# Impaired glymphatic function in idiopathic intracranial hypertension

**DOI:** 10.1093/braincomms/fcab043

**Published:** 2021-03-21

**Authors:** Per Kristian Eide, Are Hugo Pripp, Geir Ringstad, Lars Magnus Valnes

**Affiliations:** 1 Institute of Clinical Medicine, University of Oslo, N-0316 Oslo, Norway; 2 Department of Neurosurgery, Oslo University Hospital—Rikshospitalet, N-0424 Oslo, Norway; 3 Oslo Centre of Biostatistics and Epidemiology, Research Support Services, Oslo University Hospital, N-0424 Oslo, Norway; 4 Department of Radiology, Oslo University Hospital—Rikshospitalet, N-0424 Oslo, Norway

**Keywords:** idiopathic intracranial hypertension, glymphatic system, cerebrospinal fluid, MRI, pulsatile intracranial pressure

## Abstract

Idiopathic intracranial hypertension is a brain disease incorporating cerebrospinal fluid disturbance, increased intracranial pressure and visual failure, but with unknown cause. This study examined a hypothesis that glymphatic function is impaired in idiopathic intracranial hypertension patients. The MRI contrast agent gadobutrol was utilized as a cerebrospinal fluid tracer following intrathecal administration. Consecutive standardized T1 MRI acquisitions over 48 h were done to assess tracer distribution within brain of 15 idiopathic intracranial hypertension patients and 15 reference individuals who were comparable in age and gender distribution. Using FreeSurfer software, we semi-quantified tracer level in multiple brain regions as T1 MRI signal change. The tracer enriched the entire brain of idiopathic intracranial hypertension and reference subjects. In idiopathic intracranial hypertension, tracer enrichment was increased and clearance of tracer delayed from a wide range of brain regions, including both grey and white matter. Differences were most evident in frontal and temporal regions. The pulsatile intracranial pressure was measured overnight and tracer propagation in brain compared between individuals with pathological and normal pulsatile intracranial pressure. In individuals with pathological pulsatile intracranial pressure, tracer enrichment was stronger and clearance from brain delayed, particularly in regions nearby large artery trunks at the brain surface. The present *in vivo* observations provide evidence for impaired glymphatic function in several brain regions of idiopathic intracranial hypertension patients. Glymphatic failure may imply altered clearance of metabolic byproducts, which may precede neurodegeneration. Further studies are needed to characterize glymphatic failure in idiopathic intracranial hypertension.

## Teaser

Intrathecal contrast-enhanced MRI provided evidence for impaired glymphatic function in many brain regions, including both grey and white matter, of patients with idiopathic intracranial hypertension, a brain disease characterized by CSF disturbance and increased intracranial pressure of unknown cause.

## Graphical Abstract

**Graphical Abstract:**
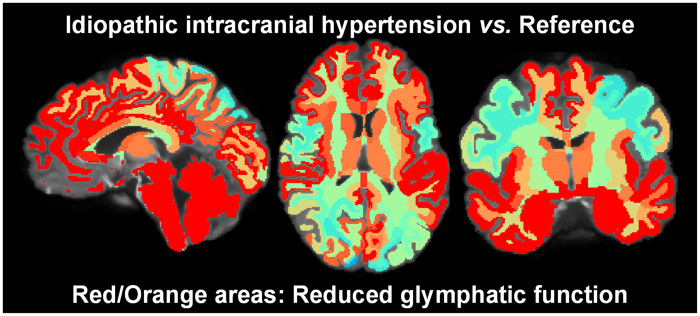

## Introduction

Idiopathic intracranial hypertension (IIH), also denoted pseudotumor cerebri and benign intracranial hypertension, is a disease characterized by elevated intracranial pressure (ICP) and visual failure, most frequently affecting overweight, fertile women.^1^^,^^2^ The typical features are headache in combination with papilledema and visual failure (obscurations, blurred vision and diplopia), though a subset of patients may suffer IIH without papilledema. Other symptoms include pulsatile tinnitus, dizziness, olfactory dysfunction and cognitive impairment.^2–6^ Typically, the disease has major impact on quality of life as well as ability to work/schooling.^7^

The cause of IIH is unknown, and disease management remains symptomatic, aiming at reducing ICP, either by weight reduction, medication to reduce cerebrospinal fluid (CSF) production (e.g. acetazolamide), or interventions such as CSF diversion (shunt) surgery, stenting stenosis of dural sinus or fenestration of the optic sheet.^1^^,^^2^^,^^6^ However, the current management options have limitations. A significant proportion of patients have lasting symptoms, often resulting in numerous hospital re-admissions. This, together with an increasing prevalence of the disease due to increasing weight in the population results in increased burden to the health care system.^8^^,^^9^ A deeper understanding of the disease is required to improve treatment.

Increased CSF pressure is one diagnostic requirement for IIH, though the composition of CSF should be normal.^1^^,^^2^ Accordingly, disturbance of CSF homeostasis resulting in increased CSF pressure is the most recognized aetiological factor in IIH. Over the last few years, our understanding of CSF homeostasis has changed, not least facilitated by the discovery of the glymphatic system in 2012.^10^ From the initial studies performed in rodents, we have learned that in humans a tracer molecule administered to the lumbar CSF enriches around large artery trunks, before enriching the entire brain in a centripetal pattern.^11^^,^^12^ Modelling have provided evidence that tracer movement is driven by convective forces in addition to diffusion.^13^ Other imaging modalities than utilizing intrathecal contrast agents have also been used to assess the human glymphatic system.^14–16^

In the dementia subtype idiopathic normal pressure hydrocephalus (iNPH) impaired clearance of tracer from CSF and brain is accompanied with abnormal pulsatile ICP,^17^ the latter also characterizing patients with IIH.^18^^,^^19^ Given the close association between vascular disease affecting arterial pulsatility and glymphatic function,^20^ recent ultrastructural observations of marked changes at the glia-neuro-vascular interface of IIH^21–24^ may indicate the presence of impaired glymphatic function in IIH.

In the present study, we tested the hypothesis that glymphatic function is impaired in IIH, which has not been investigated before. The study was carried out in patients under clinical work up of IIH and compared with a gender and age-matched group. The MRI contrast agent gadobutrol was administered intrathecally, serving as a CSF tracer. Multiphase MRI over 48 h was performed to assess the time and magnitude aspect of CSF tracer distribution within brain, as indicator of glymphatic function.

## Materials and methods

### Approvals

The study was approved by these authorities: Regional Committee for Medical and Health Research Ethics (REK) of Health Region South-East, Norway (2015/96). The Institutional Review Board of Oslo university hospital (2015/1868). The National Medicines Agency (15/04932–7). The study was registered in Oslo University Hospital Research Registry (ePhorte 2015/1868). Study participants were included after written and oral informed consent, and the study was governed by ethical standards according to the Helsinki Declaration of 1975 (and as revised in 1983).

The study excluded individuals with a history of hypersensitivity reactions to contrast agents, a history of severe allergy reactions in general and evidence of renal dysfunction. Pregnant or breastfeeding women or individuals aged <18 or >80 years were not included.

### Study design and patients

To assess CSF tracer transport, we used the MRI contrast agent gadobutrol as a CSF tracer following intrathecal injection. Standardized T1 MRI acquisitions were performed at multiple time points, including before: pre-contrast, and after 0–0.5 h, 0.5–3 h, 3–7 h (Day 1), 24 h (Day 2), 48 h (Day 3) and after 4 weeks. The distribution of tracer over time within CSF spaces and brain was compared between IIH patients and a cohort of reference (REF) subjects. The selected time points were based on our previous experience with this kind of MRI.

#### IIH patients

The IIH patients included consecutive individuals referred to the Department of Neurosurgery, Oslo University Hospital—Rikshospitalet, Oslo, Norway, from local neurological and ophthalmological departments, in whom conservative treatment had failed. The following criteria of IIH is used: Increased lumbar opening pressure (>25 mmH2O), normal CSF composition, papilledema, normal neurological examination (except for fourth cranial nerve affection) and normal MRI with exclusion of venous thrombosis.^1^

#### Reference patients

Since intrathecal gadobutrol is used off-label on clinical indication, the contrast agent is not given to healthy individuals. There was a clinical indication for intrathecal contrast-enhanced MRI in all REF subjects, as illustrated by their presence of symptoms. The REF individuals included patients under clinical work-up for tentative CSF disorders in Department of Neurosurgery, Oslo University Hospital—Rikshospitalet. In patients categorized as REF in the gMRI database, the imaging and physiological investigations provided no evidence for CSF disturbance and therefore no intervention was recommended. We here included REF individuals from our gMRI database who best matched the individual IIH patients regarding age and gender. Other selection criteria were not used.

### Monitoring of pulsatile ICP

In this institution, IIH patients undergo over-night ICP monitoring as part of assessment routine before shunt surgery, as previously described.^18^^,^^19^ The ICP is measured via solid pressure sensor (Codman ICP micro sensor placed in the parenchyma) via small burr hole, as previously described.^25^ Both the static ICP (mean ICP) and pulsatile ICP (mean ICP wave amplitude; MWA) are measured. The latter refers to the pressure changes occurring during the cardiac cycle. Based on our previous studies,^18^^,^^19^^,^^25^ we categorize the pulsatile ICP as pathological when average of MWA is above 4–5 mmHg overnight and the percentage of MWA >5 mmHg exceeds 10%.

### MRI protocol

The MRI protocol utilized a 3 Tesla Philips Ingenia MRI scanner (Philips Medical systems, Best, The Netherlands), with equal imaging protocol settings at each time point to acquire sagittal 3 D T1-weighted volume scans. These imaging parameters were used: Repetition time = ‘shortest’ (typically 5.1 ms), echo time = ‘shortest’ (typically 2.3 ms), Flip angle = 8 degrees, field of view = 256 x 256 cm and matrix = 256 x 256 pixels (reconstructed 512 x 512). Hundred and eighty-four over-contiguous (overlapping) slices with one mm thickness were automatically reconstructed to 368 slices with 0.5 mm thickness. The duration of each image acquisition was 6 min and 29 s. Slice orientation of image stacks was defined using an automated anatomy recognition protocol based on landmark detection in MRI data (SmartExam™, Philips Medical Systems, Best, The Netherlands) for each time point to secure consistency and reproducibility of the MRI slice placement and orientation.

### Intrathecal gadobutrol

The intrathecal injection of gadobutrol was done by an interventional neuroradiologist. CSF backflow from the puncture needle verified correct position of the syringe tip in the subarachnoid space. Gadobutrol was given in a dose of 0.5 mmol (0.5 ml of 1.0 mmol/ml gadobutrol; Gadovist, Bayer Pharma AG, Berlin, Germany). Patients stayed in bed until the last MRI acquisition Day 1, and thereafter were allowed free movement.

### Image post-processing

Using standardized T1 MRI sequences, the change in T1 signal provides a semi-quantitative measure of the tracer concentration since gadobutrol increases the T1 relaxation of water and hence results in higher signal intensity at the image grey scale when present in CSF or brain tissue. FreeSurfer software (version 6.0) (http://surfer.nmr.mgh.harvard.edu/) was used for segmentation, parcellation and registration/alignment of MRI time series. To assess the increase of T1 intensity caused by the tracer, we utilized the segmentation and parcellation acquired from Freesurfer; methods are previously described.^26^ A hybrid watershed/surface deformation procedure is applied to remove non-brain tissue.^27^ An automated Talairach transformation and segmentation of the subcortical white matter, deep grey matter structures (including structures such as hippocampus, amygdala, caudate, putamen and ventricles) is performed.^28^ The MR images of each patient were used to create a median template registered to the baseline, and the MR images registered to the corresponding template using a rigid transformation.^29^ The registrations were subsequently checked manually by the senior author to correct for any registration errors.

To adjust for changes in the greyscale between MRI scans, the T1 signal unit for each time point was divided by the T1 signal unit of a reference region of interest within the posterior part of the orbit (retrobulbar fat) for the respective time point. This ratio represents the *normalized T1 signal unit*s and corrects for any baseline changes of image grey scale due to automatic image scaling.

Differential maps were created by subtracting the average of the median relative increase in segmentation regions of the IIH group from the average of the median relative increase in corresponding segmentation regions of the REF group. The results were projected onto an average brain segmentation in FreeSurfer for visualization.

### Statistical analyses

Continuous data were presented as mean and standard deviation or standard error, as appropriate. Normal distribution of data was assessed in both groups. Demographic and clinical information between the groups were determined by Pearson Chi-square test for categorical data and by independent samples *t*-test for continuous data. The repeated measurements on enrichment of tracer within brain regions were assessed with linear mixed models by maximum likelihood estimation using a subject-specific random intercept and including for main regions parenchyma of brain crossed effects of brain segments. Using estimated marginal mean from the statistical model, we tested the difference between the IIH and REF groups at the different points of follow-up. The mean, standard errors and *P*-values were calculated from the linear mixed model for repeated measurements.

The statistical analysis was performed using SPSS version 26 (IBM Corporation, Armonk, NY) and Stata/SE 15.0 (StataCrop LLC, College Station, TX). Statistical significance was accepted at the 0.05 level (two-tailed).

### Data availability

The data presented in this work is available upon reasonable request.

## Results

### Patients

Demographic and clinical data about the IIH (*n* = 15) and REF (*n* = 15) cohorts are presented in Table 1. They were comparable regarding gender and age. Visual obscurations and blurred vision were more common in IIH patients. The IIH patients fulfilled the diagnostic criteria of IIH disease.^1^ Subjective cognitive disturbances were prevalent in both groups.

**Table 1:** Demographic and clinical information about the IIH and REF cohorts

	IIH (*n* = 15)		REF (*n* = 15)	*P*-value
Sex (F/M)	13/2		14/1	ns
Age (years)	35.6 ± 12.2		32.1 ± 6.5	ns
BMI (kg/m^2^)	31.1 ± 5.3		27.7 ± 6.2	ns
Symptoms reported by patient				
Headache	15 (100%)		15 (100%)	ns
Visual obscuration	8(53%)		2 (13%)	=0.02
Blurred vision	14 (93%)		4 (27%)	<0.001
Diplopia	6 (40%)		2 (13%)	ns
Pulsatile tinnitus	6 (40%)		2 (13%)	ns
Dizziness	11 (73%)		6 (40%)	ns
Cognitive disturbance	7 (47%)		10 (67%)	ns
Overnight ICP/MWA				
* N*	14		8	
Mean ICP, Average	11.6 ± 5.7		7.5 ± 3.3	ns
Mean ICP, % >15 mmHg	14.1 ± 13.8		3.4 ± 3.2	ns
MWA, Average	6.7 ± 1.8		3.7 ± 0.4	<0.001
MWA, % >5 mmHg	67.0 ± 20.0		8.8 ± 6.9	<0.001
IIH—Treatment and outcome				
VP shunt, clinical responder	10 (67%)			
Conservative without surgery	5 (33%)			

Categorical data presented as numbers (ranges in parentheses); continuous data presented as mean ± SD. Significant differences between groups were determined by Pearson Chi-square test for categorical data and by independent samples *t*-test for continuous data.

IIH = idiopathic intracranial hypertension; REF = reference patients; ICP = intracranial pressure; MWA = mean ICP wave amplitude and Ns = non-significant differences between groups.

All the 10/15 IIH patients who underwent shunt surgery with implantation of a ventricular-peritoneal shunt responded favourably with improvement of visual function. Five of fifteen IIH patients were recommended shunt surgery, but preferred conservative non-surgical treatment and had lasting symptoms.

With regard to ICP scores, the pulsatile ICP, represented by the mean ICP wave amplitude (MWA) was significantly elevated in IIH subjects.

All MRI acquisitions were performed before the ICP measurements and shunt surgeries.

Prior to diagnostic assessment, 10/15 (67%) IIH subjects did not attend work/school, 3/15 (20%) partly attended work/school. Only 2/15 (13%) were attending work/school.

### CSF tracer enrichment in CSF spaces of IIH and REF groups

**Figure 1:**
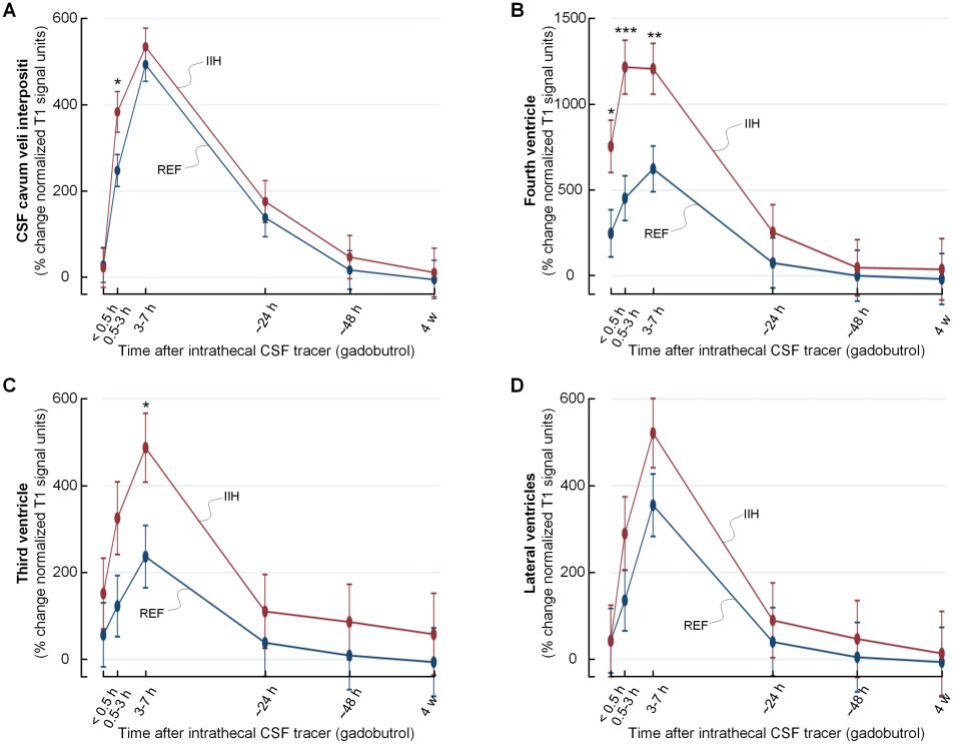
**Enrichment of tracer in intracranial CSF spaces over time in IIH and REF groups.** Following lumbar intrathecal administration, the time course of tracer level is compared between IIH and REF groups within CSF spaces segmented using FreeSurfer, including the subarachnoid space of pineal recess in **A**, fourth ventricle in **B**, third ventricle in **C** and lateral ventricles in **D**. CSF tracer levels refers to percentage change of normalized T1 signal unit ratio, and trend plots for tracer level are shown as mean ± standard error (SE). Significant differences between groups are indicated by *P < 0.05, **P < 0.01 and ***P < 0.001 (mixed model analysis).

Following intrathecal injection, the tracer rapidly enriched the CSF spaces (Video 1). The CSF space segmented by FreeSurfer was defined from the cavum veli interpositi (Supplementary Fig. 1), which is part of the subarachnoid compartment. Here, tracer enrichment was higher in IIH after 0.5–3 h, but was comparable between groups at other time points (Fig. 1A). After 3–7 h, there was similar tracer availability in CSF of both patient groups. Notably, tracer enrichment was significantly higher in fourth (Fig. 1B) and third (Fig. 1C) ventricles of IIH at 3–7 h, indicating flux of tracer toward the ventricular system early after injection. The tracer enrichment was not significant in lateral ventricles (Fig. 1D). Tracer enrichment in CSF spaces and choroid plexus is shown in Supplementary Table 1.

### Brain-wide distribution of tracer in both IIH and REF groups

**Figure 2:**
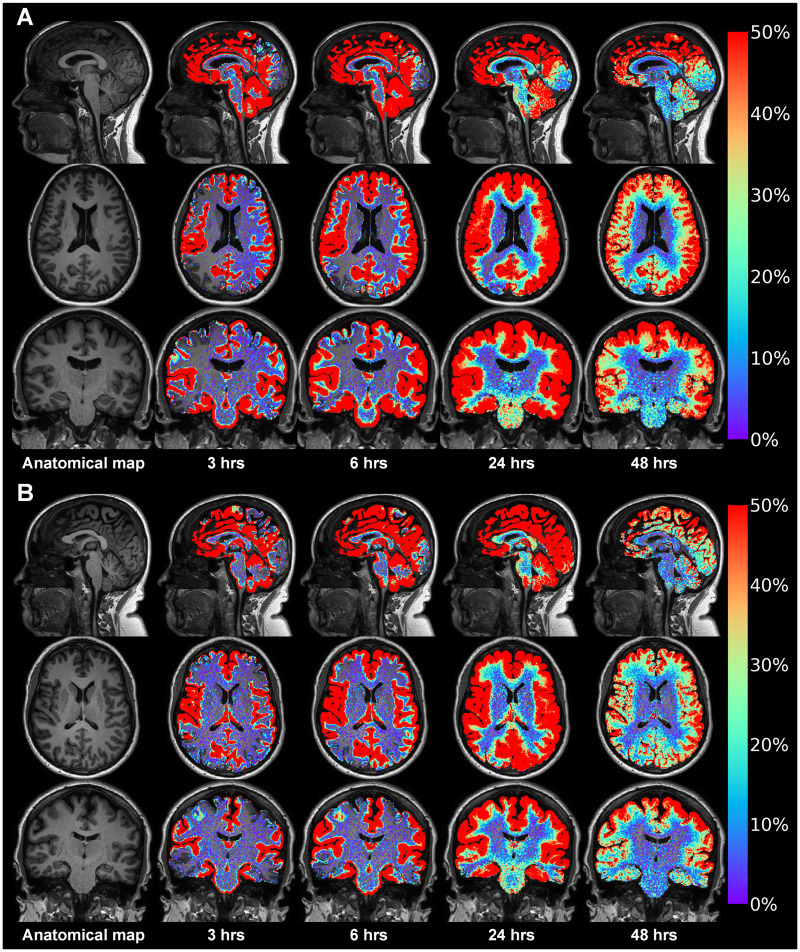
**Time course of parenchymal distribution of CSF tracer in an IIH patient and a REF subject.** gMRI from an IIH patient who complained of headache, visual obscurations, blurred vision, pulsatile tinnitus, olfactory dysfunction, dizziness and subjective cognitive impairment in **A**. The pulsatile ICP was pathological (MWA 6.6 mmHg on average), while static ICP was within normal range (average of mean ICP 12.1 mmHg). A REF patient at comparable age who complained of headache, dizziness and cognitive impairment in **B**. There was no sign of papilledema, and overnight ICP was normal. In both these patients, the CSF tracer (gadobutrol) was injected intrathecally and MRI obtained after 3 h, 6 h, 24 h and 48 h. The images are post-processed using FreeSurfer software, and showing percentage increase in normalized T1 signal over time (see colour scale to the right). Tracer in CSF spaces has been subtracted, thus only presenting tracer enrichment within brain tissue. After 3 h and 6 h, tracer enriches most strongly in brain regions adjacent to large artery trunks at the brain surface, including the medial temporal lobe close to the posterior cerebral arteries, the cingulum adjacent to the anterior cerebral arteries in the anterior interhemispheric fissure and the Sylvian fissure, where the middle cerebral artery trunks reside. Notably, the tracer enriches the extra-vascular space of the brain from outside and inward (centripetally), with peak enhancement at about 24 h.

Following enrichment of the CSF spaces, the tracer spread throughout the entire brain, as illustrated in one IIH patient (Fig. 2A) and a REF subject (Fig. 2B). Video 2 shows an animation of the tracer propagation within the brain tissue. The tracer enriched all regions of the brain, including cortical grey matter, white matter, basal ganglia and infra-tentorial regions, as further detailed in Supplementary Table 2.

**Table 2:** Enrichment of tracer within the main brain regions

Anatomical region	Group	0–0.5 h					0.5–3 h					3–7 h					_∼_ 24 h					_∼_ 48 h			
		**Mean**	**±**	**SE**	***P***		**Mean**	**±**	**SE**	***P***		**Mean**	**±**	**SE**	***P***		**Mean**	**±**	**SE**	***P***		**Mean**	**±**	**SE**	***P***
Frontal cortex (GM)	IIH	2	**±**	7	ns		35	**±**	8	0.047		99	**±**	7	ns		97	**±**	8	0.033		50	**±**	8	ns
	REF	3	**±**	7			17	**±**	7			86	**±**	7			78	**±**	8			33	**±**	8	
Frontal cortex (WM)	IIH	0	**±**	2	ns		0	**±**	2	ns		8	**±**	2	ns		31	**±**	2	0.004		24	**±**	2	0.001
	REF	–1	**±**	2			–1	**±**	2			4	**±**	2			25	**±**	2			17	**±**	2	
Temporal cortex (GM)	IIH	3	**±**	8	ns		44	**±**	8	0.002		104	**±**	8	0.014		85	**±**	8	0.006		44	**±**	8	0.047
	REF	1	**±**	8			19	**±**	8			84	**±**	8			62	**±**	8			26	**±**	8	
Temporal cortex (WM)	IIH	0	**±**	2	ns		2	**±**	3	ns		12	**±**	2	0.031		35	**±**	2	<0.001		25	**±**	2	<0.001
	REF	–2	**±**	2			–2	**±**	2			7	**±**	2			27	**±**	2			16	**±**	2	
Parietal cortex (GM)	IIH	0	**±**	6	ns		12	**±**	6	ns		43	**±**	5	ns		59	**±**	6	ns		37	**±**	6	ns
	REF	2	**±**	5			10	**±**	5			54	**±**	5			62	**±**	6			29	**±**	6	
Parietal cortex (WM)	IIH	–1	**±**	2	ns		0	**±**	2	ns		3	**±**	1	ns		18	**±**	2	ns		17	**±**	2	ns
	REF	–1	**±**	1			–1	**±**	1			3	**±**	1			20	**±**	2			14	**±**	2	
Occipital cortex (GM)	IIH	0	**±**	6	ns		13	**±**	6	ns		40	**±**	6	ns		50	**±**	6	ns		30	**±**	6	ns
	REF	2	**±**	6			10	**±**	5			42	**±**	5			44	**±**	6			21	**±**	6	
Occipital cortex (WM)	IIH	–1	**±**	2	ns		1	**±**	2	ns		5	**±**	2	ns		20	**±**	2	ns		16	**±**	2	ns
	REF	–1	**±**	2			–1	**±**	2			4	**±**	2			18	**±**	2			12	**±**	2	
Cingulate cortex (GM)	IIH	3	**±**	10	ns		57	**±**	10	0.015		126	**±**	10	ns		89	**±**	10	0.054		41	**±**	10	ns
	REF	6	**±**	10			29	**±**	10			110	**±**	10			66	**±**	10			26	**±**	10	
Cingulate cortex (WM)	IIH	0	**±**	1	ns		1	**±**	1	ns		7	**±**	1	ns		24	**±**	1	0.001		16	**±**	1	0.002
	REF	–1	**±**	1			0	**±**	1			4	**±**	1			18	**±**	1			10	**±**	1	
Corpus callosum	IIH	1	**±**	2	ns		7	**±**	2	ns		20	**±**	2	ns		22	**±**	2	0.018		11	**±**	2	0.030
	REF	–1	**±**	2			3	**±**	2			16	**±**	2			15	**±**	2			5	**±**	2	

GM = grey matter and WM = white matter. Data presented as percentage change in normalized T1 signal units over time. *P*: *P*-value (mixed model analysis) and ns = non-significant differences between groups.

### Increased tracer enrichment and delayed clearance in the IIH group

**Figure 3:**
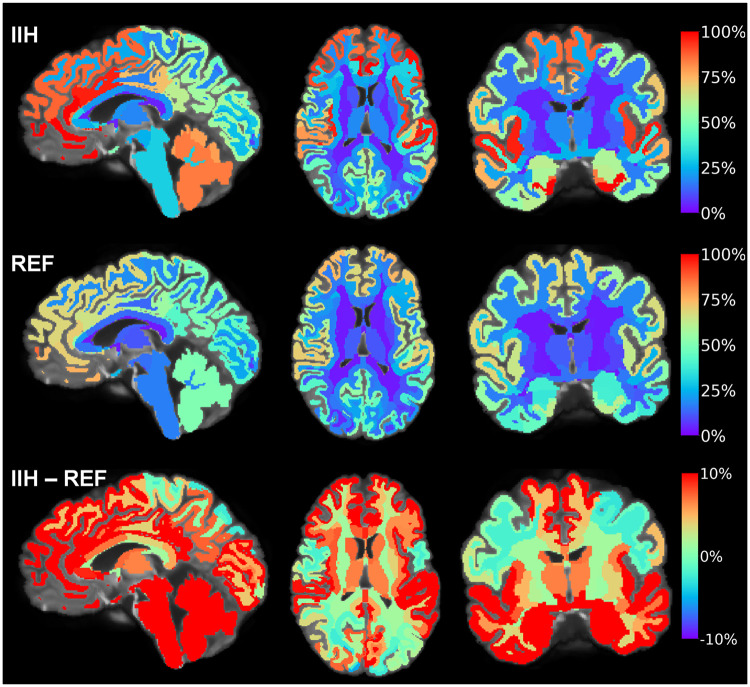
**Differences between the IIH and REF groups in enrichment of tracer within brain at 24 h.** The tracer in CSF spaces has been subtracted, showing tracer enrichment as percentage change in normalized T1 signal at 24 h from baseline. For the IIH group (upper panel), the average percentage tracer enrichment from baseline to 24 h is shown in sagittal (left), axial (middle) and coronal (right) plans. For the REF group (middle panel), the percentage tracer enrichment (normalized T1 signal) at 24 h compared to baseline is shown in and sagittal (left), axial (middle) and coronal (right) plans, with colour scale indicating percentage signal change. The difference in signal change between the cohorts (IIH minus REF groups) is shown in the lower panel. Red colour represents areas with the highest tracer levels. From these images, tracer enrichment differed most and was highest in IIH in frontal, temporal, cingulate, insular, cerebellar and brainstem regions. In this patient group, having some degree of cognitive impairment, it is notable that several of these areas correspond well with regions of tau-based neurofibrillary tangle progression in Alzheimer’s disease.^30^

In IIH patients, there was increased enhancement and reduced clearance of tracer from brain, most evidently in the frontal and temporal brain regions. In Fig. 3, the tracer enrichment from baseline at group level is shown for the IIH and REF cohorts, and presenting differences between groups at the lower panel. Notably, the increase in tracer level was most evident in frontal and temporal regions. Supplementary Fig. 2 provides a comparable illustration of differences in clearance of tracer between IIH and REF cohorts from evening Day 1 until morning Day 2.

**Figure 4:**
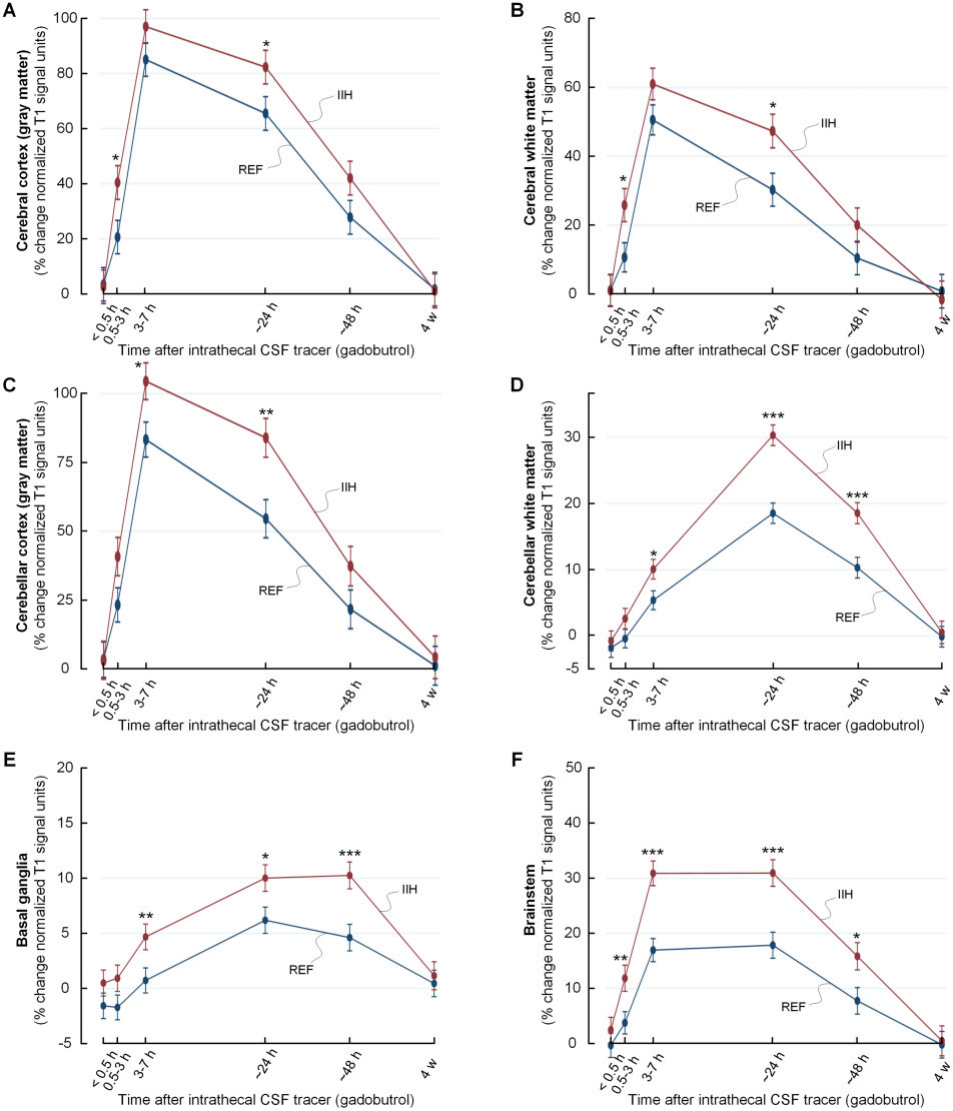
**Trend plots of tracer levels in different brain locations of the IIH and REF cohorts.** The trend plots of percentage change in tracer enrichment (normalized signal unit ratio) reveal significantly higher tracer levels in enrichment and clearance phases in the IIH than the REF group. This was shown for cerebral cortex grey matter in **A**, cerebral white matter in **B**, cerebellar cortex (grey matter) in **C**, cerebellar white matter in **D**, basal ganglia in **E** and brainstem in **F**. Trend plots are presented with mean ± standard error (SE). Statistical differences: *P < 0.05, **P < 0.01 and ***P < 0.001 (linear mixed models).

**Figure 5:**
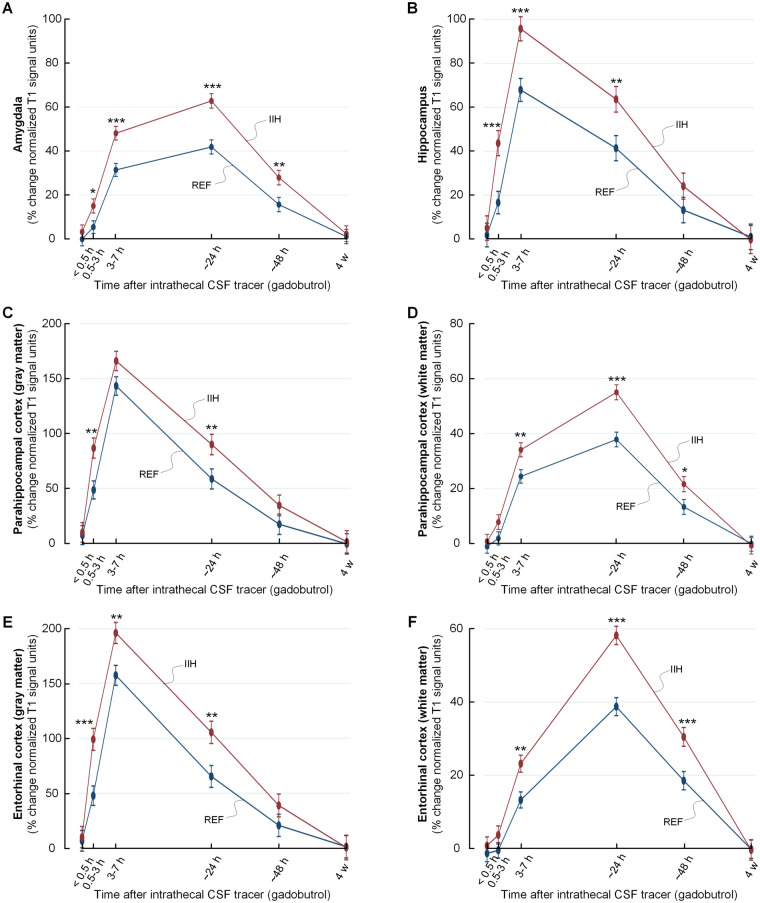
**Increased CSF tracer levels in selected limbic structures of IIH patients indicative of reduced molecular clearance.** The trend plots of percentage change in tracer enrichment (normalized signal unit ratio) reveal significantly stronger tracer enrichment in enrichment and clearance phases in IIH as compared to REF groups for several brain regions belonging to the limbic system. The regions included amygdala in **A**, hippocampus in **B**, parahippocampal cortex (grey matter) in **C**, parahippocampal white matter in **D**, entorhinal cortex (grey matter) in **E** and entorhinal subcortex (white matter) in **F**. Trend plots are presented with mean ± standard error (SE). Statistical differences: *P < 0.05, **P < 0.01 and ***P < 0.001 (linear mixed models).

**Figure 6:**
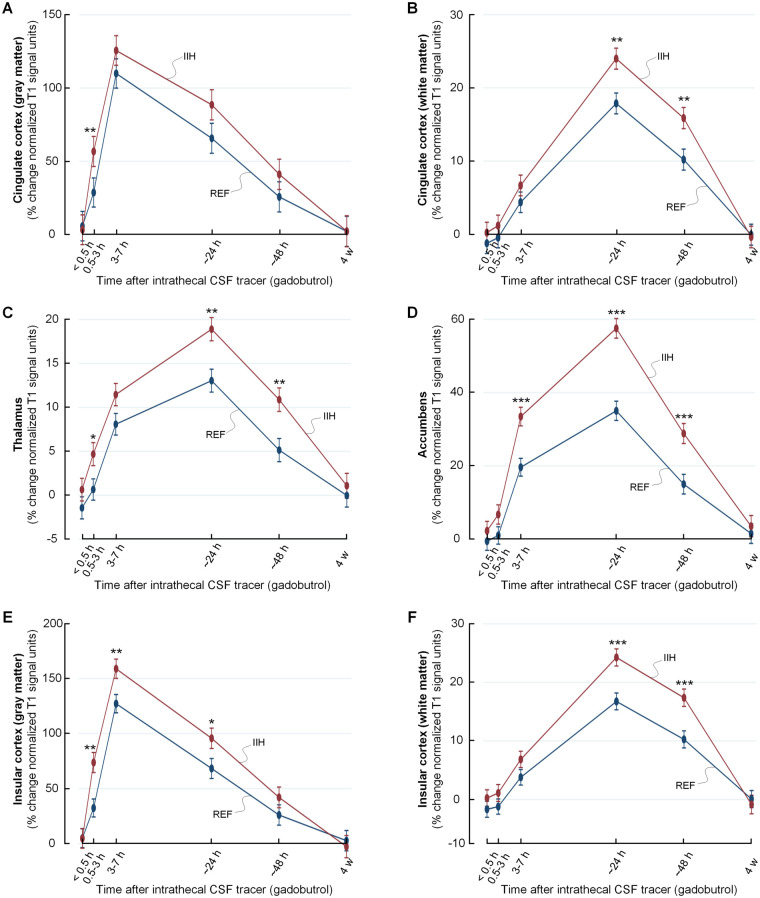
**Increased CSF tracer levels in selected limbic structures of IIH patients indicative of reduced molecular clearance.** The trend plots of percentage change in tracer enrichment (normalized signal unit ratio) reveal significantly stronger tracer enrichment in enrichment and clearance phases in IIH as compared to REF groups for several brain regions belonging to the limbic system. The regions included cingulate cortex (grey matter) in **A**, cingulate subcortex (white matter) in **B**, thalamus in **C**, accumbens area in **D**, insular cortex (grey matter) in **E** and insular subcortex (white matter) in **F**. Trend plots are presented with mean ± standard error (SE). Statistical differences: *P < 0.05, **P < 0.01 and ***P < 0.001 (linear mixed models).

As shown in Fig. 4, tracer enrichment was increased and clearance delayed in IIH patients within cerebral and cerebellar cortical grey and white matter, and within basal ganglia and brainstem. The tracer enrichment in some main brain regions are also presented in Table 2. Observations particularly for limbic structures of IIH patients are illustrated in Figs. 5 and 6. A more comprehensive list of brain regions is provided in Supplementary Table 2.

After 4 weeks, tracer enrichment in brain and CSF was normalized and without any evidence of difference between the IIH and REF groups, see Supplementary Table 3.

### Altered tracer enrichment in patients with pathological pulsatile ICP

Among the 22 of 30 study participants who had undergone overnight monitoring of ICP, overnight pulsatile ICP (MWA) was categorized as pathological in 14 (all IIH patients) and normal in 8 individuals (all REF subjects). Comparison of tracer propagation showed significantly increased tracer enrichment and delayed clearance from several brain regions in individuals with pathological MWA (Supplementary Table 4). This is further illustrated in Supplementary Fig. 3 for limbic brain structures, including amygdala, hippocampus, grey and white matter of parahippocampal cortex, grey and white matter of entorhinal cortex, grey and white matter of cingulate cortex, thalamus, accumbens area and grey and white matter of insular cortex.

## Discussion

The present results provide *in vivo* evidence for impaired glymphatic function in IIH. The brain-wide extra-vascular distribution of a CSF tracer was increased and clearance delayed in numerous brain regions of IIH patients as compared to age and gender matched REF subjects.

The REF cohort cannot be considered a material of healthy individuals, but was comparable with IIH patients regarding age and gender. They reported a high frequency of symptoms, even though no apparent CSF disturbance was verified, and therefore no intervention was advocated in these subjects.

The aetiology of IIH has remained an enigma since the disease was described by the American neurosurgeon Walter E Dandy in 1937,^31^ first denoted pseudotumor cerebri. Here, we present for the first time *in vivo* evidence for impaired glymphatic function in a consecutive series of non-selected IIH subjects. The glymphatic system refers to a peri- (or para-) vascular transport route for convective transport of fluids and solutes consisting of an influx route for CSF along arteries and an efflux route along veins, after the free mixing of CSF with solutes within the interstitial space.^10^ The convective transport of fluids and solutes along vessels and the interstitial space is thought to be driven by forces created from the arterial pulsations.^32^ This transport route enables a pathway for clearance of byproducts from brain metabolism not being actively transported through the blood-brain–barrier. Experimental data suggest that the glymphatic system becomes impaired with increasing age^33^ and a role of glymphatic failure has been suggested for various neurological and dementia diseases based on animal disease models.^34^^,^^35^ With regard to *in vivo* evidence for impaired glymphatic function in humans, the available evidence refers to the dementia subtype iNPH.^11^^,^^12^^,^^36^

The CSF tracer used here is a highly hydrophilic and stable molecule of size 604 Da that does not pass the blood-brain–barrier or enter the cells. After its entry from CSF to brain, it is thus confined to the extra-vascular compartment, i.e. the perivascular and interstitial spaces. Due to the resolution of MRI of 1 mm, we cannot conclude about the relative distribution within the perivascular and interstitial spaces, and neither differentiate between the peri-arterial and peri-venous spaces. The speed by which gadobutrol enriches the entire brain is indicative of contribution from convective forces adding to diffusion, which also a previous modelling study indicated.^13^ Transport of gadobutrol predominated by diffusion is likely, at least in the interstitial space, as previously suggested.^37^

It is of particular interest that tracer enrichment and clearance of tracer from structures susceptible to neurodegeneration in dementia, including hippocampus and entorhinal cortex, was delayed in IIH. It has previously been established that IIH subjects may suffer cognitive impairment.^3^^,^^4^ Among the present 15 IIH individuals, 7 (47%) reported subjective cognitive impairment, primarily subjectively impaired short-term memory and concentration. We previously found ultrastructural evidence for reduced post-synaptic density in IIH,^22^ which is indicative of reduced synaptic strength in these patients. In dementia disease such as Alzheimer’s, delayed clearance of toxic waste products such as amyloid-β and tau from CSF seems to be one mechanism behind neurodegeneration.^34^ It is therefore striking to note how distribution of areas with reduced molecular clearance in IIH match up with those most typically affected with amyloid-β and tau deposition in dementia diseases, including Alzheimer`s disease.^20^^,^^30^

There was no evidence of signal change in any brain region after 4 weeks, which indicate complete clearance of gadobutrol from the brain. This finding is in line with previous observations in iNPH.^36^ We have previously reported that intrathecal gadobutrol in a dose of 0.5 mmol is safe.^38^ One issue that needs to be addressed in future studies is whether dose of gadobutrol needs to be adjusted for height and weight since it would affect the intradural and brain volumes.

The group differences in parenchymal tracer enrichment may not be explained by differences in availability of tracer in CSF. We have previously shown that the enrichment of tracer in the brain heavily depends on availability of tracer within the CSF spaces.^11^^,^^36^

Given that increased CSF pressure is one diagnostic criterion for IIH,^1^^,^^2^ it is of interest that tracer enrichment in CSF of cavum veli interpositi, fourth and third ventricles differed from references the first few hours. The stronger ventricular enrichment of tracer in IIH compares with observations in iNPH,^11^^,^^17^ and adds evidence to the association between reduced intracranial compliance (as shown by increased pulsatile ICP) and ventricular reflux. Here, it is hypothesized that both reduced compliance and CSF flow taking the path of least resistance (to ventricles) may have a common cause, i.e. increased resistance within CSF efflux pathways.

We hypothesize that the tracer moves from CSF spaces into the brain via the Virchow–Robin spaces and further via the periarterial spaces, i.e. within the basement membrane. The latter covers the endothelial cells and pericytes of capillary and is surrounded by the astrocytic endfeet, allowing passage of fluids and solutes to the interstitial space via the endfeet gaps, which are about 20 nm.^39^ The size of perivascular spaces seem to increase with increasing body mass index (BMI),^40^ a hallmark of IIH.^1^ Moreover, a vascular involvement in IIH has previously been suggested, given that the prevalence of cardiovascular disease is increased in IIH.^41^^,^^42^

With regard to glymphatic failure, it should be noted that emerging evidence point at disturbance at the glia-neuro-vascular interface in IIH. From frontal cortical biopsies in IIH patients, we have shown pathological alterations in the basement membrane and pericytes of the cerebral capillaries^24^ and loss of blood-brain–barrier integrity and leakage of blood proteins to the perivascular space.^23^ Leakage of fibrinogen is pro-inflammatory, and a possible cause of the patchy astrogliosis characterizing the IIH brain.^21^ Astrogliosis is a non-specific alteration in astrocytes with loss of domain, orientation, and evidence of swelling, which may be seen in many brain diseases.^43^ A highly important anatomical feature of the brain vasculature is that the end-feet of the astrocytes completely ensheath the cerebral capillaries, which is required for maintenance of blood-brain–barrier function.^44^ Three-dimensional (3 D) electron microscopy (EM) showed bundles of mitochondria in the end-foot processes close to perivascular end-foot membrane.^39^ In IIH, we found by EM pathological mitochondria in astrocytic perivascular end-feet suggestive of altered cellular metabolism.^22^ Shortened endoplasmic-reticular-contact sites also indicated cellular metabolic failure.^22^ In IIH subjects, the astrocytic perivascular aquaporin-4 (AQP4) expression was significantly increased in light microscopy^21^ though immunogold EM showed unaltered perivascular AQP4.^23^ Higher demand on fluid exchange might result in a compensatory increase of perivascular AQP4. We suggest that in IIH alterations at the glia-neuro-vascular interface, particularly dysfunctional regulation of astrocytic endfeet that cover the capillaries, may affect perivascular solute transport and modify propagation of CSF tracer, as reported here.

We have previously reported that the pulsatile ICP is pathological in IIH, reflected by increased MWA scores, even in combination with normal static ICP (mean ICP).^18^^,^^19^ The present data further show that in patients with elevated pulsatile ICP, there was increased tracer influx and delayed tracer efflux from brain. The combination of impaired clearance of tracer and elevated pulsatile ICP compares with observations in iNPH.^17^

## Conclusion

The present study provides evidence for impaired glymphatic function in IIH patients, as revealed by increased influx and delayed clearance from parenchyma of an intrathecally administered CSF tracer. This was observed within numerous brain locations, but strikingly most in regions known susceptible to amyloid-β and tau deposition in Alzheimer’s disease, pointing to a possible mechanism behind cognitive dysfunction also in IIH. We suggest that in IIH pathological alterations at the glia-neuro-vascular interface may restrict transport of fluid and solutes along perivascular pathways and to and from interstitial tissue via astrocytic inter-endfeet gaps.

## Supplementary material

Supplementary material is available at *Brain Communications* online.

## Supplementary Material

fcab043_Supplementary_Data

## Notes

See Jansson and Iliff (https://doi.org/10.1093/braincomms/fcab094) for a scientific commentary on this article.

## Abbreviations

AQP4 =: aquaporin 4
BBB =: blood-brain-barrier
BMI =: body mass index
CSF =: cerebrospinal fluid
EM =: electron microscopy
ERC =: entorhinal cortex
gMRI =: glymphatic magnetic resonance imaging
ICP =: intracranial pressure
IIH =: idiopathic intracranial hypertension
iNPH =: idiopathic normal pressure hydrocephalus
MWA =: mean ICP wave amplitude
MRI =: magnetic resonance imaging
REF =: reference patient
