# Supplementary material for: Impaired glymphatic function in idiopathic intracranial hypertension
Source: Brain Commun. 2021 Mar 21;3(2):fcab043. doi: 10.1093/braincomms/fcab043 (PMC8253298; doi:10.1093/braincomms/fcab043)
Supplement: fcab043_Supplementary_Data [file fcab043_Supplementary_Data.zip › Supplementary Material.pdf]

Supplementary Material

Impaired glymphatic function in idiopathic intracranial hypertension

Per Kristian Eide[1,2*], Are Hugo Pripp[3], Geir Ringstad[4], Lars Magnus Valnes[2]
[1]*Institute of Clinical Medicine, Faculty of Medicine, University of Oslo, Oslo, Norway.*
[2]*Department of Neurosurgery, Oslo University Hospital – Rikshospitalet, Oslo, Norway.*
[3]*Oslo Centre of Biostatistics and Epidemiology, Research Support Services, Oslo University Hospital, Oslo, Norway.*
[4]*Department of Radiology, Oslo University Hospital- Rikshospitalet, Oslo, Norway.*

Correspondence:

Professor Per Kristian Eide, MD PhD

Department of Neurosurgery

Oslo University Hospital - Rikshospitalet

Pb 4950 Nydalen,

N-0424 Oslo, Norway

p.k.eide@medisin.uio.no

Supplementary Fig. 1

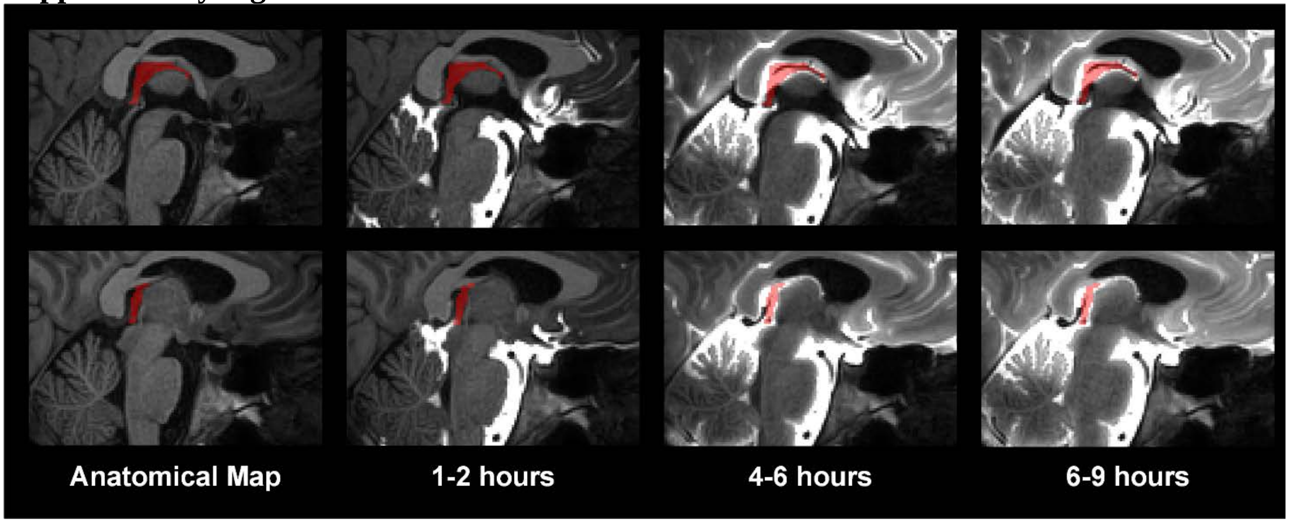

Automatic segmentation of CSF using FreeSurfer Software. The FreeSurfer Software segments CSF in the cavum veli interpositi, which is in free communication with the subarachnoid space. The images show tracer enrichment within subarachnoid spaces. The area segmented by FreeSurfer is marked with red color. All images are from one IIH patient.

Supplementary Fig. 2.

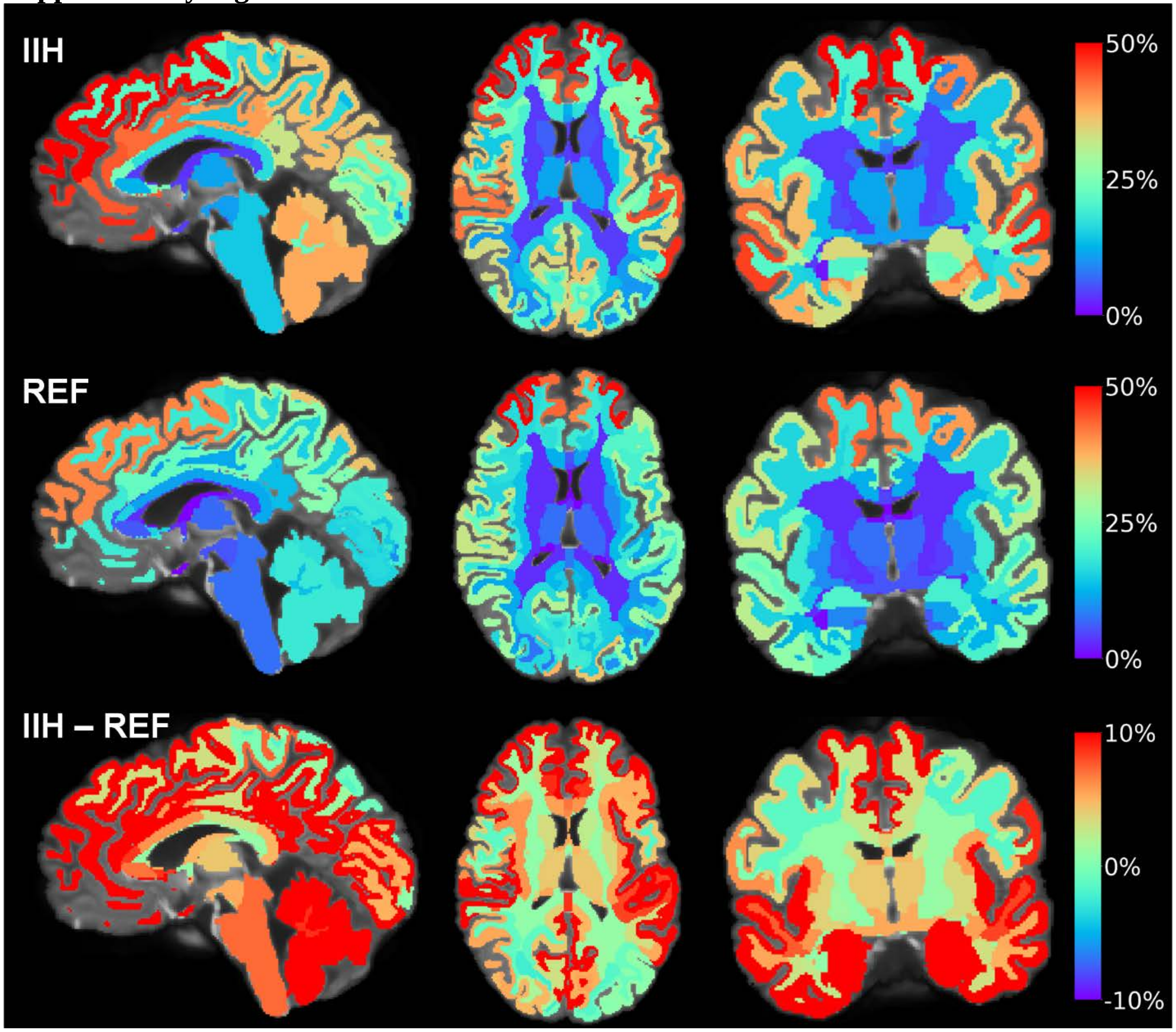

In vivo brain imaging demonstrates reduced molecular clearance in IIH from evening Day 1 until morning Day 2. Tracer enrichment in brain tissue at group level is expressed by percentage change of MRI T1 signal from evening Day 1 until morning Day 2, and shown on the color scale. The images show tracer enrichment within brain tissue, while tracer in CSF spaces have been subtracted. Upper panel: The tracer dependent signal change in brain is presented on average for the IIH group (n=15) from evening Day 1 to morning Day 2. The sagittal (left), axial (middle) and coronal (right) MRI scans are presented with the percentage signal increase indicated at the color scale. Middle panel: The average percentage signal change from tracer enrichment is presented for the REF group (n=15) from evening Day 1 to morning Day 2. Lower panel: The percentage difference in signal increase between the groups (IIH minus REF groups) is shown. It is shown by the color scale that tracer levels in brain tissue were higher in the IIH group compared to the REF group, i.e. clearance of tracer was least in the IIH group. Red color represents areas with the highest tracer enrichment. In particular, clearance of tracer in IIH was most impaired in frontal, temporal, cingulate cortical areas, close to the regions where the large arterial trunks (medial, anterior and posterior cerebral arteries) reside. Clearance also was reduced in cerebellum and brainstem.

Supplementary Fig. 3

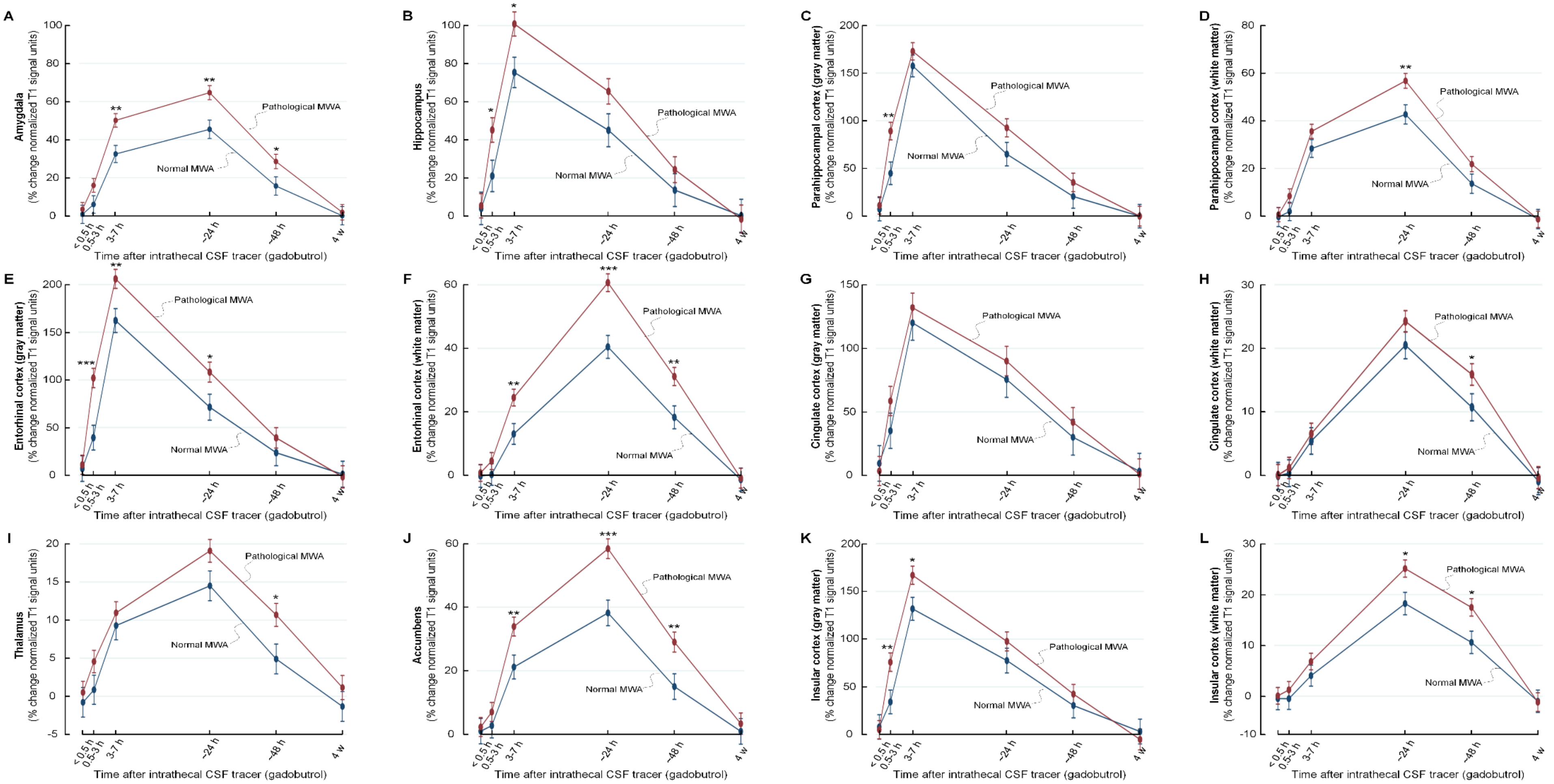

Increased CSF tracer levels in limbic structures of individuals with pathological pulsatile ICP. The trend plots of percentage change in tracer enrichment (normalized signal unit ratio) reveal significantly stronger tracer enrichment in enrichment and clearance phases of individuals with pathological pulsatile ICP (mean ICP wave amplitude; MWA; n=14) as compared to the individuals with normal MWA (n=8). The brain regions belonging to the limbic system included (A) amygdala, (B) hippocampus, (C) parahippocampal cortex (gray matter), (D) parahippocampal white matter, (E) entorhinal cortex (gray matter), (F) entorhinal cortex (white matter), (G) cingulate cortex (gray matter), (H) cingulate cortex (white matter), (I) thalamus (J) accumbens area, (K) insular cortex (gray matter), and (L) insular cortex (white matter). Trend plots are presented with mean ± standard error (SE). Statistical differences: *P<0.05, **P<0.01, ***P<0.001 (linear mixed models).

Supplementary Table 1. Enrichment of tracer within the CSF, cerebral ventricles and choroid plexus.

Anatomical region	Group	0 – 0.5 hours				0.5 - 3 hours				3 - 7 hours				~24 hours				~48 hours			
		Mean	±	SE	p	Mean	±	SE	p	Mean	±	SE	p	Mean	±	SE	p	Mean	±	SE	p
CSF	IIH	22	±	45	ns	383	±	47	=0.024	534	±	43	ns	176	±	49	ns	47	±	50	ns
	REF	29	±	40		248	±	37		493	±	38		138	±	44		17	±	45	
4th ventricle	IIH	755	±	153	=0.014	1217	±	157	<0.001	1207	±	148	=0.003	255	±	160	ns	48	±	164	ns
	REF	248	±	138		453	±	130		624	±	133		76	±	146		1	±	148	
3rd ventricle	IIH	152	±	82	ns	325	±	84	ns	488	±	79	=0.019	111	±	85	ns	86	±	87	ns
	REF	57	±	74		123	±	70		237	±	72		39	±	78		9	±	79	
Lateral ventricles	IIH	43	±	82	ns	290	±	85	ns	521	±	80	ns	90	±	86	ns	47	±	88	ns
	REF	43	±	74		136	±	70		355	±	72		40	±	79		5	±	80	
Choroid plexus	IIH	3	±	6	ns	26	±	6	ns	47	±	6	ns	23	±	6	ns	13	±	7	ns
	REF	4	±	6		18	±	6		49	±	6		16	±	6		8	±	6	

Data presented as percentage change in normalized T1 signal units over time. P: p-value (mixed model analysis); ns: non-significant differences between groups.

Supplementary Table 2. Comparison between IIH and REF groups of percentage change in tracer enrichment within brain at different time points.

		0 - 0.5 hours				0.5 - 3 hours				3 - 7 hours				~ 24 hours				~ 48 hours			
**Anatomical region**	**Group**	**Mean**	**±**	**SE**	**p**	**Mean**	**±**	**SE**	**p**	**Mean**	**±**	**SE**	**p**	**Mean**	**±**	**SE**	**p**	**Mean**	**±**	**SE**	**p**
Some main regions																					
Cerebral cortex (gray matter)	IIH	3	±	6	ns	40	±	6	0.013	97	±	6	ns	82	±	6	0.036	42	±	6	ns
	REF	3	±	6		21	±	6		85	±	6		66	±	6		28	±	6	
Cerebral white matter	IIH	1	±	5	ns	26	±	5	=0.018	61	±	5	ns	47	±	5	=0.013	20	±	5	ns
	REF	1	±	5		11	±	4		51	±	4		30	±	5		10	±	5	
Basal ganglia	IIH	0	±	1	ns	1	±	1	ns	5	±	1	=0.009	10	±	1	=0.016	10	±	1	<0.001
	REF	-2	±	1		-2	±	1		1	±	1		6	±	1		5	±	1	
Cerebellar cortex (gray matter)	IIH	3	±	7	ns	41	±	7	ns	105	±	7	0.021	84	±	7	=0.003	37	±	7	ns
	REF	4	±	7		23	±	6		83	±	6		55	±	7		22	±	7	
Cerebellar white matter	IIH	-1	±	2	ns	3	±	2	ns	10	±	2	=0.023	30	±	2	<0.001	19	±	2	<0.001
	REF	-2	±	2		0	±	1		5	±	1		19	±	2		10	±	2	
Brain Stem	IIH	2	±	2	ns	12	±	2	=0.009	31	±	2	<0.001	31	±	2	<0.001	16	±	3	0.019
	REF	0	±	2		4	±	2		17	±	2		18	±	2		8	±	2	
Limbic structures																					
Amygdala	IIH	3	±	3	ns	15	±	3	=0.026	48	±	3	<0.001	63	±	3	<0.001	28	±	3	=0.009
	REF	0	±	3		6	±	3		31	±	3		42	±	3		16	±	3	
Hippocampus	IIH	5	±	6	ns	44	±	6	<0.001	96	±	6	<0.001	64	±	6	0.006	24	±	6	ns
	REF	2	±	5		17	±	5		68	±	5		41	±	8		13	±	6	
Entorhinal cortex (gray matter)	IIH	10	±	10	ns	99	±	10	<0.001	196	±	10	=0..003	106	±	10	=0.005	39	±	10	ns
	REF	7	±	9		48	±	9		157	±	9		65	±	10		21	±	10	
Entorhinal cortex (white matter)	IIH	1	±	2	ns	4	±	3	ns	23	±	2	=0.002	58	±	3	<0.001	30	±	3	=0.001
	REF	-1	±	2		-1	±	2		13	±	2		39	±	3		19	±	3	

Parahippocampal cortex (gray matter)	IIH	10	±	9	ns	87	±	9	=0.002	166	±	9	ns	90	±	9	=0.016	35	±	10	ns
	REF	8	±	9		49	±	8		143	±	8		59	±	9		17	±	9	
Parahippocampal cortex (white matter)	IIH	1	±	3	ns	8	±	3	ns	34	±	3	=0.006	55	±	3	<0.001	22	±	3	0.033
	REF	-1	±	3		2	±	2		24	±	2		38	±	3		13	±	3	
Thalamus	IIH	1	±	1	ns	5	±	1	=0.024	12	±	1	ns	19	±	1	=0.002	11	±	1	=0.002
	REF	-1	±	1		1	±	1		8	±	1		13	±	1		5	±	1	
Cingulate cortex (gray matter)																					
Rostral anterior cingulate	IIH	6	±	13	ns	110	±	13	<0.001	212	±	13	=0.004	115	±	13	=0.032	48	±	14	ns
	REF	8	±	13		45	±	12		162	±	12		75	±	13		30	±	13	
Caudal anterior cingulate	IIH	3	±	11	ns	67	±	11	=0.021	155	±	11	=0.030	101	±	12	ns	45	±	12	ns
	REF	7	±	11		31	±	10		122	±	11		73	±	11		29	±	12	
Posterior cingulate	IIH	2	±	8	ns	24	±	8	ns	74	±	8	ns	75	±	8	ns	38	±	8	ns
	REF	4	±	8		16	±	7		75	±	7		65	±	8		27	±	8	
Isthmus cingulate	IIH	2	±	6	ns	25	±	6	ns	59	±	6	=0.016	63	±	7	ns	34	±	7	ns
	REF	3	±	6		20	±	6		80	±	6		50	±	6		19	±	7	
Insular cortex (gray matter)	IIH	5	±	9	ns	74	±	9	=0.001	159	±	9	=0.009	96	±	9	=0.034	42	±	9	ns
	REF	5	±	9		32	±	8		127	±	8		68	±	9		26	±	9	
Basal ganglia																					
Accumbens area	IIH	2	±	3	ns	7	±	3	ns	33	±	3	<0.001	58	±	3	<0.001	29	±	3	<0.001
	REF	-1	±	3		1	±	2		20	±	2		35	±	3		15	±	3	
Caudate nucleus	IIH	1	±	2	ns	3	±	2	ns	12	±	2	=0.001	11	±	2	=0.041	9	±	2	=0.021
	REF	-1	±	2		-1	±	2		3	±	2		5	±	2		3	±	2	
Putamen	IIH	1	±	1	ns	0	±	1	ns	1	±	1	ns	14	±	1	=0.012	15	±	1	<0.001
	REF	-2	±	1		-2	±	1		0	±	1		10	±	1		8	±	1	

Pallidum	IIH	0	±	1	ns	0	±	1	ns	1	±	1	ns	6	±	1	ns	7	±	1	=0.005
	REF	-2	±	1		-2	±	1		-1	±	1		4	±	1		3	±	1	
Frontal cortex (gray matter)																					
Rostral middle frontal	IIH	-1	±	6	ns	3	±	6	ns	40	±	6	ns	95	±	6	ns	59	±	7	=0.046
	REF	2	±	6		4	±	6		35	±	6		85	±	6		41	±	6	
Caudal middle frontal	IIH	-2	±	6	ns	1	±	6	ns	25	±	6	ns	57	±	6	ns	46	±	6	ns
	REF	1	±	6		2	±	5		30	±	5		66	±	6		35	±	6	
Pars opercularis	IIH	0	±	8	ns	33	±	8	ns	102	±	8	ns	92	±	8	ns	48	±	8	ns
	REF	2	±	7		18	±	7		93	±	7		72	±	8		31	±	8	
Pars orbitalis	IIH	2	±	8	ns	28	±	8	ns	108	±	8	ns	125	±	8	=0.002	60	±	9	=0.042
	REF	3	±	8		14	±	7		96	±	7		88	±	8		36	±	8	
Pars triangularis	IIH	-1	±	7	ns	26	±	7	ns	98	±	7	ns	100	±	8	=0.024	52	±	8	ns
	REF	1	±	7		13	±	6		86	±	7		77	±	7		32	±	8	
Frontal pole	IIH	3	±	12	ns	60	±	12	=0.010	172	±	12	=0.002	149	±	13	=0.006	58	±	13	ns
	REF	4	±	11		18	±	11		122	±	11		101	±	12		36	±	13	
Superior frontal	IIH	0	±	7	ns	14	±	7	ns	57	±	7	ns	94	±	7	ns	56	±	7	ns
	REF	2	±	7		8	±	7		45	±	7		81	±	7		40	±	7	
Lateral orbito-frontal	IIH	6	±	10	ns	76	±	10	=0.002	178	±	10	=0.003	117	±	10	=0.003	51	±	10	ns
	REF	6	±	9		35	±	9		140	±	9		75	±	10		29	±	10	
Medial orbito-frontal	IIH	10	±	13	ns	129	±	14	<0.001	238	±	13	=0.004	126	±	14	=0.020	51	±	14	ns
	REF	11	±	13		57	±	12		187	±	13		81	±	14		30	±	14	
Precentral	IIH	-1	±	6	ns	11	±	7	ns	46	±	6	ns	62	±	7	ns	39	±	7	ns
	REF	1	±	6		9	±	6		61	±	6		67	±	7		31	±	7	
Paracentral	IIH	0	±	6	ns	9	±	6	ns	39	±	6	ns	59	±	6	ns	34	±	6	ns
	REF	2	±	6		9	±	5		47	±	6		64	±	6		30	±	6	

Temporal cortex (gray matter)											
Inferior temporal	IIH	0 ± 4	ns	5 ± 4	ns	27 ± 3	ns	57 ± 4	=0.006	44 ± 4	=0.001
	REF	-3 ± 3		-1 ± 3		20 ± 3		43 ± 4		26 ± 4	
Middle temporal	IIH	0 ± 6	ns	13 ± 6	ns	54 ± 5	ns	79 ± 6	=0.019	48 ± 6	=0.035
	REF	-3 ± 5		1 ± 5		42 ± 5		60 ± 6		31 ± 6	
Superior temporal	IIH	2 ± 9	ns	52 ± 9	=0.018	130 ± 9	ns	100 ± 9	=0.039	47 ± 9	ns
	REF	2 ± 8		23 ± 8		110 ± 8		74 ± 9		30 ± 9	
Temporal pole	IIH	9 ± 11	ns	95 ± 12	<0.001	206 ± 11	=0.004	137 ± 12	=0.003	56 ± 12	ns
	REF	7 ± 11		40 ± 10		162 ± 11		88 ± 12		30 ± 12	
Fusiform	IIH	1 ± 4	ns	18 ± 4	ns	57 ± 4	ns	63 ± 4	=0.017	35 ± 4	=0.034
	REF	0 ± 4		9 ± 4		46 ± 4		49 ± 4		22 ± 4	
Transverse temporal	IIH	3 ± 9	ns	80 ± 9	=0.001	147 ± 9	=0.043	76 ± 10	ns	34 ± 10	ns
	REF	3 ± 9		37 ± 8		123 ± 9		56 ± 9		21 ± 9	
Parietal cortex (gray matter)											
Postcentral	IIH	-1 ± 8	ns	19 ± 9	ns	65 ± 8	ns	74 ± 9	ns	43 ± 9	ns
	REF	1 ± 8		16 ± 8		87 ± 8		78 ± 9		33 ± 9	
Superior parietal	IIH	0 ± 5	ns	2 ± 6	ns	15 ± 5	ns	50 ± 6	ns	37 ± 6	ns
	REF	2 ± 5		4 ± 5		23 ± 5		63 ± 6		35 ± 6	
Inferior parietal	IIH	0 ± 5	ns	3 ± 5	ns	20 ± 5	ns	45 ± 5	ns	33 ± 5	ns
	REF	0 ± 5		1 ± 5		22 ± 5		50 ± 5		27 ± 5	
Supramarginal	IIH	0 ± 8	ns	21 ± 8	ns	68 ± 8	ns	77 ± 9	ns	43 ± 9	ns
	REF	1 ± 8		13 ± 7		81 ± 8		75 ± 8		33 ± 9	
Precuneus	IIH	1 ± 6	ns	11 ± 6	ns	37 ± 6	ns	56 ± 6	ns	38 ± 6	ns
	REF	3 ± 6		10 ± 5		49 ± 5		60 ± 6		27 ± 6	
Pericalcarine	IIH	1 ± 6	ns	17 ± 6	ns	52 ± 6	ns	55 ± 6	ns	31 ± 6	ns
	REF	2 ± 5		12 ± 5		59 ± 5		48 ± 6		21 ± 6	

Occipital cortex (gray matter)											
Cuneus	IIH	0 ± 6	ns	13 ± 6	ns	46 ± 6	ns	60 ± 6	ns	36 ± 6	ns
	REF	2 ± 6		14 ± 5		61 ± 6		56 ± 6		25 ± 6	
Lingual	IIH	1 ± 5	ns	25 ± 5	ns	67 ± 5	ns	64 ± 5	=0.051	31 ± 5	ns
	REF	3 ± 5		16 ± 5		61 ± 5		50 ± 5		20 ± 5	
Lateral occipital	IIH	-1 ± 3	ns	1 ± 3	ns	6 ± 3	ns	26 ± 3	ns	24 ± 3	ns
	REF	-1 ± 3		-1 ± 2		3 ± 3		26 ± 3		19 ± 3	
Corpus callosum											
Anterior	IIH	1 ± 2	ns	5 ± 2	ns	19 ± 2	ns	23 ± 2	=0.003	13 ± 2	=0.024
	REF	-1 ± 2		2 ± 2		16 ± 2		15 ± 2		6 ± 2	
Mid anterior	IIH	1 ± 3	ns	8 ± 3	ns	20 ± 3	ns	20 ± 3	ns	9 ± 3	ns
	REF	-1 ± 3		2 ± 2		14 ± 3		14 ± 3		4 ± 3	
Central	IIH	1 ± 3	ns	7 ± 3	ns	19 ± 3	ns	17 ± 3	ns	10 ± 3	ns
	REF	-1 ± 3		1 ± 3		12 ± 3		12 ± 3		4 ± 3	
Mid posterior	IIH	0 ± 3	ns	9 ± 3	ns	27 ± 3	ns	23 ± 3	ns	10 ± 3	ns
	REF	-1 ± 3		4 ± 3		25 ± 3		16 ± 3		4 ± 3	
Posterior	IIH	0 ± 2	ns	4 ± 2	ns	13 ± 2	ns	25 ± 2	=0.020	13 ± 2	ns
	REF	-1 ± 2		2 ± 2		15 ± 2		18 ± 2		7 ± 2	
Optic chiasm	IIH	23 ± 12	ns	142 ± 12	<0.001	187 ± 11	=0.001	63 ± 12	ns	19 ± 13	ns
	REF	16 ± 11		75 ± 10		137 ± 10		33 ± 12		4 ± 12	
Cingulate cortex (white matter)											
Rostral anterior cingulate	IIH	1 ± 2	ns	2 ± 2	ns	12 ± 2	=0.021	35 ± 2	<0.001	23 ± 2	<0.001
	REF	-1 ± 2		0 ± 2		7 ± 2		23 ± 2		14 ± 2	
Caudal anterior cingulate	IIH	0 ± 2	ns	1 ± 2	ns	7 ± 1	ns	23 ± 2	=0.002	15 ± 2	=0.008
	REF	-1 ± 1		0 ± 1		4 ± 1		16 ± 2		9 ± 2	

Posterior cingulate	IIH	0	±	1	ns	1	±	2	ns	4	±	1	ns	18	±	2	ns	12	±	2	ns
	REF	-2	±	1		-1	±	1		3	±	1		15	±	2		9	±	2	
Isthmus cingulate	IIH	0	±	1	ns	1	±	1	ns	4	±	1	ns	20	±	1	ns	14	±	1	=0.017
	REF	-2	±	1		-1	±	1		4	±	1		17	±	1		9	±	1	
Insular cortex (white matter)	IIH	0	±	1	ns	1	±	1	ns	7	±	1	ns	24	±	2	<0.001	17	±	2	=0.001
	REF	-2	±	1		-1	±	1		4	±	1		17	±	1		10	±	2	
Frontal cortex (white matter)																					
Rostral middle frontal	IIH	-1	±	2	ns	-1	±	2	ns	1	±	1	ns	19	±	2	ns	22	±	2	=0.012
	REF	-1	±	1		-1	±	1		-1	±	1		17	±	2		16	±	2	
Caudal middle frontal	IIH	0	±	2	ns	-1	±	2	ns	0	±	1	ns	10	±	2	ns	15	±	2	ns
	REF	-1	±	1		-2	±	1		0	±	1		13	±	2		12	±	2	
Pars opercularis	IIH	0	±	2	ns	1	±	2	ns	6	±	2	ns	27	±	2	ns	23	±	2	=0.004
	REF	-2	±	2		-2	±	2		4	±	2		22	±	2		16	±	2	
Pars orbitalis	IIH	0	±	2	ns	0	±	2	ns	10	±	2	ns	49	±	2	<0.001	34	±	2	<0.001
	REF	-1	±	2		-1	±	2		6	±	2		38	±	2		22	±	2	
Pars triangularis	IIH	0	±	2	ns	0	±	2	ns	6	±	2	ns	29	±	2	ns	24	±	2	=0.002
	REF	-1	±	2		-1	±	1		3	±	2		24	±	2		17	±	2	
Frontal pole	IIH	-2	±	3	ns	1	±	3	ns	25	±	3	=0.001	69	±	3	<0.001	35	±	3	=0.017
	REF	-1	±	3		-2	±	3		11	±	3		50	±	3		24	±	3	
Superior frontal	IIH	0	±	2	ns	0	±	2	ns	2	±	2	ns	24	±	2	ns	24	±	2	=0.018
	REF	-1	±	2		-1	±	2		0	±	2		20	±	2		18	±	2	
Lateral orbitofrontal	IIH	1	±	2	ns	2	±	2	ns	11	±	2	=0.016	38	±	2	<0.001	28	±	2	<0.001
	REF	-1	±	2		-1	±	1		6	±	1		26	±	2		17	±	2	
Medial orbitofrontal	IIH	0	±	2	ns	4	±	2	ns	19	±	2	=0.009	47	±	2	<0.001	31	±	2	<0.001
	REF	0	±	2		1	±	2		12	±	2		32	±	2		20	±	2	
Precentral	IIH	0	±	2	ns	-1	±	2	ns	2	±	2	ns	15	±	2	ns	16	±	2	ns
	REF	-2	±	2		-2	±	2		2	±	2		19	±	2		14	±	2	

Paracentral	IIH	0 ± 2	ns	-1 ± 2	ns	1 ± 2	ns	16 ± 2	ns	16 ± 2	ns
	REF	-2 ± 2		-2 ± 1		0 ± 2		17 ± 2		14 ± 2	
Temporal cortex (white matter)											
Inferior temporal	IIH	-1 ± 1	ns	-1 ± 2	ns	2 ± 1	ns	17 ± 2	ns	21 ± 2	<0.001
	REF	-3 ± 1		-3 ± 1		0 ± 1		14 ± 2		13 ± 2	
Middle temporal	IIH	-1 ± 2	ns	0 ± 2	ns	3 ± 2	ns	24 ± 2	=0.032	23 ± 2	=0.001
	REF	-3 ± 2		-3 ± 2		1 ± 2		18 ± 2		15 ± 2	
Superior temporal	IIH	0 ± 2	ns	2 ± 2	ns	13 ± 2	ns	37 ± 2	=0.005	25 ± 2	=0.010
	REF	-2 ± 2		-2 ± 2		10 ± 2		29 ± 2		17 ± 2	
Temporal pole	IIH	1 ± 3	ns	4 ± 3	ns	29 ± 3	=0.016	79 ± 3	<0.001	45 ± 3	<0.001
	REF	0 ± 3		0 ± 3		19 ± 3		58 ± 3		29 ± 3	
Fusiform	IIH	-1 ± 1	ns	0 ± 1	ns	3 ± 1	ns	17 ± 1	ns	16 ± 1	=0.003
	REF	-2 ± 1		-2 ± 1		1 ± 1		14 ± 1		10 ± 1	
Transverse temporal	IIH	0 ± 2	ns	4 ± 3	ns	23 ± 2	=0.003	38 ± 3	=0.002	22 ± 3	=0.047
	REF	-2 ± 2		-1 ± 2		14 ± 2		27 ± 3		14 ± 3	
Parietal cortex (white matter)											
Postcentral	IIH	-1 ± 2	ns	1 ± 2	ns	7 ± 2	ns	27 ± 2	ns	20 ± 3	ns
	REF	-2 ± 2		-2 ± 2		9 ± 2		30 ± 2		18 ± 2	
Superior parietal	IIH	-1 ± 2	ns	-1 ± 2	ns	0 ± 2	ns	12 ± 2	ns	15 ± 2	ns
	REF	-1 ± 2		-1 ± 1		0 ± 1		17 ± 2		14 ± 2	
Inferior parietal	IIH	0 ± 2	ns	0 ± 2	ns	0 ± 2	ns	10 ± 2	ns	13 ± 2	ns
	REF	-2 ± 2		-2 ± 1		-1 ± 1		12 ± 2		11 ± 2	
Supramarginal	IIH	-1 ± 2	ns	0 ± 2	ns	3 ± 2	ns	21 ± 2	ns	19 ± 2	ns
	REF	-2 ± 2		-2 ± 2		3 ± 2		21 ± 2		16 ± 2	
Precuneus	IIH	-1 ± 1	ns	0 ± 1	ns	2 ± 1	ns	16 ± 2	ns	15 ± 2	ns
	REF	-1 ± 1		-1 ± 1		2 ± 1		19 ± 1		13 ± 2	
Pericalcarine	IIH	-1 ± 2	ns	1 ± 2	ns	5 ± 2	ns	21 ± 2	ns	17 ± 2	=0.050
	REF	-1 ± 2		-1 ± 2		5 ± 2		19 ± 2		12 ± 2	

Occipital cortex (white matter)											
Cuneus	IIH	-1 ± 2	ns	1 ± 2	ns	6 ± 2	ns	26 ± 2	ns	19 ± 2	ns
	REF	-1 ± 2		-1 ± 2		7 ± 2		24 ± 2		15 ± 2	
Lingual	IIH	-1 ± 2	ns	2 ± 2	ns	9 ± 2	ns	26 ± 2	ns	16 ± 2	=0.024
	REF	-1 ± 2		0 ± 2		8 ± 2		22 ± 2		11 ± 2	
Lateral occipital	IIH	-1 ± 1	ns	0 ± 1	ns	-2 ± 1	ns	9 ± 1	ns	12 ± 1	ns
	REF	-1 ± 1		-2 ± 1		-3 ± 1		8 ± 1		9 ± 1	

Ns: non-significant differences between groups.

Supplementary Table 3. Comparisons between IIH and REF groups of percentage change in tracer within brain tissue after 4 weeks.

	IIH			REF			Difference (IIH – REF)			
**Anatomical region**	**Mean**	**±**	**SE**	**Mean**	**±**	**SE**	**Mean**	**±**	**SE**	**p**
Some main regions of brain parenchyma										
Cerebral cortex (gray matter)	1.1	±	6.2	1.7	±	6.1	-0.6	±	8.1	0.941
Cerebral white matter	-1.7	±	5.4	0.8	±	4.9	-2.4	±	7.3	0.739
Basal ganglia	1.1	±	1.3	0.4	±	1.2	0.7	±	1.7	0.671
Cerebellar cortex (gray matter)	4.3	±	7.7	1.2	±	7.0	3.1	±	10.5	0.769
Cerebellar white matter	0.5	±	1.7	-0.1	±	1.6	0.7	±	2.3	0.779
Brain Stem	0.4	±	2.7	-0.3	±	2.4	0.7	±	3.6	0.846
Limbic structures										
Amygdala	2.4	±	3.6	1.1	±	3.2	1.3	±	4.8	0.791
Hippocampus	-0.2	±	6.4	1.0	±	5.8	-1.2	±	8.6	0.887
Entorhinal cortex (gray matter)	0.6	±	11.2	1.3	±	10.2	-0.7	±	15.1	0.965
Entorhinal cortex (white matter)	-0.4	±	2.8	-0.2	±	2.5	-0.2	±	3.8	0.954
Parahippocampal cortex (gray matter)	1.5	±	10.1	-0.4	±	9.3	1.8	±	13.7	0.893
Parahippocampal cortex (white matter)	-0.8	±	3.0	-0.1	±	2.7	-0.8	±	4.1	0.853
Thalamus	1.1	±	1.4	0	±	1.3	1.1	±	1.9	0.567
Cingulate cortex (gray matter)										
Rostral anterior cingulate	1.2	±	14.6	2.9	±	13.4	-1.8	±	19.8	0.928
Caudal anterior cingulate	1.1	±	12.6	2.9	±	11.5	-1.8	±	17.0	0.917
Posterior cingulate	1.4	±	8.8	2.3	±	8.1	-0.8	±	11.9	0.945
Isthmus cingulate	5.9	±	7.1	1.0	±	6.5	4.9	±	9.6	0.612
Insular cortex (gray matter)	-2.8	±	10.1	2.7	±	9.2	-5.4	±	13.7	0.691
Basal ganglia										
Accumbens area	3.5	±	2.9	1.5	±	2.7	2.0	±	4.0	0.612

Caudate nucleus	2.5	±	2.2	0.7	±	2.1	1.8	±	3.1	0.549
Putamen	1.2	±	1.2	1.0	±	1.1	0.3	±	1.6	0.862
Pallidum	-0.2	±	1.0	-0.2	±	0.9	0.0	±	1.4	0.993
Frontal cortex (gray matter)										
Rostral middle frontal	4.7	±	7.2	3.3	±	6.4	1.4	±	9.6	0.886
Caudal middle frontal	0.5	±	6.9	2.4	±	6.2	-2.0	±	9.2	0.831
Pars opercularis	-2.5	±	8.9	1.9	±	8.0	-4.4	±	12.0	0.714
Pars orbitalis	3.0	±	9.4	2.1	±	8.4	0.9	±	12.6	0.942
Pars triangularis	-0.8	±	8.4	2.3	±	7.5	-3.1	±	11.2	0.785
Frontal pole	8.3	±	13.9	3.8	±	12.5	4.4	±	18.9	0.812
Superior frontal	3.7	±	7.9	3.4	±	7.2	0.3	±	10.7	0.979
Lateral orbito-frontal	1.5	±	11.2	1.9	±	10.1	-0.4	±	15.1	0.978
Medial orbito-frontal	2.8	±	15.2	1.6	±	13.9	1.2	±	20.6	0.953
Precentral	-2.3	±	7.4	1.8	±	6.6	-4.1	±	9.9	0.679
Paracentral	0.4	±	6.7	2.1	±	6.1	-1.7	±	9.1	0.854
Temporal cortex (gray matter)										
Inferior temporal	2.7	±	4.1	0.5	±	3.7	2.2	±	5.5	0.689
Middle temporal	0.0	±	6.3	0.8	±	5.7	-0.8	±	8.6	0.930
Superior temporal	-2.7	±	10.0	1.5	±	9.1	-4.2	±	13.5	0.755
Temporal pole	4.6	±	13.1	3.4	±	11.9	1.1	±	17.6	0.949
Fusiform	2.4	±	4.8	0.1	±	4.4	2.3	±	6.5	0.722
Transverse temporal	-3.5	±	10.4	1.4	±	9.4	-4.9	±	14.0	0.728
Parietal cortex (gray matter)										
Postcentral	-4.9	±	9.7	1.4	±	8.7	-6.3	±	13.0	0.628
Superior parietal	1.4	±	6.2	3.2	±	5.6	-1.7	±	8.4	0.836
Inferior parietal	0.3	±	5.8	1.6	±	5.2	-1.3	±	7.8	0.865
Supramarginal	-5.6	±	9.3	0.9	±	8.5	-6.5	±	12.6	0.605
Precuneus	5.7	±	6.5	1.8	±	6.0	3.9	±	8.8	0.660
Pericalcarine	8.8	±	6.4	1.4	±	5.8	7.4	±	8.7	0.392

Occipital cortex (gray matter)				
Cuneus	6.4 ± 6.8	1.6 ± 6.2	4.8 ± 9.2	0.601
Lingual	4.8 ± 5.8	0.8 ± 5.2	4.0 ± 7.8	0.605
Lateral occipital	4.8 ± 3.0	1.4 ± 2.8	3.4 ± 4.1	0.405
Corpus callosum				
Anterior	-1.3 ± 2.2	0.2 ± 2.0	-1.5 ± 2.9	0.610
Mid anterior	-0.1 ± 3.1	0.1 ± 2.8	-0.2 ± 4.2	0.958
Central	0.3 ± 3.2	-0.4 ± 2.9	0.8 ± 4.3	0.861
Mid posterior	0.0 ± 3.7	-0.6 ± 3.3	0.6 ± 5.0	0.899
Posterior	-0.5 ± 2.3	-0.4 ± 2.1	-0.1 ± 3.1	0.975
Optic chiasm	-4.2 ± 13.9	-2.1 ± 12.3	-2.1 ± 18.5	0.913
Cingulate cortex (white matter)				
Rostral anterior cingulate	-0.2 ± 1.8	0.1 ± 1.7	-0.4 ± 2.5	0.881
Caudal anterior cingulate	-0.4 ± 1.6	0.4 ± 1.5	-0.7 ± 2.2	0.743
Posterior cingulate	-0.4 ± 1.6	-0.2 ± 1.5	-0.2 ± 2.2	0.915
Isthmus cingulate	-0.3 ± 1.5	-0.4 ± 1.4	0.1 ± 2.0	0.944
Insular cortex (white matter)	-0.9 ± 1.6	0.1 ± 1.5	-0.9 ± 2.2	0.667
Frontal cortex (white matter)				
Rostral middle frontal	0.2 ± 1.7	0.3 ± 1.5	-0.1 ± 2.3	0.969
Caudal middle frontal	-0.7 ± 1.7	0.3 ± 1.5	-1.0 ± 2.3	0.651
Pars opercularis	-1.5 ± 2.0	0.3 ± 1.8	-1.7 ± 2.7	0.520
Pars orbitalis	0.4 ± 2.6	0.1 ± 2.3	0.4 ± 3.4	0.919
Pars triangularis	-1.3 ± 1.9	0.2 ± 1.7	-1.4 ± 2.5	0.569
Frontal pole	2.6 ± 3.6	0.8 ± 3.2	1.7 ± 4.8	0.720
Superior frontal	0.4 ± 2.0	0.5 ± 1.8	-0.1 ± 2.7	0.982
Lateral orbitofrontal	0.0 ± 1.8	0.0 ± 1.6	0.0 ± 2.4	0.992

Medial orbitofrontal	0.7	±	2.2	0	±	2.0	0.7	±	3.0	0.811
Precentral	-1.3	±	1.9	0.3	±	1.7	-1.6	±	2.6	0.524
Paracentral	-0.4	±	1.8	0.2	±	1.6	-0.7	±	2.4	0.787
Temporal cortex (white matter)										
Inferior temporal	-0.8	±	1.6	-0.3	±	1.5	-0.5	±	2.2	0.827
Middle temporal	-0.9	±	1.9	-0.3	±	1.7	-0.6	±	2.6	0.819
Superior temporal	-1.6	±	2.3	0.1	±	2.1	-1.6	±	3.1	0.602
Temporal pole	0.5	±	3.5	0.5	±	3.1	0.0	±	4.6	0.997
Fusiform	-0.5	±	1.4	-0.5	±	1.3	0.1	±	1.9	0.979
Transverse temporal	-1.6	±	2.7	0.2	±	2.5	-1.8	±	3.7	0.623
Parietal cortex (white matter)										
Postcentral	-2.1	±	2.7	0.1	±	2.4	-2.2	±	3.6	0.540
Superior parietal	0	±	1.8	0.3	±	1.6	-0.3	±	2.4	0.892
Inferior parietal	-0.4	±	1.7	-0.1	±	1.6	-0.3	±	2.3	0.912
Supramarginal	-1.7	±	2.1	-0.3	±	1.9	-1.4	±	2.8	0.620
Precuneus	0.6	±	1.6	0	±	1.5	0.6	±	2.1	0.788
Pericalcarine	2.2	±	1.9	0.1	±	1.7	2.2	±	2.6	0.389
Occipital cortex (white matter)										
Cuneus	2.0	±	2.2	0.4	±	2.0	1.7	±	3.0	0.576
Lingual	0.8	±	1.8	-0.2	±	1.6	1.0	±	2.4	0.689
Lateral occipital	1.5	±	1.5	0.3	±	1.4	1.3	±	2.1	0.541

P: p-value; ns: non-significant differences between groups.

Supplementary Table 4. CSF tracer levels depending on pulsatile ICP (MWA levels) in some brain regions.

Anatomical region	MWA-Group	0 - 0.5 hours				0.5 - 3 hours				3 - 7 hours				~ 24 hours				~ 48 hours			
		Mean	±	SE	p	Mean	±	SE	p	Mean	±	SE	p	Mean	±	SE	p	Mean	±	SE	p
Some main regions																					
Frontal cortex (gray matter)	Pathological	1	±	8	ns	36	±	8	ns	104	±	8	ns	98	±	8	ns	50	±	8	ns
	Normal	7	±	10		21	±	10		88	±	10		91	±	10		39	±	10	
Frontal cortex (white matter)	Pathological	-1	±	2	ns	1	±	2	ns	8	±	2	ns	32	±	2	ns	25	±	2	=0.029
	Normal	0	±	2		0	±	2		4	±	2		28	±	2		18	±	2	
Temporal cortex (gray matter)	Pathological	3	±	9	ns	46	±	9	=0.011	109	±	9	=0.015	87	±	9	ns	44	±	9	ns
	Normal	3	±	11		18	±	11		83	±	11		67	±	11		29	±	11	
Temporal cortex (white matter)	Pathological	0	±	3	ns	2	±	3	ns	13	±	3	ns	36	±	3	=0.009	25	±	3	=0.008
	Normal	-1	±	3		-2	±	3		7	±	3		28	±	3		17	±	3	
Parietal cortex (gray matter)	Pathological	0	±	6	ns	13	±	6	ns	45	±	6	ns	59	±	6	ns	37	±	6	ns
	Normal	6	±	8		12	±	8		54	±	8		69	±	8		32	±	8	
Parietal cortex (white matter)	Pathological	-1	±	2	ns	0	±	2	ns	3	±	2	ns	18	±	2	ns	16	±	2	ns
	Normal	0	±	2		-1	±	2		3	±	2		22	±	2		14	±	2	
Occipital cortex (gray matter)	Pathological	1	±	6	ns	14	±	6	ns	42	±	6	ns	51	±	6	ns	31	±	6	ns
	Normal	3	±	7		10	±	7		44	±	7		48	±	7		21	±	7	
Occipital cortex (white matter)	Pathological	-1	±	2	ns	1	±	2	ns	5	±	2	ns	20	±	2	ns	16	±	2	ns
	Normal	-1	±	2		-1	±	2		4	±	2		20	±	2		11	±	2	
Brainstem	Pathological	3	±	3	ns	12	±	3	=0.032	32	±	3	=0.001	32	±	3	=0.005	16	±	3	ns
	Normal	0	±	4		3	±	3		18	±	3		19	±	4		7	±	4	
Basal ganglia	Pathological	0	±	1	ns	1	±	1	ns	3	±	1	ns	10	±	1	ns	10	±	1	=0.002
	Normal	-1	±	2		-1	±	2		2	±	2		6	±	2		4	±	2	
Limbic structures																					
Amygdala	Pathological	3	±	4	ns	16	±	4	ns	50	±	4	=0.002	65	±	4	=0.002	29	±	4	=0.036
	Normal	1	±	5		6	±	5		33	±	5		45	±	5		16	±	5	

Hippocampus	Pathological	5	±	6	ns	45	±	7	=0.021	101	±	6	=0.013	66	±	7	ns	24	±	7	ns
	Normal	4	±	9		21	±	8		75	±	8		45	±	9		14	±	9	
Entorhinal cortex (gray matter)	Pathological	11	±	10	ns	102	±	10	<0.001	206	±	10	=0..006	108	±	11	=0.032	39	±	11	ns
	Normal	7	±	13		40	±	13		162	±	13		71	±	14		24	±	14	
Entorhinal cortex (white matter)	Pathological	1	±	3	ns	5	±	3	ns	25	±	3	=0.006	61	±	3	<0.001	31	±	3	=0.005
	Normal	0	±	4		0	±	3		13	±	3		41	±	4		18	±	4	
Parahippocampal cortex (gray matter)	Pathological	11	±	9	ns	89	±	9	=0.003	173	±	9	ns	93	±	10	ns	35	±	10	ns
	Normal	7	±	13		45	±	12		158	±	12		65	±	12		21	±	12	
Parahippocampal cortex (white matter)	Pathological	1	±	3	ns	9	±	3	ns	36	±	3	ns	57	±	3	=0.006	22	±	3	ns
	Normal	0	±	4		2	±	4		28	±	4		43	±	4		14	±	4	
Thalamus	Pathological	1	±	2	ns	5	±	2	ns	11	±	2	ns	19	±	2	ns	11	±	2	=0.019
	Normal	-1	±	2		1	±	2		9	±	2		15	±	2		5	±	2	
Insular cortex (gray matter)	Pathological	5	±	10	ns	76	±	10	=0.008	167	±	10	=0.022	98	±	10	ns	42	±	10	ns
	Normal	8	±	13		34	±	12		132	±	12		78	±	13		31	±	13	
Insular cortex (white matter)	Pathological	0	±	2	ns	1	±	2	ns	7	±	2	ns	25	±	2	=0.013	18	±	2	=0.014
	Normal	-1	±	2		-1	±	2		4	±	2		18	±	2		11	±	2	
Cingulate cortex (gray matter)	Pathological	4	±	12	ns	59	±	12	ns	132	±	12	ns	90	±	12	ns	42	±	12	ns
	Normal	10	±	14		35	±	14		120	±	14		76	±	14		30	±	14	
Cingulate cortex (white matter)	Pathological	0	±	2	ns	1	±	2	ns	7	±	2	ns	24	±	2	ns	16	±	2	=0.041
	Normal	0	±	2		0	±	2		5	±	2		21	±	2		11	±	2	
Optic chiasm	Pathological	24	±	13	ns	142	±	13	=0.002	189	±	13	=0.041	64	±	14	ns	18	±	14	ns
	Normal	19	±	17		77	±	16		148	±	16		37	±	18		7	±	18	

MWA: Mean ICP wave amplitude. Ns: non-significant differences between groups.
