# Supplementary material for: Impaired glymphatic function in idiopathic intracranial hypertension
Source: Brain Commun. 2021 Mar 21;3(2):fcab043. doi: 10.1093/braincomms/fcab043 (PMC8253298; doi:10.1093/braincomms/fcab043)
Supplement: fcab043_Supplementary_Data [file fcab043_Supplementary_Data.zip › Review_files.zip!/Original Submission-Authors Response.pdf]

Manuscript ID BRAINCOM-2020-422 - Response to reviewers with reference to manuscript with changes highlighted

Reviewer #1:

General comment #1:

The authors of this manuscript provide important insights into the potential involvement of the glymphatic system in idiopathic intracranial hypertension (IIH). Their study investigates CSF clearance of intrathecally administered gadolinium-based contrast agent through a serial MRI study, a method first used in animal studies of the glymphatic system but not, until this study, applied to the study of IIH in humans. They compare a cohort of IIH with an age and gender matched cohort of patients with suspected non-IIH cerebrospinal fluid disorders through serial MRI, first obtained prior to the administration of contrast and then up to 4 weeks post-injection of a single dose of contrast. They present compelling evidence that there are differences between their cohorts, with delayed clearance of CSF from certain regions of the brain in the IIH cohort, and in particular in those patients with pathological increases in pathological pulsatile ICP.

Overall this is a strong manuscript of importance for our understanding of role of the glymphatic system. A few minor suggestions and questions for the authors are as follows.

Answer: We thank the reviewer for this positive assessment of our work.

Specific comment #1:

1) For the 10/15 IIH patients that eventually underwent shunt surgery, were any of those surgeries performed prior to 48 hour MRI or the 4 week MRI, or were all performed after the end of the study? If any were performed prior to the last MRI time point, it would be important to mention how many in the text.

Answer:

This is an important aspect that has been commented on (page 9, para 6). All MRI acquisitions were obtained before other invasive procedures (ICP and shunt).

Specific comment #2:

2) In Supplementary Figure 1, the authors should indicate in either the figure or the figure legend what the difference is between the top row and the bottom row. From information in the text, this appears to top: IIH and bottom: REF, as in many of their other figures, but clarification in the figure or figure legend (as with their other figures) would help the reader.

Answer:

We thank the reviewer for this comment, it has now been clarified in the figure legend of Suppl Fig 1 that all images are from the same IIH patient. We did not intend to compare IIH with REF, just to illustrate the segmentation by FreeSurfer.

Specific comment #3:

3) On Page 14, Line 3, there is a typo in the spelling of "glymphatic"

Answer:

Has been corrected

Specific comment #4:

4) The references for the Eide, Hasan-Olive, Hansson, and Enger J Neurosci Res paper and the Eide, Pripp, and Ringstad 2020 Brain Communications paper are not fully listed.

Answer:

The references have been updated.

Reviewer #2:

General comment #1:

The authors have made an interesting study with a title of "Impaired glymphatic function in idiopathic intracranial hypertension". The needed work has clearly been significant and important. Although, I did find some minor/major concerns during my review. These concerns are listed down below. Possible page and line numbering was done according to PDF that was provided. The more major comments are listed first and after more minor ones:

Answer:

We sincerely thank the reviewer for a thorough review of our work and thoughtful comments.

Specific comment #1:

- *I noticed that TR and TE times are changing between scans. Just for curiosity, how so? Is there not a way to fix those times for all subjects, so there wouldn't need to make any adjustments afterwards (Page 8, lines 10-17)? Even if this is done as described, there still is some possibly differences left between scans? Correct me if I am wrong, but if I have understood MRI physics correct before, shouldn't there be differences in some extent still? I.e. the percentual change between scans is little different in subject-wise and group-wise -> 1% =/= 1%, but more like 1%~1%. Or? If so, I think they should be addressed properly, how this affect gadobutrol results interpretation and possible margins for results etc? Of course, this shouldn't affect the results, but is still a technicality. Additionally, is there some reason to mention the acquisition time (6min29s), if so, please tell why*

Answer:

We appreciate the thoughts by the reviewer. At our Philips scanner, TR and TE are both routinely set to "shortest" (possible) at the console for the acquisition of the 3D T1-volume scan. We have found no reason to override this and have indeed made efforts to reduce the impact of baseline changes in grey scale between scans. As commented in page 8, para 2, we normalized the T1 signal units since changes in image grey scale that may occur between acquisitions. As described in the MRI protocol section, the MRI acquisitions utilized "equal imaging protocol settings at each time point to acquire sagittal 3D T1-weighted volume scans" (page 7, para 2). Since all image settings were kept unchanged, there should be no systematic differences affecting group comparisons. The minor error introduced by the MRI modality equally affects both groups and causes the variation between groups, but cannot explain our reported differences between groups.

We reported acquisition time as it says something about the clinical feasibility of the method, moreover, we have experienced this is information typically required by journals.

Specific comment #2:

- *How does the scanning time point affect? Those 0.5-3h and 3-7h scannings in particular? I mean, how divided the subjects were inside particular time point? I.e. how many subjects were scanned after 30min and how many after 2.5h? Or after 3.5h and 6.5h when they are in same group nevertheless? Is there any statistical differences between study groups, REF group mean time for scanning was ~1h and with IIH it was ~2h etc? Enrichment vs time linear correlation within particular time group basically? Is there any way to address this similarly like with TE and TR before? Additionally, how "accurate" 24h and 48h are?*

Answer: The selected time points were based on our previous experience, as now commented on (page 6, para 1). The included participants are all patients, and MRI acquisitions were part of diagnostic assessment. Since it is part of a clinical setup, exact time points for scanning are impossible, but the variation distributes equally between groups. As can be seen from trend plots of normalized T1 signal change, the differences between groups were rather constant over time, which would not be expected if time point for scanning affected the observations.

Specific comment #3:

- *How much does person size matter (especially head, brain and CSF spaces) when using same amount gadobutrol for each one? You said in your text "We have previously shown that the enrichment of tracer in the brain heavily depends on availability of tracer within the CSF spaces", thus it felt like this should be addressed too when interpreting results?*

Answer:

We thank the reviewer for this important comment, and has commented on this aspect in the Discussion (page 12, para 4). At this point, we have not examined how weight and height affects CSF tracer enrichment.

Specific comment #4:

- *You have said that groups are comparable with age and gender, that they are. However, which one is more important? For example, if you have 25 year old female patient, do you take 30 year old female or 25 year old male for REF group if exaggerated?*

Answer:

It has been commented that age and gender matching was performed at the individual level (page 6, para 3).

Specific comment #5:

- *I felt like the abstract was little unorganized. Could it be structured better?*

Answer:

The abstract has been somewhat reorganized.

Specific comment #6:

- *Which statistical test were used? In "Statistical analyses" section there were something, but the main info for these came from figure/table legends. Thus, I think that they should be added in corresponding section too?*

Answer:

The section on Statistics has been updated (page 8, last para).

Specific comment #7:

- *In page 11, lines 21-24, authors cite Mestre et al with "The convective transport of fluids and solutes along vessels and the interstitial space is thought to be driven by forces created from the arterial pulsations (Mestre et al., 2018)." This is correct of course, however, there are other forces than cardiac pulsations to affect CSF convection, mainly respiration e.g. "Dreha-Kulaczewski S, Joseph AA, Merboldt K-D, et al. Inspiration Is the Major Regulator of Human CSF Flow. J Neurosci 2015;35(6):2485–2491.", "Delaidelli A, Moiraghi A. Respiration: A New Mechanism for CSF Circulation? J. Neurosci. 2017;37(30):7076–7078.", "Kiviniemi V, Wang X, Korhonen V, et al. A Ultra-fast magnetic resonance encephalography of physiological brain activity - Glymphatic pulsation mechanisms? Journal of Cerebral Blood Flow and Metabolism 2016;36(6):1033–1045." or "Chen L, Beckett A, Verma A, Feinberg DA.*

Answer:

The reviewer here highlights a topic that is currently debated in the scientific literature, namely the relative role of convective forces versus diffusion for molecular transport in the brain. We feel it is beyond the scope of this paper to discuss this matter, but have commented in the second paragraph page 12 that both mechanisms may be involved. Presently, several papers provide support to the concept of convective forces behind paravascular molecular transport, while diffusion seems to be more prominent in the interstitial tissue. The exact mechanisms are, however, not decisive for the present conclusions.

Specific comment #8:
Dynamics of respiratory and cardiac CSF motion revealed with real-time simultaneous multi-slice EPI velocity phase contrast imaging. Neuroimage 2015;122:281–287.". So, one could say that CSF convection is more or less combination of vasomotion, respiration and cardiac physiology.
Answer:
The mechanisms behind tracer movement (and relative importance of respiratory versus cardiac induced CSF motion) is an interesting debate; our group also have recently published about this research question (Vinje et al, SciRep, 2019, Valnes et al, SciRep, 2020, and Daversin-Catty et al, PlosOne, 2020). We have, however, decided not to include a discussion on this interesting matter since it would lengthen the manuscript, not being highly relevant for this paper, and without affecting the conclusions.

Specific comment #9:
- *In introduction authors mention that disease need to be understand better for better treatments. With given results, is there any hypothetical suggestions for future treatments?*

Answer:
We feel it would be premature to speculate about future treatments based on the present observations. That said, a deeper understanding of the underlying pathophysiology is a requirement for future medical treatment. We have therefore included some comments on the role of vascular disease and glymphatic function in the context of IIH (page 5, para 2).

Specific comment #10:
- *From page 4, lines 43 onwards(and in discussion too), authors cite their own previous, and very commendable articles and results, however while reading these sections I started to question myself, is there other groups that have got similar results? Or are you only group that does this kind of research?*

Answer:
We have now referred to other studies assessing the human glymphatic system utilizing various other imaging techniques than based on intrathecal contrast agents (page 4, last para and page 5, first para).

Specific comment #11:
- Page 11, lines 28-31, "The glymphatic system is shown to be impaired with increasing age *(Kress et al., 2014) and in diseases, e.g. dementia disease and after traumatic brain injury (Reeves et al., 2020), but has mostly been studied experimentally in rodents.", the Reeves et al. study was indeed dementia study, however it is dementia+iNPH, not sheer dementia per se, and it is not particular TBI citation. Thus, I would suggest that you add references (e.g. " Rasmussen, M. K., Mestre, H. & Nedergaard, M. The glymphatic pathway in neurological disorders. Lancet Neurol. 17, 1016–1024 (2018), Tuovinen, T., Kananen, J., Rajna, Z. et al. The variability of functional MRI brain signal increases in Alzheimer's disease at cardiorespiratory frequencies. Sci Rep 10, 21559 (2020). https://doi.org/10.1038/s41598-020-77984-1 or Da Mesquita, S., Fu, Z. & Kipnis, J. The meningeal lymphatic system: a new player in neurophysiology. Neuron 100, 375–388 (2018)") or rephrase the sentence so that it would be accurate.*

Answer:
We agree that our sentence "The glymphatic system is shown..." is less accurate as in vivo evidence for impaired glymphatic function in neurological disease only has been shown for iNPH. Experimental data from animals are available for various models of disease. The sentence has been rephrased, as suggested (page 11, para 4).

Specific comment #12:

\- *There were several unclear abbreviations, i.e. not opened in Abbreviations list or in text. Sometimes, the abbreviation was opened, but it was used only once in same sentence where it was opened in text. Of course, I am familiar with most of them, but not everyone are. Additionally, as journal is instruction, abbreviations should be listed and opened in text. I am not too fond of using abbreviations if there is only once or twice where it is needed->text becomes "code". Next are examples from the text:*

o Page 8, line 8, LMV, not opened, not explained, please define, and there is no actual need for abbreviation, because mentioned only once.

o Page 13, line 21, EM, same explanation as previous

o Page 13, line 24&32, TEM, same explanation as previous

o Page 13, line 29, LM, same explanation as previous

o Page 13, line 21, EM, same explanation as previous

o Page 4, line 7, BIH, only mention in brackets and the list, no need

o Page 4, line 14, IIHWOP, only mention in brackets and the list, no need

o Page 11, line 50, ERC, appears in the list, but only mention in abbreviated form without opening.

o Table 1+page 12, line 51, BMI, of course well know, but appears in list, thus should be opened in the text/legend too, as annoying as it is…

o PTC, two mentions, in intro and discussion, in both there were also opened form, so why abbreviate anymore?

o Page 8, line 12, ROI, only mention in brackets and the list, no need

o Just plain MRI abbreviation was listed by the journal as an abbreviation that doesn't need to be defined, of course it could, but no "actual" need.

Answer:

We are sorry for these shortcomings and sincerely thank the reviewer for drawing our attention to these errors. They have now all been corrected.

Specific comment #13:

\- *Table 1 text, I understand continuous data representation with mean+std, however, std is not same as (minimum – maximum) that was mentioned too. I think, it would be appropriate to delete min-max mentioning from the text.*

Answer:

The reviewer is right, we have corrected.

Specific comment #14:

\- *Page 10, line 17, "Suppl. Fig. 1 provides a---" -> Suppl. Fig. 2 provides*

Answer:

Has been corrected.

Specific comment #15:

\- *Different hyphen page 6 lines 7 & 26*

Answer:

Has been corrected.

Specific comment #16:

\- *Two different form in the text: Oslo University Hospital vs. Oslo university hospital*

Answer:

Has been corrected.

Specific comment #17:
*-	Page 7, line 33, "Patients were kept flat until the last--", it is probably just me, but somehow I feel that sentence could use some rephrasing.*
Answer:
We agree, the sentence has been modified.
