# Supplementary material for: Impaired glymphatic function in idiopathic intracranial hypertension
Source: Brain Commun. 2021 Mar 21;3(2):fcab043. doi: 10.1093/braincomms/fcab043 (PMC8253298; doi:10.1093/braincomms/fcab043)
Supplement: fcab043_Supplementary_Data [file fcab043_Supplementary_Data.zip › Review_files.zip!/Original Submission-Decision Letter.pdf]

Original Submission – Decision Letter

18-Jan-2021

Dear Dr. Eide,

Thank you for your recent submission BRAINCOM-2020-422
entitled "Impaired glymphatic function in idiopathic intracranial hypertension" to Brain Communications.

Your manuscript has been reviewed, and while the reviewers had largely positive comments, they have some constructive critiques that I think you could address in a revision of the manuscript. Therefore, I invite you to respond to the reviewers' comments and revise your manuscript. The reviewers' comments are included at the bottom of this message.

In addition to the reviewers' comments, please note our editorial comments added below to comply with our requirements for presentation of scientific data at Brain Communications.

Please format your revised manuscript according to our Instructions to Authors https://academic.oup.com/braincomms/pages/General_Instructions – a summary of key points is attached to help with this.

To revise your manuscript, click the link below or log into https://mc.manuscriptcentral.com/braincom and enter your Author Centre, where you will find your manuscript title listed under "Manuscripts with Decisions." Under "Actions," click on "Create a Revision." Your manuscript number has been appended to denote a revision.

*** PLEASE NOTE: This is a two-step process. After clicking on the link, you will be directed to a webpage to confirm. ***

https://mc.manuscriptcentral.com/braincom?URL_MASK=7055098b5f2045fba72513e55403ad26

Please upload a revised manuscript with the changes you have made shown either with "track changes" or in coloured text.

When submitting your revised manuscript, you will be asked to respond to the reviewer(s) comments. Please be as specific as possible in your response to all of the comments.

The editorial team at Brain Communications also aims to increase transparency in scientific publishing and to demonstrate the value that peer review brings to the scientific process. Please therefore consider whether you are willing to have reviewer comments on your paper and your response published as supplementary data with the paper if it is accepted. This is entirely optional and will only be done if both the authors and reviewers agree. When submitting your revision you will have the opportunity to update the answer you entered previously.

IMPORTANT: Your original files are available to you when you upload your revised manuscript. Please delete any redundant files before completing the submission.

Because we are trying to facilitate timely publication of manuscripts submitted to Brain Communications, we would appreciate your revised manuscript as soon as possible. If it is not possible for you to resubmit within 3 months, the option to submit a revision will expire, and we would then consider your revised paper as a new submission. However, if you need a little more time, please get in touch and we can extend this deadline for you.

Once again, thank you for submitting your manuscript to Brain Communications and I look forward to receiving your revision.

Yours sincerely,
Prof. Tara Spires-Jones
Editor, Brain Communications

Reviewer(s)' Comments to Author:

Reviewer: 1

Comments to the Author

The authors of this manuscript provide important insights into the potential involvement of the glymphatic system in idiopathic intracranial hypertension (IIH). Their study investigates CSF clearance of intrathecally administered gadolinium-based contrast agent through a serial MRI study, a method first used in animal studies of the glymphatic system but not, until this study, applied to the study of IIH in humans. They compare a cohort of IIH with an age and gender matched cohort of patients with suspected non-IIH cerebrospinal fluid disorders through serial MRI, first obtained prior to the administration of contrast and then up to 4 weeks post-injection of a single dose of contrast. They present compelling evidence that there are differences between their cohorts, with delayed clearance of CSF from certain regions of the brain in the IIH cohort, and in particular in those patients with pathological increases in pathological pulsatile ICP.

Overall this is a strong manuscript of importance for our understanding of role of the glymphatic system. A few minor suggestions and questions for the authors are as follows.

1) For the 10/15 IIH patients that eventually underwent shunt surgery, were any of those surgeries performed prior to 48 hour MRI or the 4 week MRI, or were all performed after the end of the study? If any were performed prior to the last MRI time point, it would be important to mention how many in the text.
2) In Supplementary Figure 1, the authors should indicate in either the figure or the figure legend what the difference is between the top row and the bottom row. From information in the text, this appears to top: IIH and bottom: REF, as in many of their other figures, but clarification in the figure or figure legend (as with their other figures) would help the reader.
3) On Page 14, Line 3, there is a typo in the spelling of "glymphatic"
4) The references for the Eide, Hasan-Olive, Hansson, and Enger J Neurosci Res paper and the Eide, Pripp, and Ringstad 2020 Brain Communications paper are not fully listed.

Reviewer: 2 (Janne Kananen)

Comments to the Author
The authors have made an interesting study with a title of "Impaired glymphatic function in idiopathic intracranial hypertension". The needed work has clearly been significant and important. Although, I did find some minor/major concerns during my review. These concerns are listed down below. Possible page and line numbering was done according to PDF that was provided. The more major comments are listed first and after more minor ones:

- I noticed that TR and TE times are changing between scans. Just for curiosity, how so? Is there not a way to fix those times for all subjects, so there wouldn't need to make any adjustments afterwards (Page 8, lines 10-17)? Even if this is done as described, there still is some possibly differences left between scans? Correct me if I am wrong, but if I have understood MRI physics correct before, shouldn't there be differences in some extent still? I.e. the percentual change between scans is little different in subject-wise and group-wise -> 1% =/= 1%, but more like 1%~1%. Or? If so, I think they should be addressed properly, how this affect gadobutrol results interpretation and possible margins for results etc? Of course, this shouldn't affect the results, but is still a technicality. Additionally, is there some reason to mention the acquisition time (6min29s), if so, please tell why

- How does the scanning time point affect? Those 0.5-3h and 3-7h scannings in particular? I mean, how divided the subjects were inside particular time point? I.e. how many subjects were scanned after 30min and how many after 2.5h? Or after 3.5h and 6.5h when they are in same group nevertheless? Is there any statistical differences between study groups, REF group mean time for scanning was ~1h and with IIH it was ~2h etc? Enrichment vs time linear correlation within particular time group basically? Is there any way to address this similarly like with TE and TR before? Additionally, how "accurate" 24h and 48h are?

- How much does person size matter (especially head, brain and CSF spaces) when using same amount gadobutrol for each one? You said in your text "We have previously shown that the enrichment of tracer in the brain heavily depends on availability of tracer within the CSF spaces", thus it felt like this should be addressed too when interpreting results?

- You have said that groups are comparable with age and gender, that they are. However, which one is more important? For example, if you have 25 year old female patient, do you take 30 year old female or 25 year old male for REF group if exaggerated?

- I felt like the abstract was little unorganized. Could it be structured better?

- Which statistical test were used? In "Statistical analyses" section there were something, but the main info for these came from figure/table legends. Thus, I think that they should be added in corresponding section too?

- In page 11, lines 21-24, authors cite Mestre et al with "The convective transport of fluids and solutes along vessels and the interstitial space is thought to be driven by forces created from the arterial pulsations (Mestre et al., 2018)." This is correct of course, however, there are other forces than cardiac pulsations to affect CSF convection, mainly respiration e.g. "Dreha-Kulaczewski S, Joseph AA, Merboldt K-D, et al. Inspiration Is the Major Regulator of Human CSF Flow. J Neurosci 2015;35(6):2485–2491.", "Delaidelli A, Moiraghi A. Respiration: A New Mechanism for CSF Circulation? J. Neurosci. 2017;37(30):7076–7078.", "Kiviniemi V, Wang X, Korhonen V, et al. A Ultra-fast magnetic resonance encephalography of physiological brain activity - Glymphatic pulsation mechanisms? Journal of Cerebral Blood Flow and Metabolism 2016;36(6):1033–1045." or "Chen L, Beckett A, Verma A, Feinberg DA. Dynamics of respiratory and cardiac CSF motion revealed with real-time simultaneous multi-slice EPI velocity phase contrast imaging. Neuroimage 2015;122:281–287.". So, one could say that CSF convection is more or less combination of vasomotion, respiration and cardiac physiology.

- In introduction authors mention that disease need to be understand better for better treatments. With given results, is there any hypothetical suggestions for future treatments?

- From page 4, lines 43 onwards(and in discussion too), authors cite their own previous, and very commendable articles and results, however while reading these sections I started to question myself, is there other groups that have got similar results? Or are you only group that does this kind of research?

- Page 11, lines 28-31, "The glymphatic system is shown to be impaired with increasing age (Kress et al., 2014) and in diseases, e.g. dementia disease and after traumatic brain injury (Reeves et al., 2020), but has mostly been studied experimentally in rodents.", the Reeves et al. study was indeed dementia study, however it is dementia+iNPH, not sheer dementia per se, and it is not particular TBI citation. Thus, I would suggest that you add references (e.g. " Rasmussen, M. K., Mestre, H. & Nedergaard, M. The glymphatic pathway in neurological disorders. Lancet Neurol. 17, 1016–1024 (2018), Tuovinen, T., Kananen, J., Rajna, Z. et al. The variability of functional MRI brain signal increases in Alzheimer's disease at cardiorespiratory frequencies. Sci Rep 10, 21559 (2020). https://doi.org/10.1038/s41598-020-77984-1 or Da Mesquita, S., Fu, Z. & Kipnis, J. The meningeal lymphatic system: a new player in neurophysiology. Neuron 100, 375–388 (2018)") or rephrase the sentence so that it would be accurate.

- There were several unclear abbreviations, i.e. not opened in Abbreviations list or in text. Sometimes, the abbreviation was opened, but it was used only once in same sentence where it was opened in text. Of course, I am familiar with most of them, but not everyone are. Additionally, as journal is instruction, abbreviations should be listed and opened in text. I am not too fond of using abbreviations if there is only once or twice where it is needed->text becomes "code". Next are examples from the text:

o Page 8, line 8, LMV, not opened, not explained, please define, and there is no actual need for abbreviation, because mentioned only once.
o Page 13, line 21, EM, same explanation as previous
o Page 13, line 24&32, TEM, same explanation as previous
o Page 13, line 29, LM, same explanation as previous
o Page 13, line 21, EM, same explanation as previous
o Page 4, line 7, BIH, only mention in brackets and the list, no need
o Page 4, line 14, IIHWOP, only mention in brackets and the list, no need
o Page 11, line 50, ERC, appears in the list, but only mention in abbreviated form without opening.
o Table 1+page 12, line 51, BMI, of course well know, but appears in list, thus should be opened in the text/legend too, as annoying as it is…
o PTC, two mentions, in intro and discussion, in both there were also opened form, so why abbreviate anymore?
o Page 8, line 12, ROI, only mention in brackets and the list, no need
o Just plain MRI abbreviation was listed by the journal as an abbreviation that doesn't need to be defined, of course it could, but no "actual" need.

- Table 1 text, I understand continuous data representation with mean+std, however, std is not same as (minimum – maximum) that was mentioned too. I think, it would be appropriate to delete min-max mentioning from the text.

- Page 10, line 17, "Suppl. Fig. 1 provides a---" -> Suppl. Fig. 2 provides

- Different hyphen page 6 lines 7 & 26

- Two different form in the text: Oslo University Hospital vs. Oslo university hospital

- Page 7, line 33, "Patients were kept flat until the last--", it is probably just me, but somehow I feel that

sentence could use some rephrasing.

Editorial Office comments:

-You have supplied a thumbnail when originally submitting to Brain. Brain Communications uses graphical abstracts. Please confirm whether your thumbnail is suitable or whether you would like to supply something different?
A graphical abstract is a visual representation of the central finding or methodology of the paper. It will be displayed in the article and in the table of contents, and used to promote your article on Twitter. Authors should strive to make the graphical abstract informative, interesting, visually appealing, and straightforward (no caption needed, no abbreviations except gene/protein names). Please upload it as a separate high resolution image file (tiff preferred). The technical requirements for graphical abstracts are:
-size of the submitted image: 1200 pixels square at 300dpi
-font of the text: Arial 12-16 points (smaller fonts will not be legible online)

- please, spell out the abbreviations included in the graphical abstract.

-Please include a paragraph about data availability and a paragraph about statistical analysis in the 'Materials and Methods' section.
