# Supplementary material for: Impaired glymphatic function in idiopathic intracranial hypertension
Source: Brain Commun. 2021 Mar 21;3(2):fcab043. doi: 10.1093/braincomms/fcab043 (PMC8253298; doi:10.1093/braincomms/fcab043)
Supplement: fcab043_Supplementary_Data [file fcab043_Supplementary_Data.zip › Review_files.zip!/Original Submission.pdf]

OXFORD UNIVERSITY PRESS | Brain Communications

Impaired glymphatic function in idiopathic intracranial hypertension

Journal:	*Brain Communications*
Manuscript ID	BRAINCOM-2020-422
Manuscript Type:	Original Article
Date Submitted by the Author:	29-Dec-2020
Complete List of Authors:	Eide, Per; Oslo universitetssykehus Rikshospitalet, Neurosurgery; University of Oslo Faculty of Medicine, Neurosurgery<br>Pripp, Are; Oslo University Hospital, Oslo Center of Biostatistics and Epidemiology<br>Ringstad, Geir; Oslo University Hospital, Radiology and Nuclear Medicine<br>Valnes, Lars; Oslo University Hospital, Neurosurgery
Keywords:	Idiopathic intracranial hypertension, pseudotumor cerebri, benign intracranial hypertension, glymphatic system, cerebrospinal fluid, magnetic resonance imaging

SCHOLARONE™
Manuscripts

Title page

Impaired glymphatic function in idiopathic intracranial hypertension

Per Kristian Eide[1,2*], Are Hugo Pripp[3], Geir Ringstad[4], Lars Magnus Valnes[2]
[1]Institute of Clinical Medicine, Faculty of Medicine, University of Oslo, Oslo, Norway.
[2]Department of Neurosurgery, Oslo University Hospital – Rikshospitalet, Oslo, Norway.
[3]Oslo Centre of Biostatistics and Epidemiology, Research Support Services, Oslo University Hospital, Oslo, Norway.
[4]Department of Radiology, Oslo University Hospital- Rikshospitalet, Oslo, Norway.

For Review Only

Running title: Impaired glymphatic function in IIH

Word count: Title (69 characters), Running head (35 characters), Abstract (241 words), Total (3432 words), References (40).

Figures: 5

Tables: 2

Supplementary material: Video (2), Figures (3), Tables (4)

Corresponding author:

Professor Per Kristian Eide, MD PhD
Department of Neurosurgery
Oslo University Hospital - Rikshospitalet
Pb 4950 Nydalen,

Phone: +47 91649419

Fax: +47-23074310

N-0424 Oslo, Norway

p.k.eide@medisin.uio.no

Abstract

Idiopathic intracranial hypertension (IIH) is a brain disease incorporating cerebrospinal fluid (CSF) disturbance, increased intracranial pressure (ICP) and visual failure, but with unknown cause. This study examined a hypothesis that glymphatic function is impaired in IIH subjects. The magnetic resonance imaging (MRI) contrast agent gadobutrol was utilized as a CSF tracer following intrathecal administration. Consecutive standardized T1 MRI acquisitions over 48 hours were done to assess tracer distribution within brain of 15 IIH patients and 15 reference (REF) individuals who were comparable in age and gender distribution. Using FreeSurfer software, we semi-quantified tracer level in multiple brain regions as T1 MRI signal change. The pulsatile ICP was measured overnight and tracer propagation in brain compared between individuals with pathological and normal pulsatile ICP.

The tracer enriched the entire brain of IIH and REF subjects. In IIH, tracer enrichment was increased and clearance of tracer delayed from a wide range of brain regions, including both gray and white matter. Differences were most evident in frontal and temporal regions. Moreover, in individuals with pathological pulsatile ICP, tracer enrichment was stronger and clearance from brain delayed, particularly in regions nearby large artery trunks at the brain surface.

The present in vivo observations provide evidence for impaired glymphatic function in several brain regions of IIH patients. Glymphatic failure may imply altered clearance of metabolic byproducts, which may precede neurodegeneration. Further studies are needed to characterize glymphatic failure in IIH.

Key words: Idiopathic intracranial hypertension; pseudotumor cerebri; benign intracranial hypertension; glymphatic system; neurodegeneration; cerebrospinal fluid; magnetic resonance imaging; pulsatile intracranial pressure; wave amplitude.

For Review Only

Abbreviations:

AQP4	Aquaporin 4
BBB	Blood-brain-barrier
BIH	Benign intracranial hypertension
BMI	Body mass index
CSF	Cerebrospinal fluid
ERC	Entorhinal cortex
gMRI	Glymphatic Magnetic Resonance Imaging
ICP	Intracranial pressure
IIH	Idiopathic intracranial hypertension
IIHWOP	Idiopathic intracranial hypertension without papilledema
iNPH	Idiopathic normal pressure hydrocephalus
MWA	Mean ICP wave amplitude
MRI	Magnetic resonance imaging
PTC	Pseudotumor cerebri
REF	Reference patient
ROI	Region of interest

For Review Only

Introduction

Idiopathic intracranial hypertension (IIH), also denoted pseudotumor cerebri (PTC) and benign intracranial hypertension (BIH), is a disease characterized by elevated intracranial pressure (ICP) and visual failure, most frequently affecting overweight, fertile women (Ball and Clarke, 2006, Mollan *et al.*, 2018). The typical features are headache in combination with papilledema and visual failure (obscurations, blurred vision and diplopia), though a subset of patients may suffer IIH without papilledema (IIHWOP). Other symptoms include pulsatile tinnitus, dizziness, olfactory dysfunction, and cognitive impairment (Ball and Clarke, 2006, Kharkar *et al.*, 2011, Kunte *et al.*, 2013, Yri *et al.*, 2014, Mollan *et al.*, 2016). Typically, the disease has major impact on quality of life as well as ability to work/schooling (Kleinschmidt *et al.*, 2000).

The cause of IIH is unknown, and disease management remains symptomatic, aiming at reducing ICP, either by weight reduction, medication to reduce cerebrospinal fluid (CSF) production (e.g. acetazolamide), or interventions such as CSF diversion (shunt) surgery, stenting stenosis of dural sinus or fenestration of the optic sheet (Ball and Clarke, 2006, Mollan *et al.*, 2016, Mollan *et al.*, 2018). However, the current management options have limitations. A significant proportion of patients have lasting symptoms, often resulting in numerous hospital re-admissions. This, together with an increasing prevalence of the disease due to increasing weight in the population results in increased burden to the health care system (Friesner *et al.*, 2011, Mollan *et al.*, 2019). A deeper understanding of the disease is required to improve treatment.

Increased CSF pressure is one diagnostic requirement for IIH, though the composition of CSF should be normal (Ball and Clarke, 2006, Mollan *et al.*, 2018). Accordingly, disturbance of CSF homeostasis resulting in increased CSF pressure is the most recognized etiological factor in IIH. Over the last few years, our understanding of CSF homeostasis has changed, not least facilitated by the discovery of the glymphatic system in 2012 (Iliff *et al.*, 2012). From the initial studies performed in rodents, we have learned that in humans a tracer molecule administered to the lumbar CSF distributes throughout the entire brain (Ringstad *et al.*, 2017, Ringstad *et al.*, 2018). Typically, the tracer enriches around large artery trunks, before enriching the brain in a centripetal pattern (Ringstad *et al.*, 2017, Ringstad *et al.*, 2018). Modelling have provided evidence that tracer movement is driven by convective forces in addition to diffusion (Valnes *et al.*, 2020). In the dementia subtype idiopathic normal pressure hydrocephalus (iNPH) impaired clearance of tracer from CSF and brain is accompanied with abnormal pulsatile ICP (Eide,

For Review Only

2020), the latter also characterizing patients with IIH (Eide and Kerty, 2011, Fric and Eide, 2017).

In the present study, we tested a hypothesis that glymphatic function is impaired in IIH, which has not been investigated before. The study was carried out in patients under clinical work up of IIH and compared with a gender and age-matched group. The magnetic resonance imaging (MRI) contrast agent gadobutrol was administered intrathecally, serving as a CSF tracer. Multiphase MRI over 48 hours was performed to assess the time and magnitude aspect of CSF tracer distribution within brain, as indicator of glymphatic function.

For Review Only

Materials and Methods

Approvals

The study was approved by these authorities: Regional Committee for Medical and Health Research Ethics (REK) of Health Region South-East, Norway (2015/96). The Institutional Review Board of Oslo university hospital (2015/1868). The National Medicines Agency (15/04932-7). The study was registered in Oslo University Hospital Research Registry (ePhorte 2015/1868). Study participants were included after written and oral informed consent, and the study was governed by ethical standards according to the Helsinki Declaration of 1975 (and as revised in 1983).

The study excluded individuals with a history of hypersensitivity reactions to contrast agents, a history of severe allergy reactions in general, and evidence of renal dysfunction. Pregnant or breastfeeding women or individuals aged <18 or >80 years were not included.

Study design and patients

To assess CSF tracer transport, we used the MRI contrast agent gadobutrol as a CSF tracer following intrathecal injection. Standardized T1 MRI acquisitions were performed at multiple time points, including before : Pre-contrast, and after 0-0.5 hours, 0.5-3 hours, 3-7 hours (Day 1), 24 hours (Day 2), 48 hours (Day 3), and after 4 weeks. The distribution of tracer over time within CSF spaces and brain was compared between IIH patients and a cohort of reference (REF) subjects.

IIH patients.

The IIH patients included consecutive individuals referred to the Department of Neurosurgery, Oslo University Hospital - Rikshospitalet, Oslo, Norway, from local neurological and ophthalmological departments, in whom conservative treatment had failed. The following criteria of IIH is used: Increased lumbar opening pressure (>25 mmH2O), normal CSF composition, papilledema, normal neurological examination (except for 4th cranial nerve affection) and normal MRI with exclusion of venous thrombosis (Mollan *et al.*, 2018).

Reference (REF) patients.

Since intrathecal gadobutrol is used off-label on clinical indication, the contrast agent is not given to healthy individuals. There was a clinical indication for intrathecal contrast-enhanced MRI in all REF subjects, as illustrated by their presence of symptoms. The REF individuals included patients under clinical work-up for tentative CSF disorders in Department of Neurosurgery, Oslo university hospital – Rikshospitalet. In patients categorized as REF in the gMRI database, the imaging and physiological investigations provided no evidence for CSF disturbance and therefore no intervention was recommended. We here included REF individuals from our gMRI database who best matched the IIH cohort regarding age and gender. Other selection criteria were not used.

For Review Only

Monitoring of pulsatile intracranial pressure

In this institution, IIH patients undergo over-night intracranial pressure (ICP) monitoring as part of assessment routine before shunt surgery, as previously described (Eide and Kerty, 2011, Fric and Eide, 2017). The ICP is measured via solid pressure sensor (Codman ICP micro sensor placed in the parenchyma) via small burr hole, as previously described (Eide and Sorteberg, 2010). Both the static ICP (mean ICP) and pulsatile ICP (mean ICP wave amplitude; MWA) are measured. The latter refers to the pressure changes occurring during the cardiac cycle. Based on our previous studies (Eide and Sorteberg, 2010, Eide and Kerty, 2011, Fric and Eide, 2017), we categorize the pulsatile ICP as pathological when average of MWA is above 4-5 mmHg overnight and the percentage of MWA >5 mmHg exceeds 10%.

MRI protocol

The MRI protocol utilized a 3 Tesla Philips Ingenia MRI scanner (Philips Medical systems, Best, The Netherlands), with equal imaging protocol settings at each time point to acquire sagittal 3D T1-weighted volume scans. These imaging parameters were used: repetition time = “shortest” (typically 5.1 ms), echo time = “shortest” (typically 2.3 ms), Flip angle = 8 degrees, field of view = 256 x 256 cm and matrix = 256 x 256 pixels (reconstructed 512 x 512). Hundred and eighty-four over-contiguous (overlapping) slices with one mm thickness were automatically reconstructed to 368 slices with 0.5 mm thickness. The duration of each image acquisition was 6 minutes and 29 seconds. Slice orientation of image stacks was defined using an automated anatomy recognition protocol based on landmark detection in MRI data (SmartExam^{tm}, Philips Medical Systems, Best, The Netherlands) for each time point to secure consistency and reproducibility of the MRI slice placement and orientation.

Intrathecal gadobutrol

The intrathecal injection of gadobutrol was done by an interventional neuroradiologist. CSF backflow from the puncture needle verified correct position of the syringe tip in the subarachnoid space. Gadobutrol was given in a dose of 0.5 mmol (0.5 ml of 1.0 mmol/ml gadobutrol; Gadovist, Bayer Pharma AG, Berlin, Germany). Patients were kept flat until the last MRI acquisition Day 1, and thereafter allowed free movement.

Image post-processing

Using standardized T1 MRI sequences, the change in T1 signal provides a semi-quantitative measure of the tracer concentration since gadobutrol increases the T1 relaxation of water and hence results in higher signal intensity at the image gray scale when present in CSF or brain tissue. FreeSurfer software (version 6.0) (http://surfer.nmr.mgh.harvard.edu/) was used for segmentation, parcellation and registration/alignment of MRI time series. To assess the increase of T1 intensity caused by the tracer, we utilized the segmentation and parcellation acquired from Freesurfer; methods are previously described (Fischl, 2012). A hybrid watershed/surface deformation procedure is applied to remove non-brain tissue (Segonne *et al.*, 2004). An automated Talairach transformation and segmentation of the subcortical white matter, deep gray matter structures (including structures such as hippocampus, amygdala, caudate, putamen and

For Review Only

ventricles) is performed (Fischl *et al.*, 2002). The MR images of each patient were used to create a median template registered to the baseline, and the MR images registered to the corresponding template using a rigid transformation (Reuter *et al.*, 2012). The registrations were subsequently checked manually by LMV to correct for any registration errors.

To adjust for changes in the grayscale between MRI scans, the T1 signal unit for each time point was divided by the T1 signal unit of a reference region of interest (ROI) within the posterior part of the orbit (retrobulbar fat) for the respective time point. This ratio represents the *normalized T1 signal unit*s and corrects for any baseline changes of image gray scale due to automatic image scaling.

Differential maps were created by subtracting the average of the median relative increase in segmentation regions of the IIH group from the average of the median relative increase in corresponding segmentation regions of the REF group. The results were projected onto an average brain segmentation in FreeSurfer for visualization.

Data availability

The data presented in this work is available upon reasonable request.

Statistical analyses

Continuous data were presented as mean and standard deviation or standard error, as appropriate. Normal distribution of data was assessed in both groups. The repeated measurements were assessed with linear mixed models by maximum likelihood estimation using a subject-specific random intercept and including for main regions parenchyma of brain crossed effects of brain segments. Using estimated marginal mean from the statistical model, we tested the difference between the IIH and REF groups at the different points of follow-up.

The statistical analysis was performed using SPSS version 26 (IBM Corporation, Armonk, NY) and Stata/SE 15.0 (StataCrop LLC, College Station, TX). Statistical significance was accepted at the .05 level (two-tailed).

For Review Only

Results

Patients

Demographic and clinical data about the IIH (n=15) and REF (n=15) cohorts are presented in Table 1. They were comparable regarding gender and age. Visual obscurations and blurred vision were more common in IIH patients. The IIH patients fulfilled the diagnostic criteria of IIH disease.(Mollan *et al.*, 2018) Subjective cognitive disturbances were prevalent in both groups.

All the 10/15 IIH patients who underwent shunt surgery with implantation of a ventricular-peritoneal (VP) shunt responded favorably with improvement of visual function. Five of 15 IIH patients were recommended shunt surgery, but preferred conservative non-surgical treatment, and had lasting symptoms.

With regard to ICP scores, the pulsatile ICP, represented by the mean ICP wave amplitude (MWA) was significantly elevated in IIH subjects.

Prior to diagnostic assessment, 10/15 (67%) IIH subjects did not attend work/school, 3/15 (20%) partly attended work/school. Only 2/15 (13%) were attending work/school.

CSF tracer enrichment in CSF spaces of IIH and REF groups

Following intrathecal injection, the tracer rapidly enriched the CSF spaces (see Suppl. Video 1). The CSF space segmented by FreeSurfer was defined from the cavum veli interpositi (Suppl. Fig. 1), which is part of the subarachnoid compartment. Here, tracer enrichment was higher in IIH after 0.5-3 hours, but was comparable between groups at other time points (Fig. 1a). After 3-7 hours, there was similar tracer availability in CSF of both patient groups. Notably, tracer enrichment was significantly higher in 4th (Fig. 1b) and 3rd (Fig. 1c) ventricles of IIH at 3-7 hours, indicating flux of tracer towards the ventricular system early after injection. The tracer enrichment was not significant in lateral ventricles (Fig. 1d). Tracer enrichment in CSF spaces and choroid plexus is shown in Suppl. Table 1.

Brain-wide distribution of tracer in both IIH and REF groups

Following enrichment of the CSF spaces, the tracer spread throughout the entire brain, as illustrated in one IIH patient (Fig. 2A) and a REF subject (Fig. 2B). Suppl. Video 2 shows an animation of the tracer propagation within the brain tissue. The tracer enriched all regions of the

https://mc.manuscriptcentral.com/braincom

For Review Only

brain, including cortical gray matter, white matter, basal ganglia, and infra-tentorial regions, as further detailed in Suppl. Table 2.

Increased tracer enrichment and delayed clearance in the IIH group

In IIH patients, there was increased enhancement and reduced clearance of tracer from brain, most evidently in the frontal and temporal brain regions. In Fig. 3, the tracer enrichment from baseline at group level is shown for the IIH and REF cohorts, and presenting differences between groups at the lower panel. Notably, the increase in tracer level was most evident in frontal and temporal regions. Suppl. Fig. 1 provides a comparable illustration of differences in clearance of tracer between IIH and REF cohorts from evening Day 1 until morning Day 2.

As shown in Fig. 4, tracer enrichment was increased and clearance delayed in IIH patients within cerebral and cerebellar cortical gray and white matter, and within basal ganglia and brainstem. Observations particularly for limbic structures of IIH patients are illustrated in Fig. 5. A more comprehensive list of brain regions is provided in Suppl. Table 2.

After 4 weeks, tracer enrichment in brain and CSF was normalized and without any evidence of difference between the IIH and REF groups, see Suppl. Table 3.

Altered tracer enrichment in patients with pathological pulsatile ICP

Among the 22 of 30 study participants who had undergone overnight monitoring of ICP, overnight pulsatile ICP (MWA) was categorized as pathological in 14 (all IIH patients) and normal in 8 individuals (all REF subjects). Comparison of tracer propagation showed significantly increased tracer enrichment and delayed clearance from several brain regions in individuals with pathological MWA (Suppl. Table 4). This is further illustrated in Suppl. Fig. 3 for limbic brain structures, including amygdala, hippocampus, gray and white matter of parahippocampal cortex, gray and white matter of entorhinal cortex, gray and white matter of cingulate cortex, thalamus, accumbens area, and gray and white matter of insular cortex.

Discussion

The present results provide in vivo evidence for impaired glymphatic function in IIH. The brain-wide extra-vascular distribution of a CSF tracer was increased and clearance delayed in numerous brain regions of IIH patients as compared to age and gender matched REF subjects.

For Review Only

The REF cohort cannot be considered a material of healthy individuals, but was comparable with IIH patients regarding age and gender. They reported a high frequency of symptoms, even though no apparent CSF disturbance was verified, and therefore no intervention was advocated in these subjects.

The etiology of IIH has remained an enigma since the disease was described by the American neurosurgeon Walter E Dandy in 1937 (Dandy, 1937), first denoted pseudotumor cerebri (PTC). Here, we present for the first time in vivo evidence for impaired glymphatic function in a consecutive series of non-selected IIH subjects. The glymphatic system refers to a peri- (or para-) vascular transport route for convective transport of fluids and solutes consisting of an influx route for CSF along arteries and an efflux route along veins, after the free mixing of CSF with solutes within the interstitial space (Iliff *et al.*, 2012). The convective transport of fluids and solutes along vessels and the interstitial space is thought to be driven by forces created from the arterial pulsations (Mestre *et al.*, 2018). This transport route enables a pathway for clearance of byproducts from brain metabolism not being actively transported through the blood-brain–barrier (BBB). The glymphatic system is shown to be impaired with increasing age (Kress *et al.*, 2014) and in diseases, e.g. dementia disease and after traumatic brain injury (Reeves *et al.*, 2020), but has mostly been studied experimentally in rodents.

The CSF tracer used here is a highly hydrophilic and stable molecule of size 604 Da that does not pass the BBB or enter the cells. After its entry from CSF to brain, it is thus confined to the extra-vascular compartment, i.e. the perivascular and interstitial spaces. Due to the resolution of MRI of 1 mm, we cannot conclude about the relative distribution within the perivascular and interstitial spaces, and neither differentiate between the peri-arterial and peri-venous spaces. The speed by which gadobutrol enriches the entire brain is indicative of contribution from convective forces adding to diffusion, which also a previous modelling study indicated (Valnes *et al.*, 2020). Transport of gadobutrol predominated by diffusion is likely, at least in the interstitial space, as previously suggested (Holter *et al.*, 2017).

It is of particular interest that tracer enrichment and clearance of tracer from structures susceptible to neurodegeneration in dementia, including hippocampus and ERC, was delayed in IIH. It has previously been established that IIH subjects may suffer cognitive impairment (Kharkar *et al.*, 2011, Yri *et al.*, 2014). Among the present 15 IIH individuals, seven (47%) reported subjective cognitive impairment, primarily subjectively impaired short-term memory

For Review Only

and concentration. We previously found reduced postsynaptic density in IIH (Eide *et al.*, 2020), which is indicative of reduced synaptic strength in these patients. In dementia disease such as Alzheimer's, delayed clearance of toxic waste products such as amyloid-β and tau from CSF seems to be one mechanism behind neurodegeneration (Reeves *et al.*, 2020). It is therefore striking to note how distribution of areas with reduced molecular clearance in IIH match up with those most typically affected with amyloid-β and tau deposition in dementia diseases, including Alzheimer`s disease (Braak and Braak, 1991, Nedergaard and Goldman, 2020).

There was no evidence of signal change in any brain region after 4 weeks, which indicate complete clearance of gadobutrol from the brain. This finding is in line with previous observations in iNPH (Eide and Ringstad, 2019). We have previously reported that intrathecal gadobutrol in a dose of 0.5 mmol is safe (Edeklev *et al.*, 2019).

The group differences in parenchymal tracer enrichment may not be explained by differences in availability of tracer in CSF. We have previously shown that the enrichment of tracer in the brain heavily depends on availability of tracer within the CSF spaces (Ringstad *et al.*, 2017, Eide and Ringstad, 2019).

Given that increased CSF pressure is one diagnostic criterion for IIH (Ball and Clarke, 2006, Mollan *et al.*, 2018), it is of interest that tracer enrichment in CSF of cavum veli interpositi, 4th and 3rd ventricles differed from references the first few hours. The stronger ventricular enrichment of tracer in IIH compares with observations in iNPH (Ringstad *et al.*, 2017, Eide, 2020), and adds evidence to the association between reduced intracranial compliance (as shown by increased pulsatile ICP) and ventricular reflux. Here, it is hypothesized that both reduced compliance and CSF flow taking the path of least resistance (to ventricles) may have a common cause, i.e. increased resistance within CSF efflux pathways.

We hypothesize that the tracer moves from CSF spaces into the brain via the Virchow-Robin spaces and further via the periarterial spaces, i.e. within the basement membrane. The latter covers the endothelial cells and pericytes of capillary and is surrounded by the astrocytic endfeet, allowing passage of fluids and solutes to the interstitial space via the endfeet gaps, which are about 20 nm (Mathiisen *et al.*, 2010). The size of perivascular spaces seem to increase with increasing BMI (Barisano *et al.*, 2020), a hallmark of IIH (Mollan *et al.*, 2018). Moreover, a vascular involvement in IIH has previously been suggested, given that the prevalence of cardiovascular disease is increased in IIH (Fric *et al.*, 2017, Adderley *et al.*, 2019).

For Review Only

With regard to glymphatic failure, it should be noted that emerging evidence point at disturbance at the glia-neuro-vascular interface in IIH. From frontal cortical biopsies in IIH patients, we have shown pathological alterations in the basement membrane and pericytes of the cerebral capillaries (Eidsvaag *et al.*, 2018), and loss of BBB integrity and leakage of blood proteins to the perivascular space (Hasan-Olive *et al.*, 2019). Leakage of fibrinogen is pro-inflammatory, and a possible cause of the patchy astrogliosis characterizing the IIH brain (Eide *et al.*, 2016). Astrogliosis is a non-specific alteration in astrocytes with loss of domain, orientation, and evidence of swelling, which may be seen in many brain diseases (Verkhratsky and Nedergaard, 2018). A highly important anatomical feature of the brain vasculature is that the end-feet of the astrocytes completely ensheath the cerebral capillaries, which is required for maintenance of BBB function (Haddad-Tovolli *et al.*, 2017). Three-dimensional (3D) EM showed bundles of mitochondria in the end-foot processes close to perivascular end-foot membrane (Mathiisen *et al.*, 2010). In IIH, we found by TEM pathological mitochondria in astrocytic perivascular end-feet suggestive of altered cellular metabolism (Eide *et al.*, 2020). Shortened endoplasmic-reticular-contact sites also indicated cellular metabolic failure (Eide *et al.*, 2020). In IIH subjects, the astrocytic perivascular AQP4 expression was significantly increased in LM (Eide *et al.*, 2016) though immunogold TEM showed unaltered perivascular AQP4 (Hasan-Olive *et al.*, 2019). Higher demand on fluid exchange might result in a compensatory increase of perivascular AQP4. We suggest that in IIH alterations at the glia-neuro-vascular interface, particularly dysfunctional regulation of astrocytic endfeet that cover the capillaries, may affect perivascular solute transport and modify propagation of CSF tracer, as reported here.

We have previously reported that the pulsatile ICP is pathological in IIH, reflected by increased MWA scores, even in combination with normal static ICP (mean ICP) (Eide and Kerty, 2011, Fric and Eide, 2017). The present data further show that in patients with elevated pulsatile ICP, there was increased tracer influx and delayed tracer efflux from brain. The combination of impaired clearance of tracer and elevated pulsatile ICP compares with observations in iNPH (Eide, 2020).

Conclusions

The present study provides evidence for impaired glympahtic function in IIH patients, as revealed by increased influx and delayed clearance from parenchyma of an intrathecally administered CSF tracer. This was observed within numerous brain locations, but strikingly most in regions known susceptible to amyloid-β and tau deposition in Alzheimer`s disease, pointing to a possible mechanism behind cognitive dysfunction also in IIH. We suggest that in IIH pathological alterations at the glia-neuro-vascular interface may restrict transport of fluid and solutes along perivascular pathways and to and from interstitial tissue via astrocytic inter-endfeet gaps.

For Review Only

Declaration section

Acknowledgements

We thank Dr. Øivind Gjertsen, Dr. Bård Nedregaard and Dr. Ruth Sletteberg from the Department of Radiology, Oslo University Hospital – Rikshospitalet, who performed the intrathecal gadobutrol injections in all study subjects. We also sincerely thank the Intervention Centre and Department of Neurosurgery at Oslo University Hospital Rikshospitalet for providing valuable support with MR scanning and care taking of all study subjects throughout the study. Finally, we sincerely thank the Nurse Staff and Hydrocephalus outward clinic, Department of Neurosurgery Oslo University Hospital – Rikshospitalet for care taking of all study subjects throughout the study.

Funding

This work was supported by grants from Health South-East, Norway (grants 2020068), and from Department of neurosurgery, Oslo university hospital-Rikshospitalet, Oslo, Norway.

Competing interests

The authors disclose no conflicts of interest. GR has received a speaker fee from Bayer AG.

For Review Only

Figure legends

Fig. 1. Enrichment of tracer in intracranial CSF spaces over time in IIH and REF groups. Following lumbar intrathecal administration, the time course of tracer level is compared between IIH and REF groups within CSF spaces segmented using FreeSurfer, including (a) the subarachnoid space of pineal recess, (b) fourth ventricle, (c) third ventricle and (d) lateral ventricles. CSF tracer levels refers to percentage change of normalized T1 signal unit ratio, and trend plots for tracer level are shown as mean ± standard error (SE). Significant differences between groups are indicated by *P<0.05, **P<0.01, ***P<0.001 (mixed model analysis).

Fig. 2. Time course of parenchymal distribution of CSF tracer in an IIH patient and a REF subject. (A) gMRI from an IIH patient who complained of headache, visual obscurations, blurred vision, pulsatile tinnitus, olfactory dysfunction, dizziness, and subjective cognitive impairment. The pulsatile ICP was pathological (MWA 6.6 mmHg on average), while static ICP was within normal range (average of mean ICP 12.1 mmHg). (B) A REF patient at comparable age who complained of headache, dizziness and cognitive impairment. There was no sign of papilledema, and overnight ICP was normal. In both these patients, the CSF tracer (gadobutrol) was injected intrathecally, and MRI obtained after 3, 6, 24 and 48 hours. The images are post-processed using FreeSurfer software, and showing percentage increase in normalized T1 signal over time (see color scale to the right). Tracer in CSF spaces has been subtracted, thus only presenting tracer enrichment within brain tissue. After 3 and 6 hours, tracer enriches most strongly in brain regions adjacent to large artery trunks at the brain surface, including the medial temporal lobe close to the posterior cerebral arteries, the cingulum adjacent to the anterior cerebral arteries in the anterior interhemispheric fissure, and the Sylvian fissure, where the middle cerebral artery trunks reside. Notably, the tracer enriches the extra-vascular space of the brain from outside and inward (centripetally), with peak enhancement at about 24 hours.

Fig 3. Differences between the IIH and REF groups in enrichment of tracer within brain at 24 hours. The tracer in CSF spaces has been subtracted, showing tracer enrichment as percentage change in normalized T1 signal at 24 hours from baseline. For the IIH group (upper panel), the average percentage tracer enrichment from baseline to 24 hours is shown in sagittal (left), axial (middle), and coronal (right) plans. (For the REF group (middle panel), the

For Review Only

percentage tracer enrichment (normalized T1 signal) at 24 hours compared to baseline is shown in and sagittal (left), axial (middle) and coronal (right) plans, with color scale indicating percentage signal change. The difference in signal change between the cohorts (IIH minus REF groups) is shown in the lower panel. Red color represents areas with the highest tracer levels. From these images, tracer enrichment differed most and was highest in IIH in frontal, temporal, cingulate, insular, cerebellar and brainstem regions. In this patient group having some degree of cognitive impairment, it is notable that several of these areas correspond well with regions of tau-based neurofibrillary tangle progression in Alzheimer`s disease (Braak and Braak, 1991).

For Review Only

Fig. 4. Trend plots of tracer levels in different brain locations of the IIH and REF cohorts. The trend plots of percentage change in tracer enrichment (normalized signal unit ratio) reveal significantly higher tracer levels in enrichment and clearance phases in the IIH than the REF group. This was shown for (A) cerebral cortex gray matter, (B) cerebral white matter, (C) cerebellar cortex (gray matter), (D) cerebellar white matter, (E) basal ganglia and (F) brainstem. Trend plots are presented with mean ± standard error (SE). Statistical differences: $*P<0.05$, $**P<0.01$, $***P<0.001$ (linear mixed models).

Fig. 5. Increased CSF tracer levels in limbic structures of IIH patients indicative of reduced molecular clearance.

The trend plots of percentage change in tracer enrichment (normalized signal unit ratio) reveal significantly stronger tracer enrichment in enrichment and clearance phases in IIH as compared to REF groups for several brain regions belonging to the limbic system. The regions included (A) amygdala, (B) hippocampus, (C) parahippocampal cortex (gray matter), (D) parahippocampal white matter, (E) entorhinal cortex (gray matter), (F) entorhinal subcortex (white matter), (G) cingulate cortex (gray matter), (H) cingulate subcortex (white matter), (I) thalamus (J) accumbens area, (K) insular cortex (gray matter), and (L) insular subcortex (white matter). Trend plots are presented with mean ± standard error (SE). Statistical differences: $*P<0.05$, $**P<0.01$, $***P<0.001$ (linear mixed models).

References

Adderley NJ, Subramanian A, Nirantharakumar K, Yiangou A, Gokhale KM, Mollan SP, et al. Association Between Idiopathic Intracranial Hypertension and Risk of Cardiovascular Diseases in Women in the United Kingdom. JAMA neurology. 2019;76(9):1088-98.
Ball AK, Clarke CE. Idiopathic intracranial hypertension. Lancet neurology. 2006;5(5):433-42.
Barisano G, Sheikh-Bahaei N, Law M, Toga AW, Sepehrband F. Body mass index, time of day, and genetics affect perivascular spaces in the white matter. J Cereb Blood Flow Metab. 2020:271678x20972856.
Braak H, Braak E. Neuropathological stageing of Alzheimer-related changes. Acta Neuropathol. 1991;82(4):239-59.
Dandy WE. Intracranial Pressure without Brain Tumor: Diagnosis and Treatment. Ann Surg. 1937;106(4):492-513.
Edeklev CS, Halvorsen M, Lovland G, Vatnehol SAS, Gjertsen O, Nedregaard B, et al. Intrathecal Use of Gadobutrol for Glymphatic MR Imaging: Prospective Safety Study of 100 Patients. AJNR Am J Neuroradiol. 2019;40(8):1257-64.
Eide PK, Eidsvaag VA, Nagelhus EA, Hansson HA. Cortical astrogliosis and increased perivascular aquaporin-4 in idiopathic intracranial hypertension. Brain Res. 2016;1644:161-75.
Eide PK, Hasan-Olive MM, Hansson HA, Enger R. Increased occurrence of pathological mitochondria in astrocytic perivascular endfoot processes and neurons of idiopathic intracranial hypertension. J Neurosci Res. 2020.
Eide PK, Kerty E. Static and pulsatile intracranial pressure in idiopathic intracranial hypertension. Clin Neurol Neurosurg. 2011;113(2):123-8.
Eide PK, Pripp, A.H., Ringstad, G.A. Magnetic resonance imaging biomarkers of cerebrospinal fluid tracer dynamics in idiopathic normal pressure hydrocephalus. Brain Commun. 2020.
Eide PK, Ringstad G. Delayed clearance of cerebrospinal fluid tracer from entorhinal cortex in idiopathic normal pressure hydrocephalus: A glymphatic magnetic resonance imaging study. J Cereb Blood Flow Metab. 2019;39(7):1355-68.
Eide PK, Sorteberg W. Diagnostic intracranial pressure monitoring and surgical management in idiopathic normal pressure hydrocephalus: a 6-year review of 214 patients. Neurosurgery. 2010;66(1):80-91.
Eidsvaag VA, Hansson HA, Heuser K, Nagelhus EA, Eide PK. Cerebral microvascular abnormalities in patients with idiopathic intracranial hypertension. Brain Res. 2018;1686:72-82.
Fischl B. FreeSurfer. Neuroimage. 2012;62(2):774-81.
Fischl B, Salat DH, Busa E, Albert M, Dieterich M, Haselgrove C, et al. Whole brain segmentation: automated labeling of neuroanatomical structures in the human brain. Neuron. 2002;33(3):341-55.
Fric R, Eide PK. Comparative observational study on the clinical presentation, intracranial volume measurements, and intracranial pressure scores in patients with either Chiari malformation Type I or idiopathic intracranial hypertension. J Neurosurg. 2017;126(4):1312-22.
Fric R, Pripp AH, Eide PK. Cardiovascular risk factors in Chiari malformation and idiopathic intracranial hypertension. Brain Behav. 2017;7(5):e00677.
Friesner D, Rosenman R, Lobb BM, Tanne E. Idiopathic intracranial hypertension in the USA: the role of obesity in establishing prevalence and healthcare costs. Obesity reviews : an official journal of the International Association for the Study of Obesity. 2011;12(5):e372-80.

For Review Only

Haddad-Tovolli R, Dragano NRV, Ramalho AFS, Velloso LA. Development and Function of the Blood-Brain Barrier in the Context of Metabolic Control. Frontiers in neuroscience. 2017;11:224.
Hasan-Olive MM, Hansson HA, Enger R, Nagelhus EA, Eide PK. Blood-Brain Barrier Dysfunction in Idiopathic Intracranial Hypertension. J Neuropathol Exp Neurol. 2019;78(9):808-18.
Holter KE, Kehlet B, Devor A, Sejnowski TJ, Dale AM, Omholt SW, et al. Interstitial solute transport in 3D reconstructed neuropil occurs by diffusion rather than bulk flow. Proc Natl Acad Sci U S A. 2017;114(37):9894-9.
Iliff JJ, Wang M, Liao Y, Plogg BA, Peng W, Gundersen GA, et al. A paravascular pathway facilitates CSF flow through the brain parenchyma and the clearance of interstitial solutes, including amyloid beta. Science translational medicine. 2012;4(147):147ra11.
Kharkar S, Hernandez R, Batra S, Metellus P, Hillis A, Williams MA, et al. Cognitive impairment in patients with Pseudotumor Cerebri Syndrome. Behavioural neurology. 2011;24(2):143-8.
Kleinschmidt JJ, Digre KB, Hanover R. Idiopathic intracranial hypertension. Relationship To depression, anxiety, and quality of life. . Am J Ophthalmol. 2000;129(6):831.
Kress BT, Iliff JJ, Xia M, Wang M, Wei HS, Zeppenfeld D, et al. Impairment of paravascular clearance pathways in the aging brain. Ann Neurol. 2014;76(6):845-61.
Kunte H, Schmidt F, Kronenberg G, Hoffmann J, Schmidt C, Harms L, et al. Olfactory dysfunction in patients with idiopathic intracranial hypertension. Neurology. 2013;81(4):379-82.
Mathiisen TM, Lehre KP, Danbolt NC, Ottersen OP. The perivascular astroglial sheath provides a complete covering of the brain microvessels: an electron microscopic 3D reconstruction. Glia. 2010;58(9):1094-103.
Mestre H, Tithof J, Du T, Song W, Peng W, Sweeney AM, et al. Flow of cerebrospinal fluid is driven by arterial pulsations and is reduced in hypertension. Nature communications. 2018;9(1):4878.
Mollan SP, Aguiar M, Evison F, Frew E, Sinclair AJ. The expanding burden of idiopathic intracranial hypertension. Eye (London, England). 2019;33(3):478-85.
Mollan SP, Ali F, Hassan-Smith G, Botfield H, Friedman DI, Sinclair AJ. Evolving evidence in adult idiopathic intracranial hypertension: pathophysiology and management. J Neurol Neurosurg Psychiatry. 2016;87(9):982-92.
Mollan SP, Davies B, Silver NC, Shaw S, Mallucci CL, Wakerley BR, et al. Idiopathic intracranial hypertension: consensus guidelines on management. J Neurol Neurosurg Psychiatry. 2018;89(10):1088-100.
Nedergaard M, Goldman SA. Glymphatic failure as a final common pathway to dementia. Science. 2020;370(6512):50-6.
Reeves BC, Karimy JK, Kundishora AJ, Mestre H, Cerci HM, Matouk C, et al. Glymphatic System Impairment in Alzheimer's Disease and Idiopathic Normal Pressure Hydrocephalus. Trends Mol Med. 2020;26(3):285-95.
Reuter M, Schmansky NJ, Rosas HD, Fischl B. Within-subject template estimation for unbiased longitudinal image analysis. Neuroimage. 2012;61(4):1402-18.
Ringstad G, Valnes LM, Dale AM, Pripp AH, Vatnehol SS, Emblem KE, et al. Brain-wide glymphatic enhancement and clearance in humans assessed with MRI. JCI insight. 2018;3(13):1-16.
Ringstad G, Vatnehol SAS, Eide PK. Glymphatic MRI in idiopathic normal pressure hydrocephalus. Brain. 2017;140(10):2691-705.

For Review Only

Segonne F, Dale AM, Busa E, Glessner M, Salat D, Hahn HK, et al. A hybrid approach to the skull stripping problem in MRI. Neuroimage. 2004;22(3):1060-75.
Valnes LM, Mitusch SK, Ringstad G, Eide PK, Funke SW, Mardal KA. Apparent diffusion coefficient estimates based on 24 hours tracer movement support glymphatic transport in human cerebral cortex. Scientific reports. 2020;10(1):9176.
Verkhratsky A, Nedergaard M. Physiology of Astroglia. Physiol Rev. 2018;98(1):239-389.
Yri HM, Fagerlund B, Forchhammer HB, Jensen RH. Cognitive function in idiopathic intracranial hypertension: a prospective case-control study. BMJ open. 2014;4(4):e004376.

For Review Only

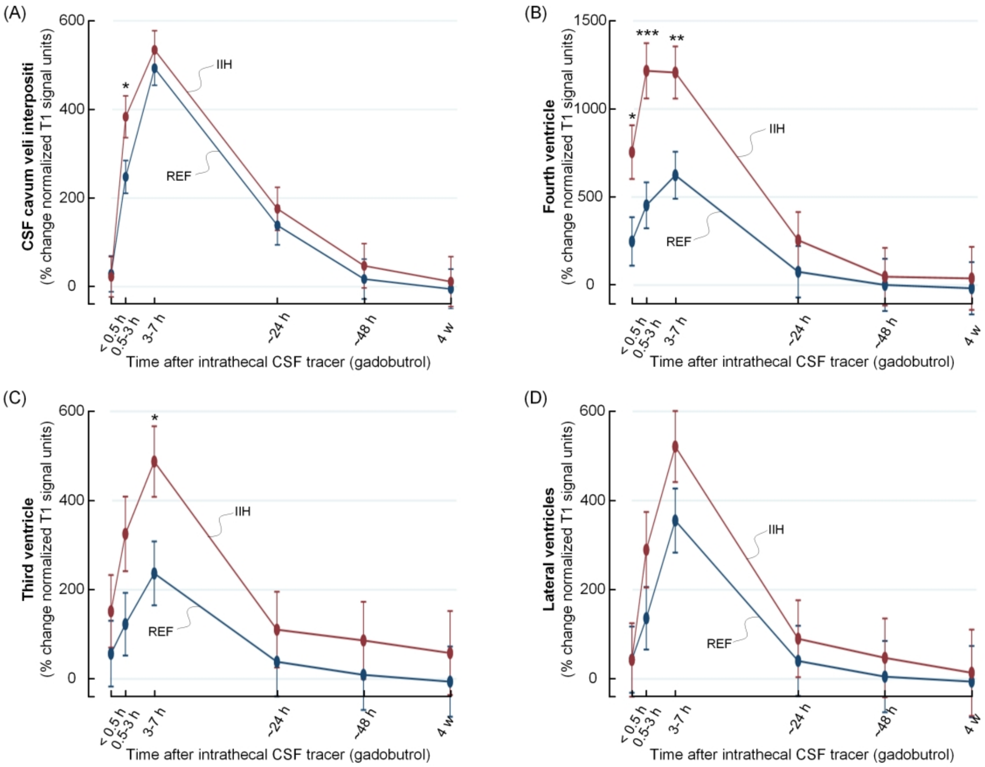

Fig 1

280x216mm (300 x 300 DPI)

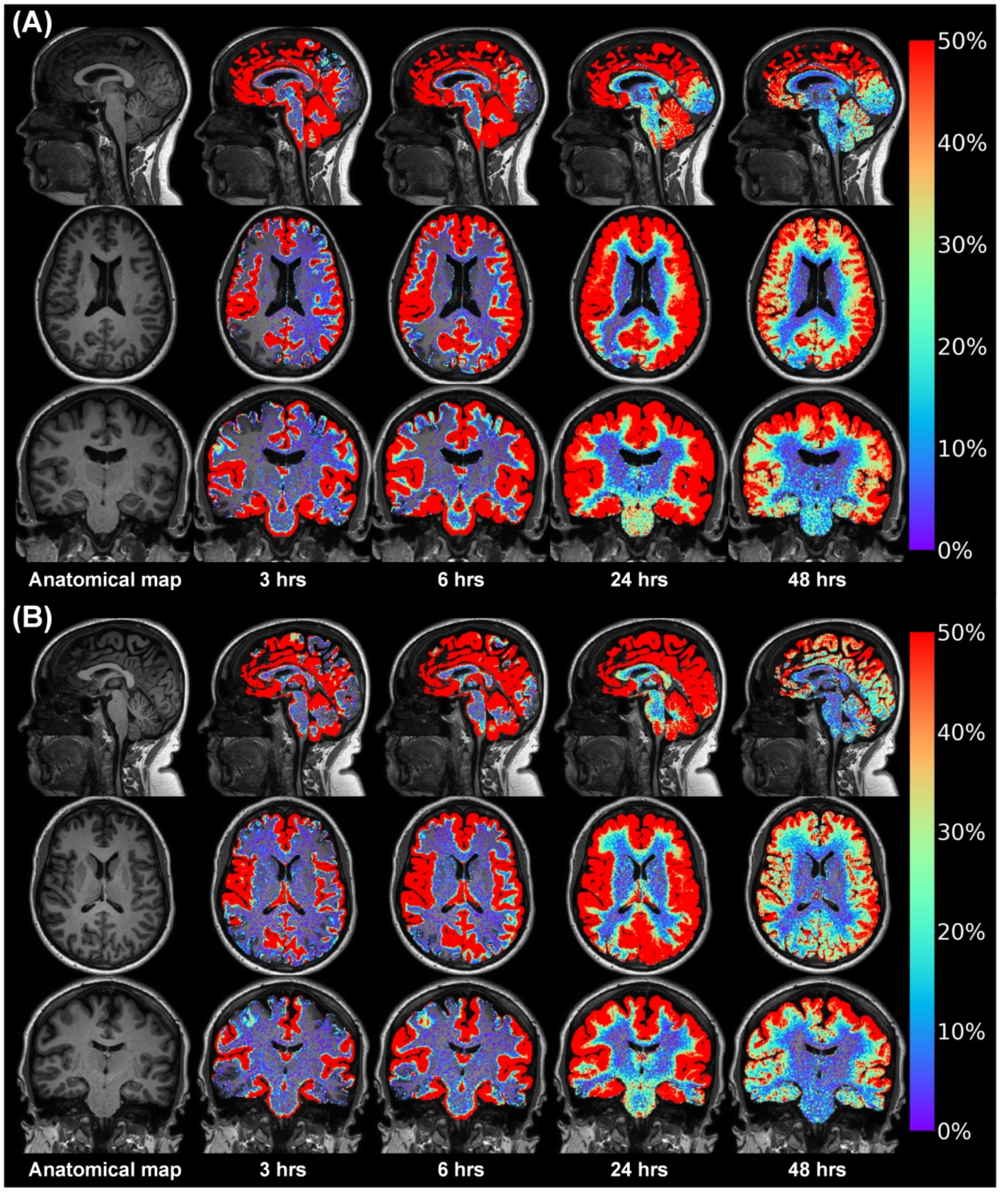

Fig 2

284x339mm (300 x 300 DPI)

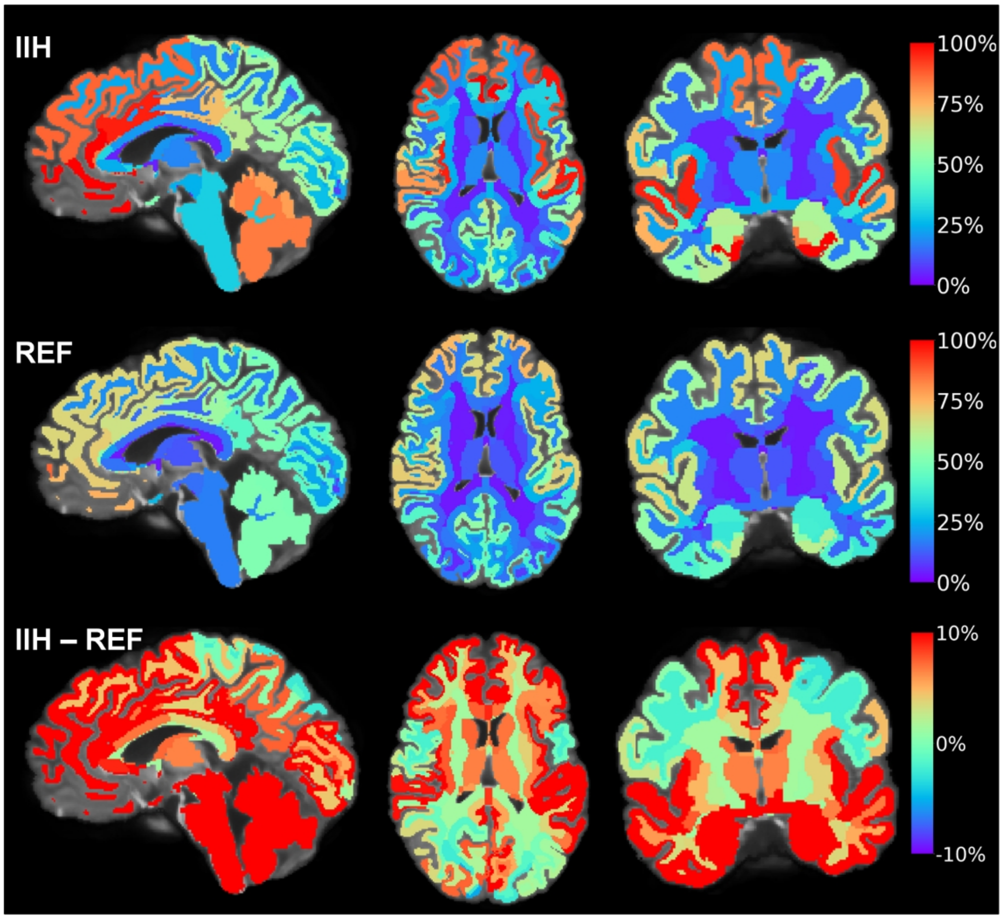

Fig 3

191x174mm (300 x 300 DPI)

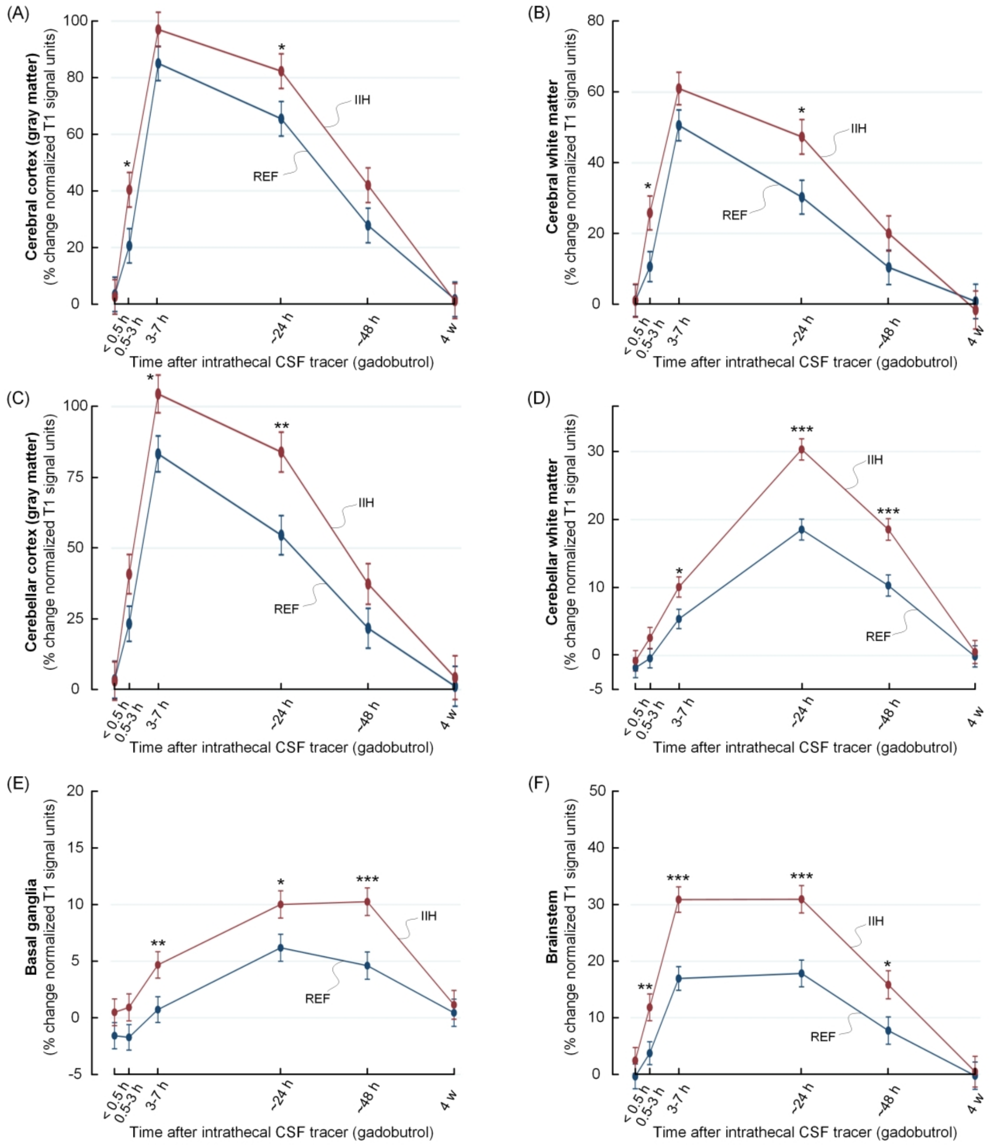

Fig 4

280x322mm (300 x 300 DPI)

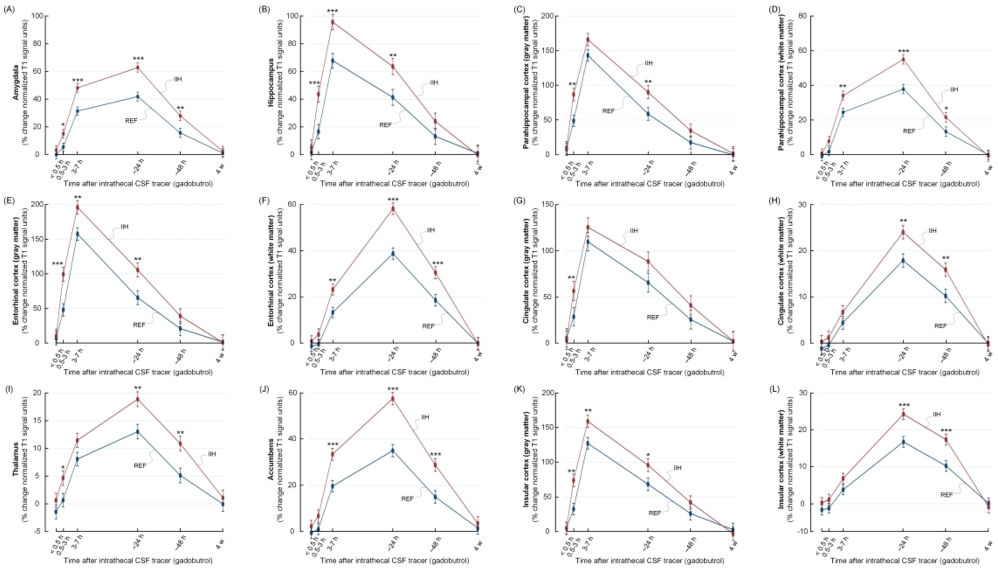

Fig 5

572x324mm (300 x 300 DPI)

TABLE 1. Demographic and clinical information about the IIH and REF cohorts.

	IIH (n=15)	REF (n=15)	P-value
Sex (F/M)	13/2	14/1	ns
Age (years)	35.6 ± 12.2	32.1 ± 6.5	ns
BMI (kg/m²)	31.1 ± 5.3	27.7 ± 6.2	ns
Symptoms reported by patient			
Headache	15 (100%)	15 (100%)	ns
Visual obscuration	8(53%)	2 (13%)	=0.02
Blurred vision	14 (93%)	4 (27%)	<0.001
Diplopia	6 (40%)	2 (13%)	ns
Pulsatile tinnitus	6 (40%)	2 (13%)	ns
Dizziness	11 (73%)	6 (40%)	ns
Cognitive disturbance	7 (47%)	10 (67%)	ns
Overnight ICP/MWA			
N	14	8	
Mean ICP, Average	11.6 ± 5.7	7.5 ± 3.3	ns
Mean ICP, % >15 mmHg	14.1 ± 13.8	3.4 ± 3.2	ns
MWA, Average	6.7 ± 1.8	3.7 ± 0.4	<0.001
MWA, % >5 mmHg	67.0 ± 20.0	8.8 ± 6.9	<0.001
IIH - Treatment and outcome			
VP shunt, clinical responder	10 (67%)		
Conservative without surgery	5 (33%)		

Categorical data presented as numbers (ranges in parentheses); continuous data presented as mean ± standard deviation or (minimum – maximum). Significant differences between groups were determined by Pearson Chi-square test for categorical data and by independent samples t-test for continuous data. IIH: Idiopathic intracranial hypertension. REF: Reference patients; ICP: intracranial pressure. MWA: mean ICP wave amplitude. Ns: non-significant differences between groups.

For Review Only

Table 2. Enrichment of tracer within the main brain regions.

Anatomical region	Group	0 – 0.5 hours				0.5 - 3 hours				3 - 7 hours				~24 hours				~48 hours			
		Mean	±	SE	p	Mean	±	SE	p	Mean	±	SE	p	Mean	±	SE	p	Mean	±	SE	p
Frontal cortex (GM)	IIH	2	±	7	ns	35	±	8	0.047	99	±	7	ns	97	±	8	0.033	50	±	8	ns
	REF	3	±	7		17	±	7		86	±	7		78	±	8		33	±	8	
Frontal cortex (WM)	IIH	0	±	2	ns	0	±	2	ns	8	±	2	ns	31	±	2	0.004	24	±	2	0.001
	REF	-1	±	2		-1	±	2		4	±	2		25	±	2		17	±	2	
Temporal cortex (GM)	IIH	3	±	8	ns	44	±	8	0.002	104	±	8	0.014	85	±	8	0.006	44	±	8	0.047
	REF	1	±	8		19	±	8		84	±	8		62	±	8		26	±	8	
Temporal cortex (WM)	IIH	0	±	2	ns	2	±	3	ns	12	±	2	0.031	35	±	2	<0.001	25	±	2	<0.001
	REF	-2	±	2		-2	±	2		7	±	2		27	±	2		16	±	2	
Parietal cortex (GM)	IIH	0	±	6	ns	12	±	6	ns	43	±	5	ns	59	±	6	ns	37	±	6	ns
	REF	2	±	5		10	±	5		54	±	5		62	±	6		29	±	6	
Parietal cortex (WM)	IIH	-1	±	2	ns	0	±	2	ns	3	±	1	ns	18	±	2	ns	17	±	2	ns
	REF	-1	±	1		-1	±	1		3	±	1		20	±	2		14	±	2	
Occipital cortex (GM)	IIH	0	±	6	ns	13	±	6	ns	40	±	6	ns	50	±	6	ns	30	±	6	ns
	REF	2	±	6		10	±	5		42	±	5		44	±	6		21	±	6	
Occipital cortex (WM)	IIH	-1	±	2	ns	1	±	2	ns	5	±	2	ns	20	±	2	ns	16	±	2	ns
	REF	-1	±	2		-1	±	2		4	±	2		18	±	2		12	±	2	
Cingulate cortex (GM)	IIH	3	±	10	ns	57	±	10	0.015	126	±	10	ns	89	±	10	0.054	41	±	10	ns
	REF	6	±	10		29	±	10		110	±	10		66	±	10		26	±	10	
Cingulate cortex (WM)	IIH	0	±	1	ns	1	±	1	ns	7	±	1	ns	24	±	1	0.001	16	±	1	0.002
	REF	-1	±	1		0	±	1		4	±	1		18	±	1		10	±	1	
Corpus callosum	IIH	1	±	2	ns	7	±	2	ns	20	±	2	ns	22	±	2	0.018	11	±	2	0.030
	REF	-1	±	2		3	±	2		16	±	2		15	±	2		5	±	2	

GM: Gray matter; WM: White matter. Data presented as percentage change in normalized T1 signal units over time. P: p-value (mixed model analysis); ns: non-significant differences between groups.

For Review Only

Supplementary Material

Impaired glymphatic function in idiopathic intracranial hypertension

Per Kristian Eide[1,2*], Are Hugo Pripp[3], Geir Ringstad[4], Lars Magnus Valnes[2]
[1]*Institute of Clinical Medicine, Faculty of Medicine, University of Oslo, Oslo, Norway.*
[2]*Department of Neurosurgery, Oslo University Hospital – Rikshospitalet, Oslo, Norway.*
[3]*Oslo Centre of Biostatistics and Epidemiology, Research Support Services, Oslo University Hospital, Oslo, Norway.*
[4]*Department of Radiology, Oslo University Hospital- Rikshospitalet, Oslo, Norway.*

Correspondence:

Professor Per Kristian Eide, MD PhD

Department of Neurosurgery

Oslo University Hospital - Rikshospitalet

Pb 4950 Nydalen,

N-0424 Oslo, Norway

p.k.eide@medisin.uio.no

For Review Only

Supplementary Video 1.
Animation of tracer enrichment over time within the intracranial CSF spaces in an IIH patient. Voxels in the CSF space displaying a higher T1 signal intensity than a given threshold were chosen for visualization.

Supplementary Video 2.
Animation of CSF tracer enrichment over time within the brain tissue in the same IIH patient presented in Suppl. Video 1. The same patient is also illustrated in Fig. 1A. The animation was generated by linear interpolation between the data points (images) with a time step of 30 minutes.

For Review Only

Supplementary Fig. 1

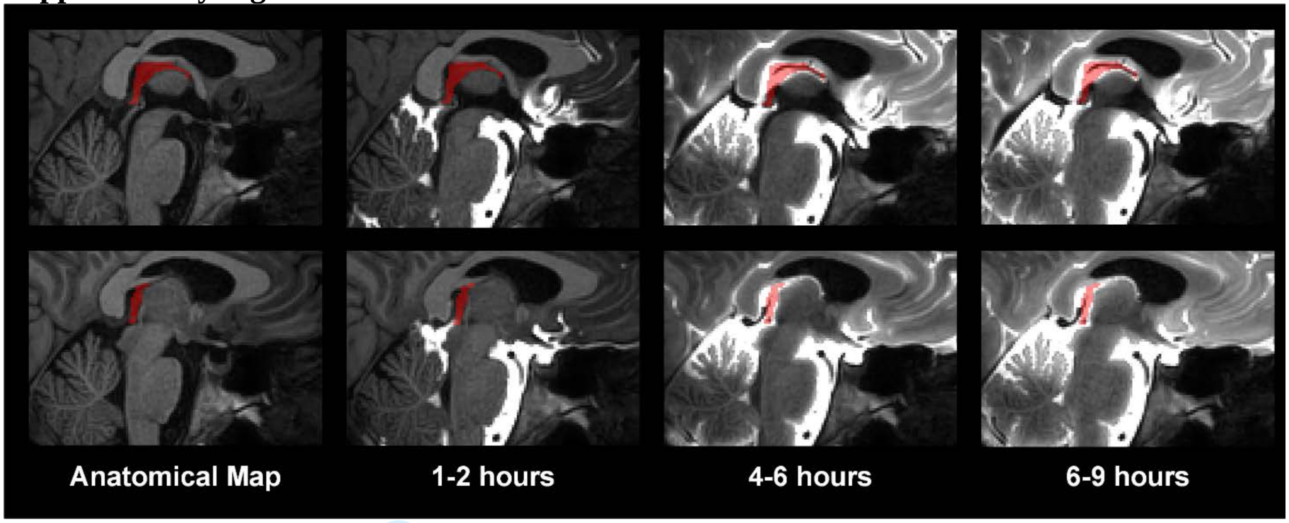

Automatic segmentation of CSF using FreeSurfer Software. The FreeSurfer Software segments CSF in the cavum veli interpositi, which is in free communication with the subarachnoid space. The images show tracer enrichment within subarachnoid spaces. The area segmented by FreeSurfer is marked with red color.

For Review Only

Supplementary Fig. 2.

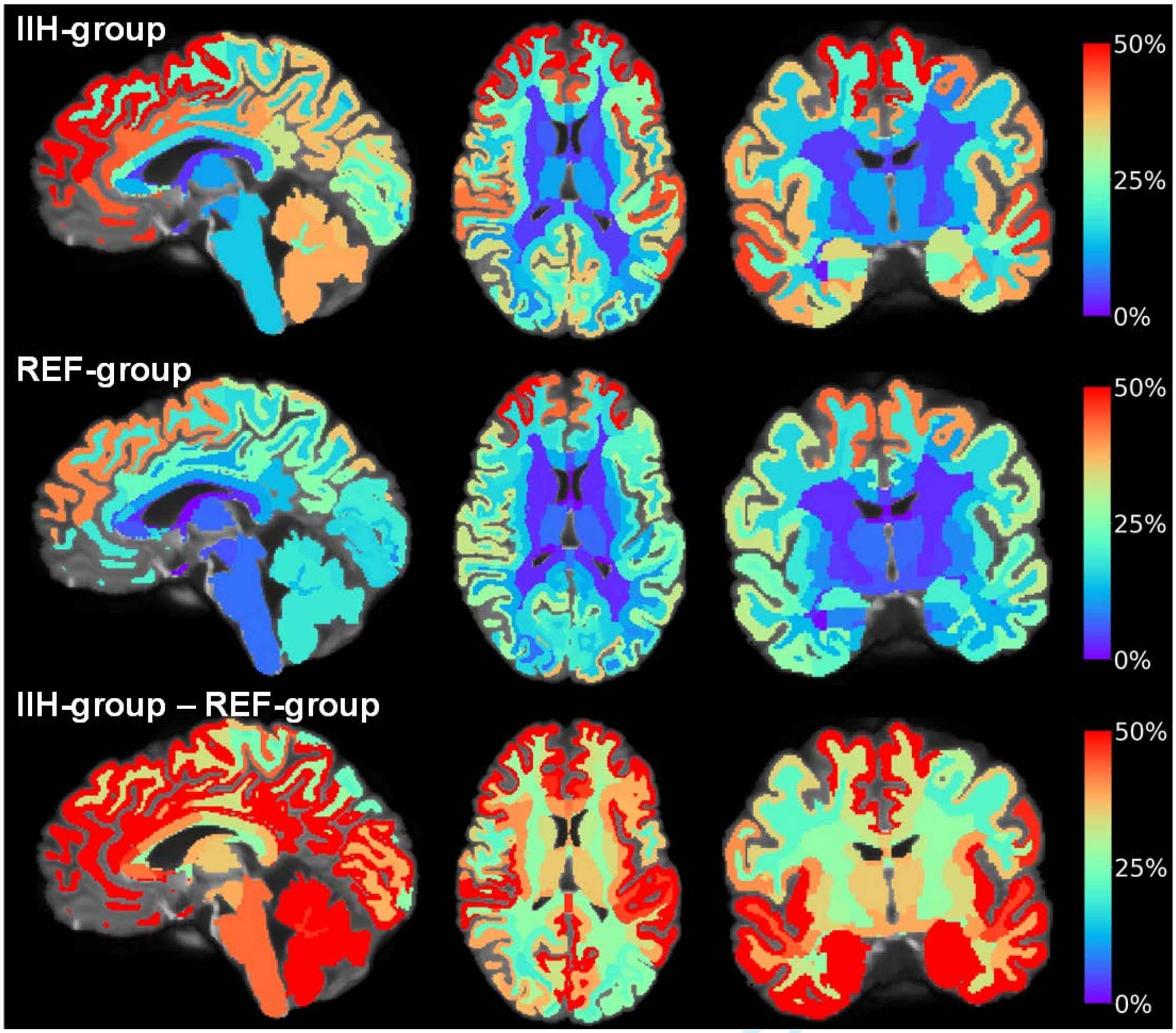

In vivo brain imaging demonstrates reduced molecular clearance in IIH from evening Day 1 until morning Day 2. Tracer enrichment in brain tissue at group level is expressed by percentage change of MRI T1 signal from evening Day 1 until morning Day 2, and shown on the color scale. The images show tracer enrichment within brain tissue, while tracer in CSF spaces have been subtracted. Upper panel: The tracer dependent signal change in brain is presented on average for the IIH group (n=15) from evening Day 1 to morning Day 2. The sagittal (left), axial (middle) and coronal (right) MRI scans are presented with the percentage signal increase indicated at the color scale. Middle panel: The average percentage signal change from tracer enrichment is presented for the REF group (n=15) from evening Day 1 to morning Day 2. Lower panel: The percentage difference in signal increase between the groups (IIH minus REF groups) is shown. It is shown by the color scale that tracer levels in brain tissue were higher in the IIH group compared to the REF group, i.e. clearance of tracer was least in the IIH group. Red color represents areas with the highest tracer enrichment. In particular, clearance of tracer in IIH was most impaired in frontal, temporal, cingulate cortical areas, close to the regions where the large arterial trunks (medial, anterior and posterior cerebral arteries) reside. Clearance also was reduced in cerebellum and brainstem.

Supplementary Fig. 3

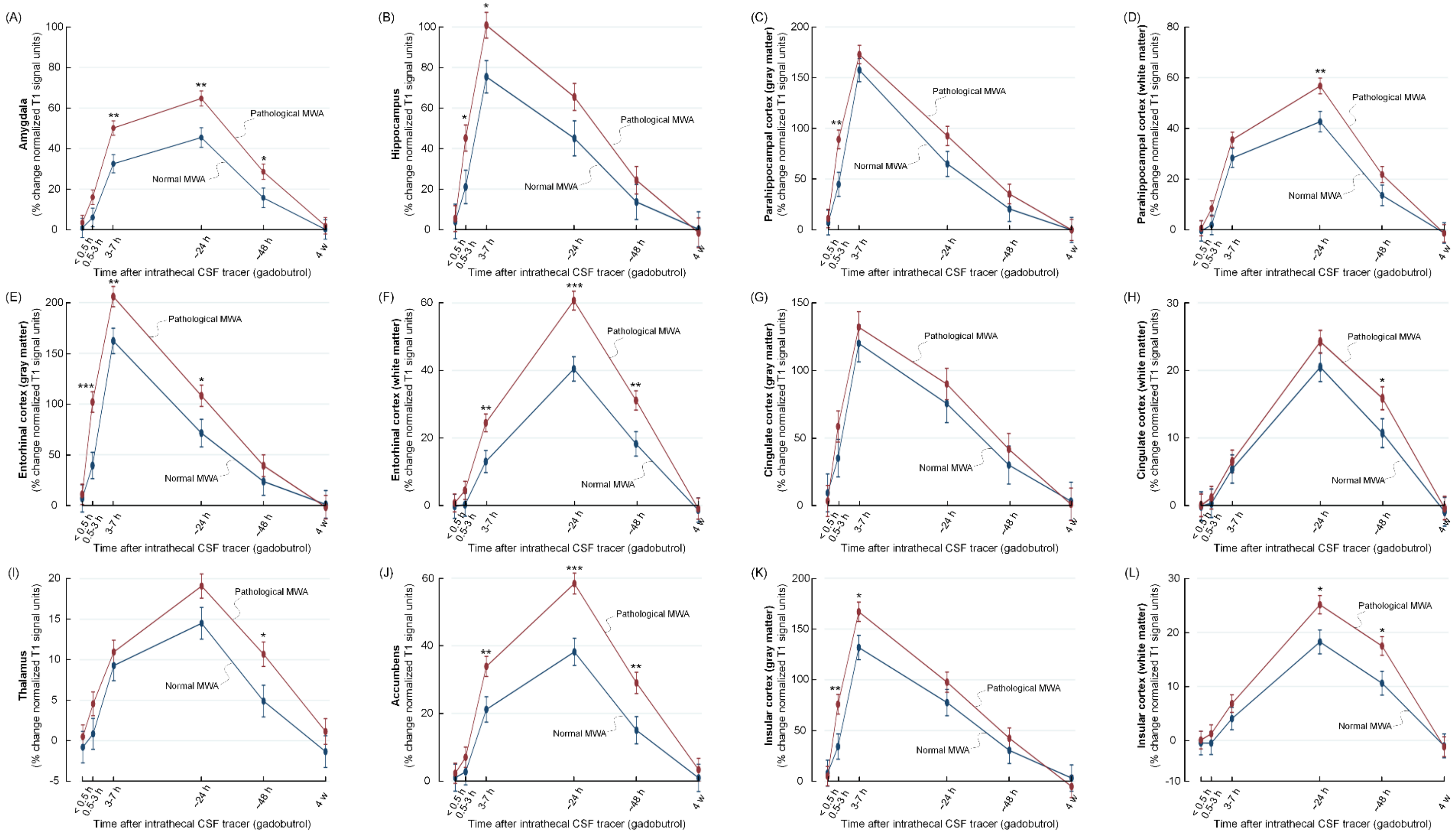

Increased CSF tracer levels in limbic structures of individuals with pathological pulsatile ICP. The trend plots of percentage change in tracer enrichment (normalized signal unit ratio) reveal significantly stronger tracer enrichment in enrichment and clearance phases of individuals with pathological pulsatile ICP (mean ICP wave amplitude; MWA; n=14) as compared to the individuals with normal MWA (n=8). The brain regions belonging to the limbic system included (A) amygdala, (B) hippocampus, (C) parahippocampal cortex (gray matter), (D) parahippocampal white matter, (E) entorhinal cortex (gray matter), (F) entorhinal cortex (white matter), (G) cingulate cortex (gray matter), (H) cingulate cortex (white matter), (I) thalamus (J) accumbens area, (K) insular cortex (gray matter), and (L) insular cortex (white matter). Trend plots are presented with mean ± standard error (SE). Statistical differences: *P<0.05, **P<0.01, ***P<0.001 (linear mixed models).

Supplementary Table 1. Enrichment of tracer within the CSF, cerebral ventricles and choroid plexus.

Anatomical region	Group	0 – 0.5 hours				0.5 - 3 hours				3 - 7 hours				~24 hours				~48 hours			
		**Mean**	**±**	**SE**	**p**	**Mean**	**±**	**SE**	**p**	**Mean**	**±**	**SE**	**p**	**Mean**	**±**	**SE**	**p**	**Mean**	**±**	**SE**	**p**
CSF	IIH	22	±	45	ns	383	±	47	=0.024	534	±	43	ns	176	±	49	ns	47	±	50	ns
	REF	29	±	40		248	±	37		493	±	38		138	±	44		17	±	45	
4th ventricle	IIH	755	±	153	=0.014	1217	±	157	<0.001	1207	±	148	=0.003	255	±	160	ns	48	±	164	ns
	REF	248	±	138		453	±	130		624	±	133		76	±	146		1	±	148	
3rd ventricle	IIH	152	±	82	ns	325	±	84	ns	488	±	79	=0.019	111	±	85	ns	86	±	87	ns
	REF	57	±	74		123	±	70		237	±	72		39	±	78		9	±	79	
Lateral ventricles	IIH	43	±	82	ns	290	±	85	ns	521	±	80	ns	90	±	86	ns	47	±	88	ns
	REF	43	±	74		136	±	70		355	±	72		40	±	79		5	±	80	
Choroid plexus	IIH	3	±	6	ns	26	±	6	ns	47	±	6	ns	23	±	6	ns	13	±	7	ns
	REF	4	±	6		18	±	6		49	±	6		16	±	6		8	±	6	

Data presented as percentage change in normalized T1 signal units over time. P: p-value (mixed model analysis); ns: non-significant differences between groups.

For Review Only

Supplementary Table 2. Comparison between IIH and REF groups of percentage change in tracer enrichment within brain at different time points.

		0 - 0.5 hours				0.5 - 3 hours				3 - 7 hours				~ 24 hours				~ 48 hours			
**Anatomical region**	**Group**	**Mean**	**±**	**SE**	**p**	**Mean**	**±**	**SE**	**p**	**Mean**	**±**	**SE**	**p**	**Mean**	**±**	**SE**	**p**	**Mean**	**±**	**SE**	**p**
Some main regions																					
Cerebral cortex (gray matter)	IIH	3	±	6	ns	40	±	6	0.013	97	±	6	ns	82	±	6	0.036	42	±	6	ns
	REF	3	±	6		21	±	6		85	±	6		66	±	6		28	±	6	
Cerebral white matter	IIH	1	±	5	ns	26	±	5	=0.018	61	±	5	ns	47	±	5	=0.013	20	±	5	ns
	REF	1	±	5		11	±	4		51	±	4		30	±	5		10	±	5	
Basal ganglia	IIH	0	±	1	ns	1	±	1	ns	5	±	1	=0.009	10	±	1	=0.016	10	±	1	<0.001
	REF	-2	±	1		-2	±	1		1	±	1		6	±	1		5	±	1	
Cerebellar cortex (gray matter)	IIH	3	±	7	ns	41	±	7	ns	105	±	7	0.021	84	±	7	=0.003	37	±	7	ns
	REF	4	±	7		23	±	6		83	±	6		55	±	7		22	±	7	
Cerebellar white matter	IIH	-1	±	2	ns	3	±	2	ns	10	±	2	=0.023	30	±	2	<0.001	19	±	2	<0.001
	REF	-2	±	2		0	±	1		5	±	1		19	±	2		10	±	2	
Brain Stem	IIH	2	±	2	ns	12	±	2	=0.009	31	±	2	<0.001	31	±	2	<0.001	16	±	3	0.019
	REF	0	±	2		4	±	2		17	±	2		18	±	2		8	±	2	
Limbic structures																					
Amygdala	IIH	3	±	3	ns	15	±	3	=0.026	48	±	3	<0.001	63	±	3	<0.001	28	±	3	=0.009
	REF	0	±	3		6	±	3		31	±	3		42	±	3		16	±	3	
Hippocampus	IIH	5	±	6	ns	44	±	6	<0.001	96	±	6	<0.001	64	±	6	0.006	24	±	6	ns
	REF	2	±	5		17	±	5		68	±	5		41	±	8		13	±	6	
Entorhinal cortex (gray matter)	IIH	10	±	10	ns	99	±	10	<0.001	196	±	10	=0..003	106	±	10	=0.005	39	±	10	ns
	REF	7	±	9		48	±	9		157	±	9		65	±	10		21	±	10	
Entorhinal cortex (white matter)	IIH	1	±	2	ns	4	±	3	ns	23	±	2	=0.002	58	±	3	<0.001	30	±	3	=0.001
	REF	-1	±	2		-1	±	2		13	±	2		39	±	3		19	±	3	

For Review Only

Parahippocampal cortex (gray matter)	IIH	10	±	9	ns	87	±	9	=0.002	166	±	9	ns	90	±	9	=0.016	35	±	10	ns
	REF	8	±	9		49	±	8		143	±	8		59	±	9		17	±	9	
Parahippocampal cortex (white matter)	IIH	1	±	3	ns	8	±	3	ns	34	±	3	=0.006	55	±	3	<0.001	22	±	3	0.033
	REF	-1	±	3		2	±	2		24	±	2		38	±	3		13	±	3	
Thalamus	IIH	1	±	1	ns	5	±	1	=0.024	12	±	1	ns	19	±	1	=0.002	11	±	1	=0.002
	REF	-1	±	1		1	±	1		8	±	1		13	±	1		5	±	1	
Cingulate cortex (gray matter)																					
Rostral anterior cingulate	IIH	6	±	13	ns	110	±	13	<0.001	212	±	13	=0.004	115	±	13	=0.032	48	±	14	ns
	REF	8	±	13		45	±	12		162	±	12		75	±	13		30	±	13	
Caudal anterior cingulate	IIH	3	±	11	ns	67	±	11	=0.021	155	±	11	=0.030	101	±	12	ns	45	±	12	ns
	REF	7	±	11		31	±	10		122	±	11		73	±	11		29	±	12	
Posterior cingulate	IIH	2	±	8	ns	24	±	8	ns	74	±	8	ns	75	±	8	ns	38	±	8	ns
	REF	4	±	8		16	±	7		75	±	7		65	±	8		27	±	8	
Isthmus cingulate	IIH	2	±	6	ns	25	±	6	ns	59	±	6	=0.016	63	±	7	ns	34	±	7	ns
	REF	3	±	6		20	±	6		80	±	6		50	±	6		19	±	7	
Insular cortex (gray matter)	IIH	5	±	9	ns	74	±	9	=0.001	159	±	9	=0.009	96	±	9	=0.034	42	±	9	ns
	REF	5	±	9		32	±	8		127	±	8		68	±	9		26	±	9	
Basal ganglia																					
Accumbens area	IIH	2	±	3	ns	7	±	3	ns	33	±	3	<0.001	58	±	3	<0.001	29	±	3	<0.001
	REF	-1	±	3		1	±	2		20	±	2		35	±	3		15	±	3	
Caudate nucleus	IIH	1	±	2	ns	3	±	2	ns	12	±	2	=0.001	11	±	2	=0.041	9	±	2	=0.021
	REF	-1	±	2		-1	±	2		3	±	2		5	±	2		3	±	2	
Putamen	IIH	1	±	1	ns	0	±	1	ns	1	±	1	ns	14	±	1	=0.012	15	±	1	<0.001
	REF	-2	±	1		-2	±	1		0	±	1		10	±	1		8	±	1	

For Review Only

Pallidum	IIH	0 ± 1	ns	0 ± 1	ns	1 ± 1	ns	6 ± 1	ns	7 ± 1	=0.005
	REF	-2 ± 1		-2 ± 1		-1 ± 1		4 ± 1		3 ± 1	
Frontal cortex (gray matter)											
Rostral middle frontal	IIH	-1 ± 6	ns	3 ± 6	ns	40 ± 6	ns	95 ± 6	ns	59 ± 7	=0.046
	REF	2 ± 6		4 ± 6		35 ± 6		85 ± 6		41 ± 6	
Caudal middle frontal	IIH	-2 ± 6	ns	1 ± 6	ns	25 ± 6	ns	57 ± 6	ns	46 ± 6	ns
	REF	1 ± 6		2 ± 5		30 ± 5		66 ± 6		35 ± 6	
Pars opercularis	IIH	0 ± 8	ns	33 ± 8	ns	102 ± 8	ns	92 ± 8	ns	48 ± 8	ns
	REF	2 ± 7		18 ± 7		93 ± 7		72 ± 8		31 ± 8	
Pars orbitalis	IIH	2 ± 8	ns	28 ± 8	ns	108 ± 8	ns	125 ± 8	=0.002	60 ± 9	=0.042
	REF	3 ± 8		14 ± 7		96 ± 7		88 ± 8		36 ± 8	
Pars triangularis	IIH	-1 ± 7	ns	26 ± 7	ns	98 ± 7	ns	100 ± 8	=0.024	52 ± 8	ns
	REF	1 ± 7		13 ± 6		86 ± 7		77 ± 7		32 ± 8	
Frontal pole	IIH	3 ± 12	ns	60 ± 12	=0.010	172 ± 12	=0.002	149 ± 13	=0.006	58 ± 13	ns
	REF	4 ± 11		18 ± 11		122 ± 11		101 ± 12		36 ± 13	
Superior frontal	IIH	0 ± 7	ns	14 ± 7	ns	57 ± 7	ns	94 ± 7	ns	56 ± 7	ns
	REF	2 ± 7		8 ± 7		45 ± 7		81 ± 7		40 ± 7	
Lateral orbito-frontal	IIH	6 ± 10	ns	76 ± 10	=0.002	178 ± 10	=0.003	117 ± 10	=0.003	51 ± 10	ns
	REF	6 ± 9		35 ± 9		140 ± 9		75 ± 10		29 ± 10	
Medial orbito-frontal	IIH	10 ± 13	ns	129 ± 14	<0.001	238 ± 13	=0.004	126 ± 14	=0.020	51 ± 14	ns
	REF	11 ± 13		57 ± 12		187 ± 13		81 ± 14		30 ± 14	
Precentral	IIH	-1 ± 6	ns	11 ± 7	ns	46 ± 6	ns	62 ± 7	ns	39 ± 7	ns
	REF	1 ± 6		9 ± 6		61 ± 6		67 ± 7		31 ± 7	
Paracentral	IIH	0 ± 6	ns	9 ± 6	ns	39 ± 6	ns	59 ± 6	ns	34 ± 6	ns
	REF	2 ± 6		9 ± 5		47 ± 6		64 ± 6		30 ± 6	

For Review Only

Temporal cortex (gray matter)											
Inferior temporal	IIH	0 ± 4	ns	5 ± 4	ns	27 ± 3	ns	57 ± 4	=0.006	44 ± 4	=0.001
	REF	-3 ± 3		-1 ± 3		20 ± 3		43 ± 4		26 ± 4	
Middle temporal	IIH	0 ± 6	ns	13 ± 6	ns	54 ± 5	ns	79 ± 6	=0.019	48 ± 6	=0.035
	REF	-3 ± 5		1 ± 5		42 ± 5		60 ± 6		31 ± 6	
Superior temporal	IIH	2 ± 9	ns	52 ± 9	=0.018	130 ± 9	ns	100 ± 9	=0.039	47 ± 9	ns
	REF	2 ± 8		23 ± 8		110 ± 8		74 ± 9		30 ± 9	
Temporal pole	IIH	9 ± 11	ns	95 ± 12	<0.001	206 ± 11	=0.004	137 ± 12	=0.003	56 ± 12	ns
	REF	7 ± 11		40 ± 10		162 ± 11		88 ± 12		30 ± 12	
Fusiform	IIH	1 ± 4	ns	18 ± 4	ns	57 ± 4	ns	63 ± 4	=0.017	35 ± 4	=0.034
	REF	0 ± 4		9 ± 4		46 ± 4		49 ± 4		22 ± 4	
Transverse temporal	IIH	3 ± 9	ns	80 ± 9	=0.001	147 ± 9	=0.043	76 ± 10	ns	34 ± 10	ns
	REF	3 ± 9		37 ± 8		123 ± 9		56 ± 9		21 ± 9	
Parietal cortex (gray matter)											
Postcentral	IIH	-1 ± 8	ns	19 ± 9	ns	65 ± 8	ns	74 ± 9	ns	43 ± 9	ns
	REF	1 ± 8		16 ± 8		87 ± 8		78 ± 9		33 ± 9	
Superior parietal	IIH	0 ± 5	ns	2 ± 6	ns	15 ± 5	ns	50 ± 6	ns	37 ± 6	ns
	REF	2 ± 5		4 ± 5		23 ± 5		63 ± 6		35 ± 6	
Inferior parietal	IIH	0 ± 5	ns	3 ± 5	ns	20 ± 5	ns	45 ± 5	ns	33 ± 5	ns
	REF	0 ± 5		1 ± 5		22 ± 5		50 ± 5		27 ± 5	
Supramarginal	IIH	0 ± 8	ns	21 ± 8	ns	68 ± 8	ns	77 ± 9	ns	43 ± 9	ns
	REF	1 ± 8		13 ± 7		81 ± 8		75 ± 8		33 ± 9	
Precuneus	IIH	1 ± 6	ns	11 ± 6	ns	37 ± 6	ns	56 ± 6	ns	38 ± 6	ns
	REF	3 ± 6		10 ± 5		49 ± 5		60 ± 6		27 ± 6	
Pericalcarine	IIH	1 ± 6	ns	17 ± 6	ns	52 ± 6	ns	55 ± 6	ns	31 ± 6	ns
	REF	2 ± 5		12 ± 5		59 ± 5		48 ± 6		21 ± 6	

For Review Only

Occipital cortex (gray matter)											
Cuneus	IIH	0 ± 6	ns	13 ± 6	ns	46 ± 6	ns	60 ± 6	ns	36 ± 6	ns
	REF	2 ± 6		14 ± 5		61 ± 6		56 ± 6		25 ± 6	
Lingual	IIH	1 ± 5	ns	25 ± 5	ns	67 ± 5	ns	64 ± 5	=0.051	31 ± 5	ns
	REF	3 ± 5		16 ± 5		61 ± 5		50 ± 5		20 ± 5	
Lateral occipital	IIH	-1 ± 3	ns	1 ± 3	ns	6 ± 3	ns	26 ± 3	ns	24 ± 3	ns
	REF	-1 ± 3		-1 ± 2		3 ± 3		26 ± 3		19 ± 3	
Corpus callosum											
Anterior	IIH	1 ± 2	ns	5 ± 2	ns	19 ± 2	ns	23 ± 2	=0.003	13 ± 2	=0.024
	REF	-1 ± 2		2 ± 2		16 ± 2		15 ± 2		6 ± 2	
Mid anterior	IIH	1 ± 3	ns	8 ± 3	ns	20 ± 3	ns	20 ± 3	ns	9 ± 3	ns
	REF	-1 ± 3		2 ± 2		14 ± 3		14 ± 3		4 ± 3	
Central	IIH	1 ± 3	ns	7 ± 3	ns	19 ± 3	ns	17 ± 3	ns	10 ± 3	ns
	REF	-1 ± 3		1 ± 3		12 ± 3		12 ± 3		4 ± 3	
Mid posterior	IIH	0 ± 3	ns	9 ± 3	ns	27 ± 3	ns	23 ± 3	ns	10 ± 3	ns
	REF	-1 ± 3		4 ± 3		25 ± 3		16 ± 3		4 ± 3	
Posterior	IIH	0 ± 2	ns	4 ± 2	ns	13 ± 2	ns	25 ± 2	=0.020	13 ± 2	ns
	REF	-1 ± 2		2 ± 2		15 ± 2		18 ± 2		7 ± 2	
Optic chiasm	IIH	23 ± 12	ns	142 ± 12	<0.001	187 ± 11	=0.001	63 ± 12	ns	19 ± 13	ns
	REF	16 ± 11		75 ± 10		137 ± 10		33 ± 12		4 ± 12	
Cingulate cortex (white matter)											
Rostral anterior cingulate	IIH	1 ± 2	ns	2 ± 2	ns	12 ± 2	=0.021	35 ± 2	<0.001	23 ± 2	<0.001
	REF	-1 ± 2		0 ± 2		7 ± 2		23 ± 2		14 ± 2	
Caudal anterior cingulate	IIH	0 ± 2	ns	1 ± 2	ns	7 ± 1	ns	23 ± 2	=0.002	15 ± 2	=0.008
	REF	-1 ± 1		0 ± 1		4 ± 1		16 ± 2		9 ± 2	

For Review Only

Posterior cingulate	IIH	0 ± 1	ns	1 ± 2	ns	4 ± 1	ns	18 ± 2	ns	12 ± 2	ns
	REF	-2 ± 1		-1 ± 1		3 ± 1		15 ± 2		9 ± 2	
Isthmus cingulate	IIH	0 ± 1	ns	1 ± 1	ns	4 ± 1	ns	20 ± 1	ns	14 ± 1	=0.017
	REF	-2 ± 1		-1 ± 1		4 ± 1		17 ± 1		9 ± 1	
Insular cortex (white matter)	IIH	0 ± 1	ns	1 ± 1	ns	7 ± 1	ns	24 ± 2	<0.001	17 ± 2	=0.001
	REF	-2 ± 1		-1 ± 1		4 ± 1		17 ± 1		10 ± 2	
Frontal cortex (white matter)											
Rostral middle frontal	IIH	-1 ± 2	ns	-1 ± 2	ns	1 ± 1	ns	19 ± 2	ns	22 ± 2	=0.012
	REF	-1 ± 1		-1 ± 1		-1 ± 1		17 ± 2		16 ± 2	
Caudal middle frontal	IIH	0 ± 2	ns	-1 ± 2	ns	0 ± 1	ns	10 ± 2	ns	15 ± 2	ns
	REF	-1 ± 1		-2 ± 1		0 ± 1		13 ± 2		12 ± 2	
Pars opercularis	IIH	0 ± 2	ns	1 ± 2	ns	6 ± 2	ns	27 ± 2	ns	23 ± 2	=0.004
	REF	-2 ± 2		-2 ± 2		4 ± 2		22 ± 2		16 ± 2	
Pars orbitalis	IIH	0 ± 2	ns	0 ± 2	ns	10 ± 2	ns	49 ± 2	<0.001	34 ± 2	<0.001
	REF	-1 ± 2		-1 ± 2		6 ± 2		38 ± 2		22 ± 2	
Pars triangularis	IIH	0 ± 2	ns	0 ± 2	ns	6 ± 2	ns	29 ± 2	ns	24 ± 2	=0.002
	REF	-1 ± 2		-1 ± 1		3 ± 2		24 ± 2		17 ± 2	
Frontal pole	IIH	-2 ± 3	ns	1 ± 3	ns	25 ± 3	=0.001	69 ± 3	<0.001	35 ± 3	=0.017
	REF	-1 ± 3		-2 ± 3		11 ± 3		50 ± 3		24 ± 3	
Superior frontal	IIH	0 ± 2	ns	0 ± 2	ns	2 ± 2	ns	24 ± 2	ns	24 ± 2	=0.018
	REF	-1 ± 2		-1 ± 2		0 ± 2		20 ± 2		18 ± 2	
Lateral orbitofrontal	IIH	1 ± 2	ns	2 ± 2	ns	11 ± 2	=0.016	38 ± 2	<0.001	28 ± 2	<0.001
	REF	-1 ± 2		-1 ± 1		6 ± 1		26 ± 2		17 ± 2	
Medial orbitofrontal	IIH	0 ± 2	ns	4 ± 2	ns	19 ± 2	=0.009	47 ± 2	<0.001	31 ± 2	<0.001
	REF	0 ± 2		1 ± 2		12 ± 2		32 ± 2		20 ± 2	
Precentral	IIH	0 ± 2	ns	-1 ± 2	ns	2 ± 2	ns	15 ± 2	ns	16 ± 2	ns
	REF	-2 ± 2		-2 ± 2		2 ± 2		19 ± 2		14 ± 2	

For Review Only

Paracentral	IIH	0 ± 2	ns	-1 ± 2	ns	1 ± 2	ns	16 ± 2	ns	16 ± 2	ns
	REF	-2 ± 2		-2 ± 1		0 ± 2		17 ± 2		14 ± 2	
Temporal cortex (white matter)											
Inferior temporal	IIH	-1 ± 1	ns	-1 ± 2	ns	2 ± 1	ns	17 ± 2	ns	21 ± 2	<0.001
	REF	-3 ± 1		-3 ± 1		0 ± 1		14 ± 2		13 ± 2	
Middle temporal	IIH	-1 ± 2	ns	0 ± 2	ns	3 ± 2	ns	24 ± 2	=0.032	23 ± 2	=0.001
	REF	-3 ± 2		-3 ± 2		1 ± 2		18 ± 2		15 ± 2	
Superior temporal	IIH	0 ± 2	ns	2 ± 2	ns	13 ± 2	ns	37 ± 2	=0.005	25 ± 2	=0.010
	REF	-2 ± 2		-2 ± 2		10 ± 2		29 ± 2		17 ± 2	
Temporal pole	IIH	1 ± 3	ns	4 ± 3	ns	29 ± 3	=0.016	79 ± 3	<0.001	45 ± 3	<0.001
	REF	0 ± 3		0 ± 3		19 ± 3		58 ± 3		29 ± 3	
Fusiform	IIH	-1 ± 1	ns	0 ± 1	ns	3 ± 1	ns	17 ± 1	ns	16 ± 1	=0.003
	REF	-2 ± 1		-2 ± 1		1 ± 1		14 ± 1		10 ± 1	
Transverse temporal	IIH	0 ± 2	ns	4 ± 3	ns	23 ± 2	=0.003	38 ± 3	=0.002	22 ± 3	=0.047
	REF	-2 ± 2		-1 ± 2		14 ± 2		27 ± 3		14 ± 3	
Parietal cortex (white matter)											
Postcentral	IIH	-1 ± 2	ns	1 ± 2	ns	7 ± 2	ns	27 ± 2	ns	20 ± 3	ns
	REF	-2 ± 2		-2 ± 2		9 ± 2		30 ± 2		18 ± 2	
Superior parietal	IIH	-1 ± 2	ns	-1 ± 2	ns	0 ± 2	ns	12 ± 2	ns	15 ± 2	ns
	REF	-1 ± 2		-1 ± 1		0 ± 1		17 ± 2		14 ± 2	
Inferior parietal	IIH	0 ± 2	ns	0 ± 2	ns	0 ± 2	ns	10 ± 2	ns	13 ± 2	ns
	REF	-2 ± 2		-2 ± 1		-1 ± 1		12 ± 2		11 ± 2	
Supramarginal	IIH	-1 ± 2	ns	0 ± 2	ns	3 ± 2	ns	21 ± 2	ns	19 ± 2	ns
	REF	-2 ± 2		-2 ± 2		3 ± 2		21 ± 2		16 ± 2	
Precuneus	IIH	-1 ± 1	ns	0 ± 1	ns	2 ± 1	ns	16 ± 2	ns	15 ± 2	ns
	REF	-1 ± 1		-1 ± 1		2 ± 1		19 ± 1		13 ± 2	
Pericalcarine	IIH	-1 ± 2	ns	1 ± 2	ns	5 ± 2	ns	21 ± 2	ns	17 ± 2	=0.050
	REF	-1 ± 2		-1 ± 2		5 ± 2		19 ± 2		12 ± 2	

For Review Only

Occipital cortex (white matter)																					
Cuneus	IIH	-1	±	2	ns	1	±	2	ns	6	±	2	ns	26	±	2	ns	19	±	2	ns
	REF	-1	±	2		-1	±	2		7	±	2		24	±	2		15	±	2	
Lingual	IIH	-1	±	2	ns	2	±	2	ns	9	±	2	ns	26	±	2	ns	16	±	2	=0.024
	REF	-1	±	2		0	±	2		8	±	2		22	±	2		11	±	2	
Lateral occipital	IIH	-1	±	1	ns	0	±	1	ns	-2	±	1	ns	9	±	1	ns	12	±	1	ns
	REF	-1	±	1		-2	±	1		-3	±	1		8	±	1		9	±	1	

Ns: non-significant differences between groups.

For Review Only

Supplementary Table 3. Comparisons between IIH and REF groups of percentage change in tracer within brain tissue after 4 weeks.

	IIH			REF			Difference (IIH – REF)			
**Anatomical region**	**Mean**	**±**	**SE**	**Mean**	**±**	**SE**	**Mean**	**±**	**SE**	**p**
Some main regions of brain parenchyma										
Cerebral cortex (gray matter)	1.1	±	6.2	1.7	±	6.1	-0.6	±	8.1	0.941
Cerebral white matter	-1.7	±	5.4	0.8	±	4.9	-2.4	±	7.3	0.739
Basal ganglia	1.1	±	1.3	0.4	±	1.2	0.7	±	1.7	0.671
Cerebellar cortex (gray matter)	4.3	±	7.7	1.2	±	7.0	3.1	±	10.5	0.769
Cerebellar white matter	0.5	±	1.7	-0.1	±	1.6	0.7	±	2.3	0.779
Brain Stem	0.4	±	2.7	-0.3	±	2.4	0.7	±	3.6	0.846
Limbic structures										
Amygdala	2.4	±	3.6	1.1	±	3.2	1.3	±	4.8	0.791
Hippocampus	-0.2	±	6.4	1.0	±	5.8	-1.2	±	8.6	0.887
Entorhinal cortex (gray matter)	0.6	±	11.2	1.3	±	10.2	-0.7	±	15.1	0.965
Entorhinal cortex (white matter)	-0.4	±	2.8	-0.2	±	2.5	-0.2	±	3.8	0.954
Parahippocampal cortex (gray matter)	1.5	±	10.1	-0.4	±	9.3	1.8	±	13.7	0.893
Parahippocampal cortex (white matter)	-0.8	±	3.0	-0.1	±	2.7	-0.8	±	4.1	0.853
Thalamus	1.1	±	1.4	0	±	1.3	1.1	±	1.9	0.567
Cingulate cortex (gray matter)										
Rostral anterior cingulate	1.2	±	14.6	2.9	±	13.4	-1.8	±	19.8	0.928
Caudal anterior cingulate	1.1	±	12.6	2.9	±	11.5	-1.8	±	17.0	0.917
Posterior cingulate	1.4	±	8.8	2.3	±	8.1	-0.8	±	11.9	0.945
Isthmus cingulate	5.9	±	7.1	1.0	±	6.5	4.9	±	9.6	0.612
Insular cortex (gray matter)	-2.8	±	10.1	2.7	±	9.2	-5.4	±	13.7	0.691
Basal ganglia										
Accumbens area	3.5	±	2.9	1.5	±	2.7	2.0	±	4.0	0.612

For Review Only

Caudate nucleus	2.5	±	2.2	0.7	±	2.1	1.8	±	3.1	0.549
Putamen	1.2	±	1.2	1.0	±	1.1	0.3	±	1.6	0.862
Pallidum	-0.2	±	1.0	-0.2	±	0.9	0.0	±	1.4	0.993
Frontal cortex (gray matter)										
Rostral middle frontal	4.7	±	7.2	3.3	±	6.4	1.4	±	9.6	0.886
Caudal middle frontal	0.5	±	6.9	2.4	±	6.2	-2.0	±	9.2	0.831
Pars opercularis	-2.5	±	8.9	1.9	±	8.0	-4.4	±	12.0	0.714
Pars orbitalis	3.0	±	9.4	2.1	±	8.4	0.9	±	12.6	0.942
Pars triangularis	-0.8	±	8.4	2.3	±	7.5	-3.1	±	11.2	0.785
Frontal pole	8.3	±	13.9	3.8	±	12.5	4.4	±	18.9	0.812
Superior frontal	3.7	±	7.9	3.4	±	7.2	0.3	±	10.7	0.979
Lateral orbito-frontal	1.5	±	11.2	1.9	±	10.1	-0.4	±	15.1	0.978
Medial orbito-frontal	2.8	±	15.2	1.6	±	13.9	1.2	±	20.6	0.953
Precentral	-2.3	±	7.4	1.8	±	6.6	-4.1	±	9.9	0.679
Paracentral	0.4	±	6.7	2.1	±	6.1	-1.7	±	9.1	0.854
Temporal cortex (gray matter)										
Inferior temporal	2.7	±	4.1	0.5	±	3.7	2.2	±	5.5	0.689
Middle temporal	0.0	±	6.3	0.8	±	5.7	-0.8	±	8.6	0.930
Superior temporal	-2.7	±	10.0	1.5	±	9.1	-4.2	±	13.5	0.755
Temporal pole	4.6	±	13.1	3.4	±	11.9	1.1	±	17.6	0.949
Fusiform	2.4	±	4.8	0.1	±	4.4	2.3	±	6.5	0.722
Transverse temporal	-3.5	±	10.4	1.4	±	9.4	-4.9	±	14.0	0.728
Parietal cortex (gray matter)										
Postcentral	-4.9	±	9.7	1.4	±	8.7	-6.3	±	13.0	0.628
Superior parietal	1.4	±	6.2	3.2	±	5.6	-1.7	±	8.4	0.836
Inferior parietal	0.3	±	5.8	1.6	±	5.2	-1.3	±	7.8	0.865
Supramarginal	-5.6	±	9.3	0.9	±	8.5	-6.5	±	12.6	0.605
Precuneus	5.7	±	6.5	1.8	±	6.0	3.9	±	8.8	0.660
Pericalcarine	8.8	±	6.4	1.4	±	5.8	7.4	±	8.7	0.392

For Review Only

Occipital cortex (gray matter)										
Cuneus	6.4	±	6.8	1.6	±	6.2	4.8	±	9.2	0.601
Lingual	4.8	±	5.8	0.8	±	5.2	4.0	±	7.8	0.605
Lateral occipital	4.8	±	3.0	1.4	±	2.8	3.4	±	4.1	0.405
Corpus callosum										
Anterior	-1.3	±	2.2	0.2	±	2.0	-1.5	±	2.9	0.610
Mid anterior	-0.1	±	3.1	0.1	±	2.8	-0.2	±	4.2	0.958
Central	0.3	±	3.2	-0.4	±	2.9	0.8	±	4.3	0.861
Mid posterior	0.0	±	3.7	-0.6	±	3.3	0.6	±	5.0	0.899
Posterior	-0.5	±	2.3	-0.4	±	2.1	-0.1	±	3.1	0.975
Optic chiasm	-4.2	±	13.9	-2.1	±	12.3	-2.1	±	18.5	0.913
Cingulate cortex (white matter)										
Rostral anterior cingulate	-0.2	±	1.8	0.1	±	1.7	-0.4	±	2.5	0.881
Caudal anterior cingulate	-0.4	±	1.6	0.4	±	1.5	-0.7	±	2.2	0.743
Posterior cingulate	-0.4	±	1.6	-0.2	±	1.5	-0.2	±	2.2	0.915
Isthmus cingulate	-0.3	±	1.5	-0.4	±	1.4	0.1	±	2.0	0.944
Insular cortex (white matter)	-0.9	±	1.6	0.1	±	1.5	-0.9	±	2.2	0.667
Frontal cortex (white matter)										
Rostral middle frontal	0.2	±	1.7	0.3	±	1.5	-0.1	±	2.3	0.969
Caudal middle frontal	-0.7	±	1.7	0.3	±	1.5	-1.0	±	2.3	0.651
Pars opercularis	-1.5	±	2.0	0.3	±	1.8	-1.7	±	2.7	0.520
Pars orbitalis	0.4	±	2.6	0.1	±	2.3	0.4	±	3.4	0.919
Pars triangularis	-1.3	±	1.9	0.2	±	1.7	-1.4	±	2.5	0.569
Frontal pole	2.6	±	3.6	0.8	±	3.2	1.7	±	4.8	0.720
Superior frontal	0.4	±	2.0	0.5	±	1.8	-0.1	±	2.7	0.982
Lateral orbitofrontal	0.0	±	1.8	0.0	±	1.6	0.0	±	2.4	0.992

For Review Only

Medial orbitofrontal	0.7	±	2.2	0	±	2.0	0.7	±	3.0	0.811
Precentral	-1.3	±	1.9	0.3	±	1.7	-1.6	±	2.6	0.524
Paracentral	-0.4	±	1.8	0.2	±	1.6	-0.7	±	2.4	0.787
Temporal cortex (white matter)										
Inferior temporal	-0.8	±	1.6	-0.3	±	1.5	-0.5	±	2.2	0.827
Middle temporal	-0.9	±	1.9	-0.3	±	1.7	-0.6	±	2.6	0.819
Superior temporal	-1.6	±	2.3	0.1	±	2.1	-1.6	±	3.1	0.602
Temporal pole	0.5	±	3.5	0.5	±	3.1	0.0	±	4.6	0.997
Fusiform	-0.5	±	1.4	-0.5	±	1.3	0.1	±	1.9	0.979
Transverse temporal	-1.6	±	2.7	0.2	±	2.5	-1.8	±	3.7	0.623
Parietal cortex (white matter)										
Postcentral	-2.1	±	2.7	0.1	±	2.4	-2.2	±	3.6	0.540
Superior parietal	0	±	1.8	0.3	±	1.6	-0.3	±	2.4	0.892
Inferior parietal	-0.4	±	1.7	-0.1	±	1.6	-0.3	±	2.3	0.912
Supramarginal	-1.7	±	2.1	-0.3	±	1.9	-1.4	±	2.8	0.620
Precuneus	0.6	±	1.6	0	±	1.5	0.6	±	2.1	0.788
Pericalcarine	2.2	±	1.9	0.1	±	1.7	2.2	±	2.6	0.389
Occipital cortex (white matter)										
Cuneus	2.0	±	2.2	0.4	±	2.0	1.7	±	3.0	0.576
Lingual	0.8	±	1.8	-0.2	±	1.6	1.0	±	2.4	0.689
Lateral occipital	1.5	±	1.5	0.3	±	1.4	1.3	±	2.1	0.541

P: p-value; ns: non-significant differences between groups.

For Review Only

Supplementary Table 4. CSF tracer levels depending on pulsatile ICP (MWA levels) in some brain regions.

		0 - 0.5 hours				0.5 - 3 hours				3 - 7 hours				~ 24 hours				~ 48 hours			
**Anatomical region**	**MWA-Group**	**Mean**	**±**	**SE**	**p**	**Mean**	**±**	**SE**	**p**	**Mean**	**±**	**SE**	**p**	**Mean**	**±**	**SE**	**p**	**Mean**	**±**	**SE**	**p**
Some main regions																					
Frontal cortex (gray matter)	Pathological	1	±	8	ns	36	±	8	ns	104	±	8	ns	98	±	8	ns	50	±	8	ns
	Normal	7	±	10		21	±	10		88	±	10		91	±	10		39	±	10	
Frontal cortex (white matter)	Pathological	-1	±	2	ns	1	±	2	ns	8	±	2	ns	32	±	2	ns	25	±	2	=0.029
	Normal	0	±	2		0	±	2		4	±	2		28	±	2		18	±	2	
Temporal cortex (gray matter)	Pathological	3	±	9	ns	46	±	9	=0.011	109	±	9	=0.015	87	±	9	ns	44	±	9	ns
	Normal	3	±	11		18	±	11		83	±	11		67	±	11		29	±	11	
Temporal cortex (white matter)	Pathological	0	±	3	ns	2	±	3	ns	13	±	3	ns	36	±	3	=0.009	25	±	3	=0.008
	Normal	-1	±	3		-2	±	3		7	±	3		28	±	3		17	±	3	
Parietal cortex (gray matter)	Pathological	0	±	6	ns	13	±	6	ns	45	±	6	ns	59	±	6	ns	37	±	6	ns
	Normal	6	±	8		12	±	8		54	±	8		69	±	8		32	±	8	
Parietal cortex (white matter)	Pathological	-1	±	2	ns	0	±	2	ns	3	±	2	ns	18	±	2	ns	16	±	2	ns
	Normal	0	±	2		-1	±	2		3	±	2		22	±	2		14	±	2	
Occipital cortex (gray matter)	Pathological	1	±	6	ns	14	±	6	ns	42	±	6	ns	51	±	6	ns	31	±	6	ns
	Normal	3	±	7		10	±	7		44	±	7		48	±	7		21	±	7	
Occipital cortex (white matter)	Pathological	-1	±	2	ns	1	±	2	ns	5	±	2	ns	20	±	2	ns	16	±	2	ns
	Normal	-1	±	2		-1	±	2		4	±	2		20	±	2		11	±	2	
Brainstem	Pathological	3	±	3	ns	12	±	3	=0.032	32	±	3	=0.001	32	±	3	=0.005	16	±	3	ns
	Normal	0	±	4		3	±	3		18	±	3		19	±	4		7	±	4	
Basal ganglia	Pathological	0	±	1	ns	1	±	1	ns	3	±	1	ns	10	±	1	ns	10	±	1	=0.002
	Normal	-1	±	2		-1	±	2		2	±	2		6	±	2		4	±	2	
Limbic structures																					
Amygdala	Pathological	3	±	4	ns	16	±	4	ns	50	±	4	=0.002	65	±	4	=0.002	29	±	4	=0.036
	Normal	1	±	5		6	±	5		33	±	5		45	±	5		16	±	5	

For Review Only

Hippocampus	Pathological	5 ± 6	ns	45 ± 7	=0.021	101 ± 6	=0.013	66 ± 7	ns	24 ± 7	ns
	Normal	4 ± 9		21 ± 8		75 ± 8		45 ± 9		14 ± 9	
Entorhinal cortex (gray matter)	Pathological	11 ± 10	ns	102 ± 10	<0.001	206 ± 10	=0..006	108 ± 11	=0.032	39 ± 11	ns
	Normal	7 ± 13		40 ± 13		162 ± 13		71 ± 14		24 ± 14	
Entorhinal cortex (white matter)	Pathological	1 ± 3	ns	5 ± 3	ns	25 ± 3	=0.006	61 ± 3	<0.001	31 ± 3	=0.005
	Normal	0 ± 4		0 ± 3		13 ± 3		41 ± 4		18 ± 4	
Parahippocampal cortex (gray matter)	Pathological	11 ± 9	ns	89 ± 9	=0.003	173 ± 9	ns	93 ± 10	ns	35 ± 10	ns
	Normal	7 ± 13		45 ± 12		158 ± 12		65 ± 12		21 ± 12	
Parahippocampal cortex (white matter)	Pathological	1 ± 3	ns	9 ± 3	ns	36 ± 3	ns	57 ± 3	=0.006	22 ± 3	ns
	Normal	0 ± 4		2 ± 4		28 ± 4		43 ± 4		14 ± 4	
Thalamus	Pathological	1 ± 2	ns	5 ± 2	ns	11 ± 2	ns	19 ± 2	ns	11 ± 2	=0.019
	Normal	-1 ± 2		1 ± 2		9 ± 2		15 ± 2		5 ± 2	
Insular cortex (gray matter)	Pathological	5 ± 10	ns	76 ± 10	=0.008	167 ± 10	=0.022	98 ± 10	ns	42 ± 10	ns
	Normal	8 ± 13		34 ± 12		132 ± 12		78 ± 13		31 ± 13	
Insular cortex (white matter)	Pathological	0 ± 2	ns	1 ± 2	ns	7 ± 2	ns	25 ± 2	=0.013	18 ± 2	=0.014
	Normal	-1 ± 2		-1 ± 2		4 ± 2		18 ± 2		11 ± 2	
Cingulate cortex (gray matter)	Pathological	4 ± 12	ns	59 ± 12	ns	132 ± 12	ns	90 ± 12	ns	42 ± 12	ns
	Normal	10 ± 14		35 ± 14		120 ± 14		76 ± 14		30 ± 14	
Cingulate cortex (white matter)	Pathological	0 ± 2	ns	1 ± 2	ns	7 ± 2	ns	24 ± 2	ns	16 ± 2	=0.041
	Normal	0 ± 2		0 ± 2		5 ± 2		21 ± 2		11 ± 2	
Optic chiasm	Pathological	24 ± 13	ns	142 ± 13	=0.002	189 ± 13	=0.041	64 ± 14	ns	18 ± 14	ns
	Normal	19 ± 17		77 ± 16		148 ± 16		37 ± 18		7 ± 18	

MWA: Mean ICP wave amplitude. Ns: non-significant differences between groups.

For Review Only
