# Supplementary material for: Impaired glymphatic function in idiopathic intracranial hypertension
Source: Brain Commun. 2021 Mar 21;3(2):fcab043. doi: 10.1093/braincomms/fcab043 (PMC8253298; doi:10.1093/braincomms/fcab043)
Supplement: fcab043_Supplementary_Data [file fcab043_Supplementary_Data.zip › Review_files.zip!/Revision 1-decision letter .pdf]

Revision 1 – Decision Letter

01-Feb-2021

Dear Dr. Eide,

Thank you for the careful revisions of your paper based on reviewer comments. It is a pleasure to accept your manuscript entitled "Impaired glymphatic function in idiopathic intracranial hypertension" for publication in Brain Communications. Any comments made by the reviewer(s) are included below.

The next step is for your paper to go through "First Look". We will contact you again shortly if any final files or formatting changes are required for publication or if we have any queries.

If no changes are needed, your submission will be transferred to our Production Department and you will hear from the Production Editor in due course.

Video abstracts - Brain Communications encourages authors to provide a video abstract to accompany their article. These are hosted on the Brain/Brain Communications YouTube channel. If you are interested in producing a 5-min video abstract to promote your article, then please contact the Editorial Office (brcoms.editorialoffice@oup.com) for further details and guidelines.

Thank you very much for your contribution. On behalf of the Editors of Brain Communications, we look forward to your future contributions to the Journal.

Yours sincerely,

Professor Tara Spires-Jones
Founding Editor, Brain Communications
